# Serotonin limits generation of chromaffin cells during adrenal organ development

**DOI:** 10.1038/s41467-022-30438-w

**Published:** 2022-05-25

**Authors:** Polina Kameneva, Victoria I. Melnikova, Maria Eleni Kastriti, Anastasia Kurtova, Emil Kryukov, Aliia Murtazina, Louis Faure, Irina Poverennaya, Artem V. Artemov, Tatiana S. Kalinina, Nikita V. Kudryashov, Michael Bader, Jan Skoda, Petr Chlapek, Lucie Curylova, Lukas Sourada, Jakub Neradil, Marketa Tesarova, Massimo Pasqualetti, Patricia Gaspar, Vasily D. Yakushov, Boris I. Sheftel, Tomas Zikmund, Jozef Kaiser, Kaj Fried, Natalia Alenina, Elena E. Voronezhskaya, Igor Adameyko

**Affiliations:** 1 Department of Neuroimmunology, Center for Brain Research, Medical University Vienna, Vienna, Austria; 2 Department of Physiology and Pharmacology, Karolinska Institute, Stockholm, Sweden; 3 Koltsov Institute of Developmental Biology, Russian Academy of Sciences, Moscow, Russia; 4 National Medical Research Center for Endocrinology, Moscow, Russia; 5 Federal state budgetary institution “Research Zakusov Institute of Pharmacology” (FSBI “Zakusov Institute of Pharmacology”), Russian Academy of Sciences, Moscow, Russia; 6 Sechenov First Moscow State Medical University (Sechenov University), Moscow, Russia; 7 Max-Delbrück Center for Molecular Medicine (MDC), 13125 Berlin-Buch, Germany; 8 German Center for Cardiovascular Research (DZHK), Partner Site Berlin, Germany; 9 Charité-Universitätsmedizin Berlin, 10117 Berlin, Germany; 10 Institute for Biology, University of Lübeck, 23562 Lübeck, Germany; 11 Department of Experimental Biology, Faculty of Science, Masaryk University, Brno, Czech Republic; 12 International Clinical Research Center, St. Anne’s University Hospital, Brno, Czech Republic; 13 Central European Institute of Technology, Brno University of Technology, Brno, Czech Republic; 14 Unit of Cell and Developmental Biology, Department of Biology, University of Pisa, Pisa, Italy; 15 Center for Neuroscience and Cognitive Systems @UniTn, Istituto Italiano di Tecnologia, Rovereto, Italy; 16 INSERM, Paris Brain Institute, Paris, France; 17 Severtsov Institute of Ecology and Evolution, Russian Academy of Sciences, Moscow, Russia; 18 Department of Neuroscience, Karolinska Institute, Stockholm, Sweden

**Keywords:** Developmental biology, Embryogenesis, Paediatric cancer, Emotion, Endocrinology

## Abstract

Adrenal glands are the major organs releasing catecholamines and regulating our stress response. The mechanisms balancing generation of adrenergic chromaffin cells and protecting against neuroblastoma tumors are still enigmatic. Here we revealed that serotonin (5HT) controls the numbers of chromaffin cells by acting upon their immediate progenitor “bridge” cells via 5-hydroxytryptamine receptor 3A (HTR3A), and the aggressive HTR3A^high^ human neuroblastoma cell lines reduce proliferation in response to HTR3A-specific agonists. In embryos (in vivo), the physiological increase of 5HT caused a prolongation of the cell cycle in “bridge” progenitors leading to a smaller chromaffin population and changing the balance of hormones and behavioral patterns in adulthood. These behavioral effects and smaller adrenals were mirrored in the progeny of pregnant female mice subjected to experimental stress, suggesting a maternal-fetal link that controls developmental adaptations. Finally, these results corresponded to a size-distribution of adrenals found in wild rodents with different coping strategies.

## Abstract (web summary)

Adrenal glands are major organs regulating stress response., Melnikova et al., show that local release of serotonin limits adrenalin-producing cell number during rodent development, a mechanism which has implications for neuroblastoma development and stress-related maternal effects transmitted to progeny.

## Introduction

Adrenal glands are key hormonal regulators in our body, as they control major physiological processes of our daily life, and homeostasis cannot be maintained without their normal function. The structure of adrenal glands includes the cortical matter, consisting of cells that produce steroid hormones, and the centrally positioned medulla (adrenal medulla—AM), which orchestrates the response of our body to stress by releasing catecholamines (adrenaline and noradrenaline). Quite remarkably, there is another similar catecholamine-producing organ transiently present in our body, namely the Organ of Zuckerkandl (ZO)^1^. The ZO eventually disappears during the first years of human life. Chromaffin cells represent the major catecholamine-producing cell type in the AM and in ZO (together called chromaffin organs).

Despite the importance for our physiology, the key details of adrenal gland development remain unclear. These details are important not only for the adrenal gland engineering attempts or for understanding associated congenital abnormalities, but also for coping with tumors arising from sympathoadrenal lineage, namely neuroblastoma, pheochromocytoma, and paraganglioma. According to a recent paradigm, tumor cells exploit and re-play developmental programs to elicit intra-tumoral plasticity and resist treatment^2^. In addition, the good knowledge of developmental steps and molecular profiles assists better classification of tumors and helps to pinpoint the tumor-initiating cell types using transcriptional similarity of malignant cells to particular developmental cell states^3–5^.

In line with this reasoning, recent studies showed that the initial stages of chromaffin cell development depend on the recruitment of local nerve-associated Schwann cell precursors (SCPs), which turn into a short-living transient population of “bridge” cells that rapidly transitions towards mature chromaffin cells in mouse and human embryos^6–9^. This finding complicated the old picture of adrenal development (where migratory neural crest cells immediately generate chromaffin tissues), and raised a series of questions regarding the control of the number of chromaffin progenitors operating during the differentiation steps. These “bridge” cells are characterized by the expression of *Htr3a*^6,8^—a gene encoding for a subunit of HTR3 receptor to serotonin (5-hydroxytryptamine, 5HT). Based on that, 5HT was recently hinted to be a part of the mechanism related to the development of adrenal medulla^6^.

More generally, 5HT is crucial for the embryonic development^10^ and for postnatal growth in the animal kingdom^11–13^, including the formation of the nervous system^14–16^. Moreover, 5HT is one of the main players shaping mood, fight-or-flight stress response, and aggressive behavior in mammals^17^. One of the key connection between embryonic development, chromaffin organs and 5HT comes from studies of animal domestication. Domesticated animals have higher levels of 5HT and less catecholamine-triggered aggressive behaviors^18^. Genetic differences between wild and tame animals of the same species include genes encoding for the enzymes of 5HT synthesis and degradation^19^. Moreover, the adrenal glands of domesticated animals are smaller than their wild relatives^20,21^. Therefore, behavioral differences can be attributed to variations in 5HT and catecholamine systems and to the size of the respective endocrine organs, particularly the adrenal glands, which are shaped during embryonic development.

In this study, by analysing cellular composition and cell dynamics in the developing adrenal medulla and ZO, we show a 5HT-mediated regulatory negative feedback loop between chromaffin cells and their immediate precursor “bridge” cells. In line with this, we demonstrate that neuroblastoma cell lines with high expression of HTR3A are more tumorigenic and respond to HTR3A agonists with reduced proliferation rate. Finally, we discover that high levels of mother-derived 5HT affect the development of embryonic adrenal medulla in a systemic way, being possibly involved in transmission of environmental signals and stress-related states from pregnant mother to her progeny. Indeed, we find that maternal mild stress induces smaller adrenal medullae in the progeny of stressed animals. Furthermore, the wild rodent population demonstrate a natural distribution of adrenal medulla sizes correlated with their preferred individual lifestyles. Taken together, these results support a major ecological and evolutionary role of the mechanisms controlling the development of adrenal glands and, in particular, chromaffin cells via a 5HT pathway.

## Results

### 5HT-secreting and 5HT-sensitive cells in chromaffin organs

To address the role of 5HT signaling in adrenal gland development, we re-analyzed the expression of related enzymes and receptors using previously published single-cell transcriptomics dataset of chromaffin and sympathetic development at E12.5 and E13.5 stages^6^ (Supplementary Fig. 1). As shown earlier by Furlan et al., chromaffin cells originate from SCPs and the differentiation progresses through the transitory “bridge” population. Thus “bridge” cells are immediate progenitors of chromaffin cells (Supplementary Fig. 1a). According to our analysis at E12.5, *Htr3a/3b* (encoding 5HT-receptor 3A/3B) are strongly expressed in the population of “bridge” cells and are only sporadically expressed in cells from other clusters. At E13.5, *Htr3a/3b* specifically marks the “bridge” cell population and also appears to be present in a minor portion of sympathoblasts (Supplementary Fig. 1b). To check if *HTR3A* is expressed in human adrenal medulla during development, we isolated adrenal glands at weeks 5-to-7 and subjected them to single-cell transcriptomics analysis with Chromium 10X approach. The results showed the sparse expression of *HTR3A* in “bridge” cells and rather consistent expression in sympathoblasts (Supplementary Fig. 2a, b). Although we detected only 6 *HTR3A*^+^ cells in a “bridge” population, the statistical test supports the significance of this find (Supplementary Fig. 2c). At the same time, this suggests low expression level of *HTR3A*, which we experimentally validated with RNAscope in situ hybridisation on slices of week 6 and week 8 human adrenal tissue (Supplementary Fig. 2d). Indeed, if the expression of *HTR3A* is truly present, it is weak and at the border of detection, which leaves the question about the role of *HTR3A* in human “bridge” cells open. Consistently, the data from other groups show almost absent expression of *HTR3A* from SCPs and “bridge” cells in developing human adrenal medulla despite its clear presence in sympathoblasts^22^. Again, a question of whether *HTR3A* is sufficiently present in “bridge” cells of human adrenal glands requires further investigation with more sensitive methods.

5HT, the ligand activating the ion channel receptor formed by HTR3A/3B proteins, can be supplied through the embryonic bloodstream, although it might be also produced locally by other cells of the developing adrenal medulla (AM). Thus, we explored different cell types in AM for the presence of enzymatic cascades necessary to produce local 5HT. It turned out that nearly all cells in AM express *Ddc* gene encoding the enzyme responsible for decarboxylating 5HTP (5-hydroxytryptophan) into 5HT, and *Maoa* gene encoding the enzyme responsible for monoamine degradation. At the same time, the synthesis of 5HT from tryptophan does not take place in AM cells, as most cells do not express the *Tph1* and *Tph2* genes at E12.5 and E13.5 (Supplementary Fig. 1c). Thus, the majority of immature and mature chromaffin cells, and a subset of SCPs and “bridge” cells are capable of producing 5HT from the chemical precursor 5HTP, but not from tryptophan. In addition to this, at E12.5, chromaffin cells are positive for *Slc29a4*, which encodes Plasma Membrane Monoamine Transporter (PMAT)—a non-selective transporter responsible for pumping 5HT inside the cells (Supplementary Fig. 1d). Therefore, embryonic chromaffin cells are capable of taking 5HT from the bloodstream, especially given that the adrenal glands are heavily vascularized. Moreover, chromaffin cells and sympathoblasts express *Slc18a1* and *Slc18a2*, which encode for VMAT1 and VMAT2, non-selective vesicular monoamine transporters responsible for the storage of monoamines in intracellular secretory vesicles. Therefore, chromaffin cells possess the necessary molecular machinery to uptake, synthesize, and secrete 5HT, and they originate from the 5HT-sensitive HTR3A^+^ “bridge” cells.

To characterize the local cell type composition and to assess the physical contacts between different cell types in AM and ZO, we took advantage of a standard combination of immunohistochemical markers (SOX10 for SCPs, tyrosine hydroxylase (TH) for chromaffin cells, and 5HT) together with genetically modified *Htr3a*^*EGFP*^ mice in order to visualize “bridge” cells and their newly-differentiated progeny^23^. Because EGFP can be retained in cells up to 48 h after the cease of active expression^24^, we additionally visualized “bridge” cells by *Htr3a* mRNA in situ hybridization, which revealed a proportion of *Htr3a*^EGFP+^ cells actively expressing *Htr3a* mRNA in developing AM at the time of the observations (Supplementary Fig. 3a). This result shows that *Htr3a*^EGFP+^ cells negative for *Htr3a* mRNA (post-bridge cells) rapidly differentiate into TH^+^ chromaffin cells. No chromaffin cells were found positive for *Htr3a* mRNA (Supplementary Fig. 3b). Therefore, the *Htr3a*^*EGFP*^ mouse strain can be used to trace the transition from the intermediate “bridge” cell population to the chromaffin cells by assessing the proportions of TH^−^*/Htr3a*^EGFP+^ “bridge” cells, TH^+^*/Htr3a*^EGFP+^ early chromaffin cells and TH^+^/*Htr3a*^EGFP−^ mature chromaffin cells.

**Fig. 1:**
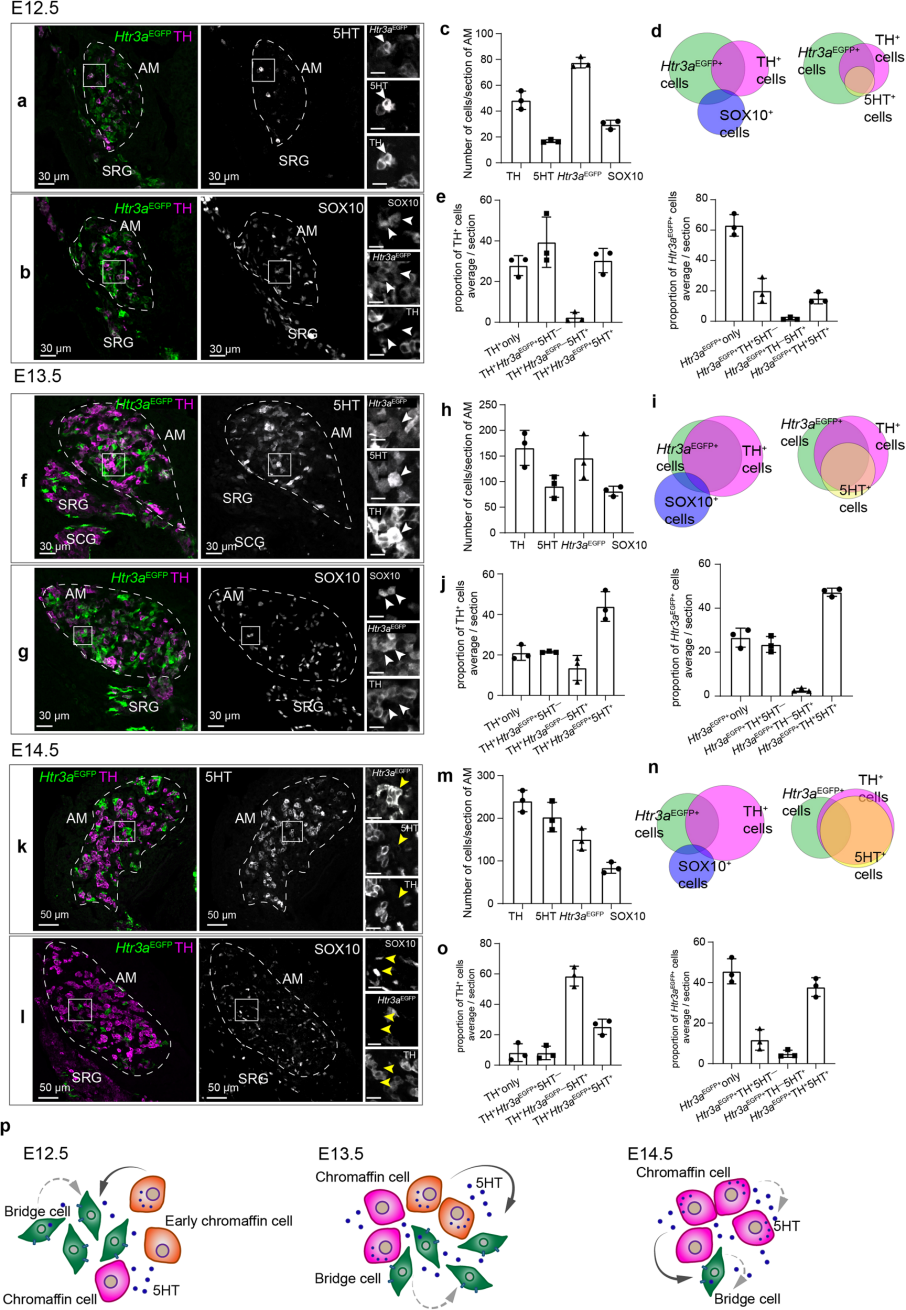
5HT-sensitive “bridge” cells and 5HT-producing chromaffin cells are present together in developing adrenal glands. **a**, **b** Transversal section of adrenal gland of *Htr3a*^*EGFP*+/−^ embryos immunostained for TH (marker chromaffin cells and sympathoblasts), EGFP (indicating expression of *Htr3a*), and 5HT (5-hydroxytryptamine, serotonin) (**a**), and for SOX10 (marker of SCPs) (**b**) at E12.5. White arrows point at TH^+^/*Htr3a*^EGFP+^/5HT^+^ (**a**) and SOX10^+^/*Htr3a*^EGFP+^/TH^−^ (**b**) cells, indicating formation of “bridge” cells and their differentiation into 5HT^+^ early chromaffin cells. **c** Cell numbers at E12.5. **d** Venn diagrams of *Htr3a*^EGFP+^, TH^+^, SOX10^+^ (left), and 5HT^+^ (right) cells at E12.5. **e** Proportions of TH^+^ cells also positive for *Htr3a*^EGFP^ and 5HT (left), and proportions of *Htr3a*^EGFP+^ cells with TH and 5HT signal (right). **f**, **g** Immunohistochemistry on the transversal section of adrenal glands of *Htr3a*^*EGFP*+/−^ embryos stained for TH, EGFP, 5HT (**f**) and for SOX10 (**g**) at E13.5. White arrows point at TH^+^/*Htr3a*^EGFP+^/5HT^+^ cells in (**f**) and SOX10^+^/*Htr3a*^EGFP+^/TH^−^ cells in (**g**) at E13.5. **h** Cell numbers at E13.5. **i** Venn diagrams of *Htr3a*^EGFP+^, TH^+^, SOX10^+^ (left), and 5HT^+^ (right) cells at E13.5. **j** Proportions of TH^+^ cells positive for *Htr3a*^EGFP^, 5HT (left) and proportions of *Htr3a*^EGFP+^ cells positive for TH, 5HT (right). **k**, **l** Transversal sections of adrenal glands from *Htr3a*^*EGFP*+/−^ embryos stained for TH, EGFP, and 5HT in (**k**), and for SOX10 in (**l**) at E14.5. Yellow arrows point at TH^−^/*Htr3a*^EGFP+^/5HT^−^ cells (**k**) and SOX10^+^/*Htr3a*^EGFP−^/TH^−^ cells (**l**) indicating the end of “bridge” differentiation, SOX10^+^ represent supporting glial cells. **m** Cell numbers at E14.5. **n** Venn diagrams of *Htr3a*^EGFP+^, TH^+^, SOX10^+^ (left), and 5HT^+^ cells (right) at E14.5. **o** Proportions of TH^+^ cells positive for *Htr3a*^EGFP^, 5HT (left), and proportions of *Htr3a*^EGFP+^ cells positive for TH and 5HT (rigth). **p** Schematic representation of the proposed paracrine/autocrine regulation: chromaffin cells release 5HT activating HTR3A receptors on the surface of “bridge” cells (solid lines). Note that few “bridge” cells produce 5HT and can stimulate “bridge” population in an autocrine mode as well as some chromaffin cells can sense 5HT with other 5HT receptors (dashed lines). Scale bars for the insets are 10 µm. Quantification is presented as mean ± SD, biological *n* = 3. Adrenal medulla (AM) is outlined by the dashed line in all sections. SRG: suprarenal ganglion, SCG: sympathetic chain ganglion.

AM and ZO are dynamically developing organs, and, therefore, the proportion of cells building AM and ZO changes between E12.5 and E14.5 towards more mature cell types (Fig. 1). Indeed, TH^−^/*Htr3a*^EGFP+^ “bridge” cells compose most of the AM and ZO at E12.5 (Fig. 1a–d, Supplementary Fig. 4a–d), and they rapidly differentiate into TH^+^*/Htr3a*^EGFP+^ early chromaffin cells and TH^+^/*Htr3a*^EGFP−^ mature chromaffin cells, composing most of the AM and ZO at E13.5 and E14.5 (Fig. 1f–o, Supplementary Fig. 4f–o). We validated the active transition from SCPs to “bridge” cells by visualizing the presence of SOX10^+^/*Htr3a*^EGFP+^ cells at E12.5 and E13.5 (Fig. 1b, g insets, Supplementary Fig. 4), as well as by assessing *Htr3a* mRNA expression at E13.5 (Supplementary Fig. 3a).

Next, our analysis revealed that already at E12.5, nearly 32% of early and mature chromaffin cells are 5HT^+^ in the AM (Fig. 1e). Soon after, around 58% and 77.8% of chromaffin cells became 5HT^+^ in the AM at E13.5 and E14.5 correspondingly (Fig. 1f–o). At the same time, SCPs did not show 5HT immunoreactivity (Supplementary Fig. 5a), and only 1.7–5.1% of all *Htr3a*^EGFP+^ cells were *Htr3a*^EGFP+^/TH^−^/5HT^+^ (5HT-positive “bridge” cells) at E12.5–E14.5 (Fig. 1e, j, o). In ZO the proportion of 5HT^+^ cells among TH^+^ and *Htr3a*^EGFP+^ cells followed a similar pattern (Supplementary Fig. 4). Therefore, chromaffin cells contribute most of the local 5HT to the surrounding and neighboring cell types in the adrenal gland. In line with these observations, at E12.5–E13.5, *Htr3a*^EGFP+^/TH^−^ “bridge” cells (sensitive to 5HT) are intermingled with chromaffin cells in the primordium of AM and ZO (Fig. 1a, e, Supplementary Fig. 4a–h), and are susceptible to 5HT generated by neighboring chromaffin cells. Based on this, we propose a mechanism of a paracrine control, where chromaffin cells release 5HT acting on neighboring “bridge” cells, with some contribution of the autocrine regulation, where few 5HT^+^ “bridge” cells act on themselves and other HTR3A^+^ “bridge” cells in their vicinity. As the SCPs and few chromaffin cells express other receptors to 5HT, additional modes of local autocrine and paracrine control might be also present (Fig. 1p).

When the differentiation of chromaffin cells slows down around E14.5 (E13.5 in ZO)^6,8^, the proportion of SOX10^+^/*Htr3a*^EGFP+^ cells decreases gradually (from 6.3% at E13.5 to 4.7% at E14.5), as the SOX10^+^ SCPs engage into the “bridge” fate at a decreasing rate (Fig. 1k–o, Supplementary Fig. 4f–o). Consistently, after E14.5, the majority of SOX10^+^ cells of AM and ZO are supporting glial cells and immature Schwann cells, as reported in the literature^25^.

Thus, the observed transitions between SCPs, “bridge” and chromaffin cells suggest that the key 5HT-mediated regulatory phase occurs predominantly in a limited time window during chromaffin organ development (E11.5–E14.5). This is substantiated by the fact that *Htr3a*^EGFP+^/TH^+^/5HT^+^ early chromaffin cells (reflecting the transition from *Htr3a*^EGFP+^ cells to chromaffin cells) are observed already at E12.5 within the AM (Fig. 1a, f insets), whereas at E14.5 the proportion of these cells drops two-fold in comparison with E13.5 (Fig. 1j, o). Moreover, at E14.5, a subset of TH^+^ chromaffin cells in AM undergoes further functional specialization as shown by the onset of expression of *Pnmt* gene encoding phenylethanolamine N-methyltransferase (PNMT) (Supplementary Fig. 5b), the enzyme responsible for converting noradrenaline to adrenaline. Even though the contribution of SCPs to “bridge” cells and their differentiation towards early chromaffin cells peaks at E13.5 and is reduced significantly after E15.5 in AM, in E14.5 ZO 90% of cells are represented by newly-generated *Htr3a*^EGFP+^/TH^+^/5HT^+^ early chromaffin cells (Supplementary Fig. 4o). Importantly, during this time window and up to postnatal day 14 (P14) (the latest stage we checked), chromaffin cells in AM and ZO maintain 5HT-immunoreactivity (Supplementary Fig. 5c). Thus, chromaffin cells in mice are capable of releasing both 5HT and catecholamines during embryonic development and later during postnatal life^26–30^ being involved into a system of cell number control, which we specify below.

### High 5HT causes developmental reduction of chromaffin organs

**Fig. 2:**
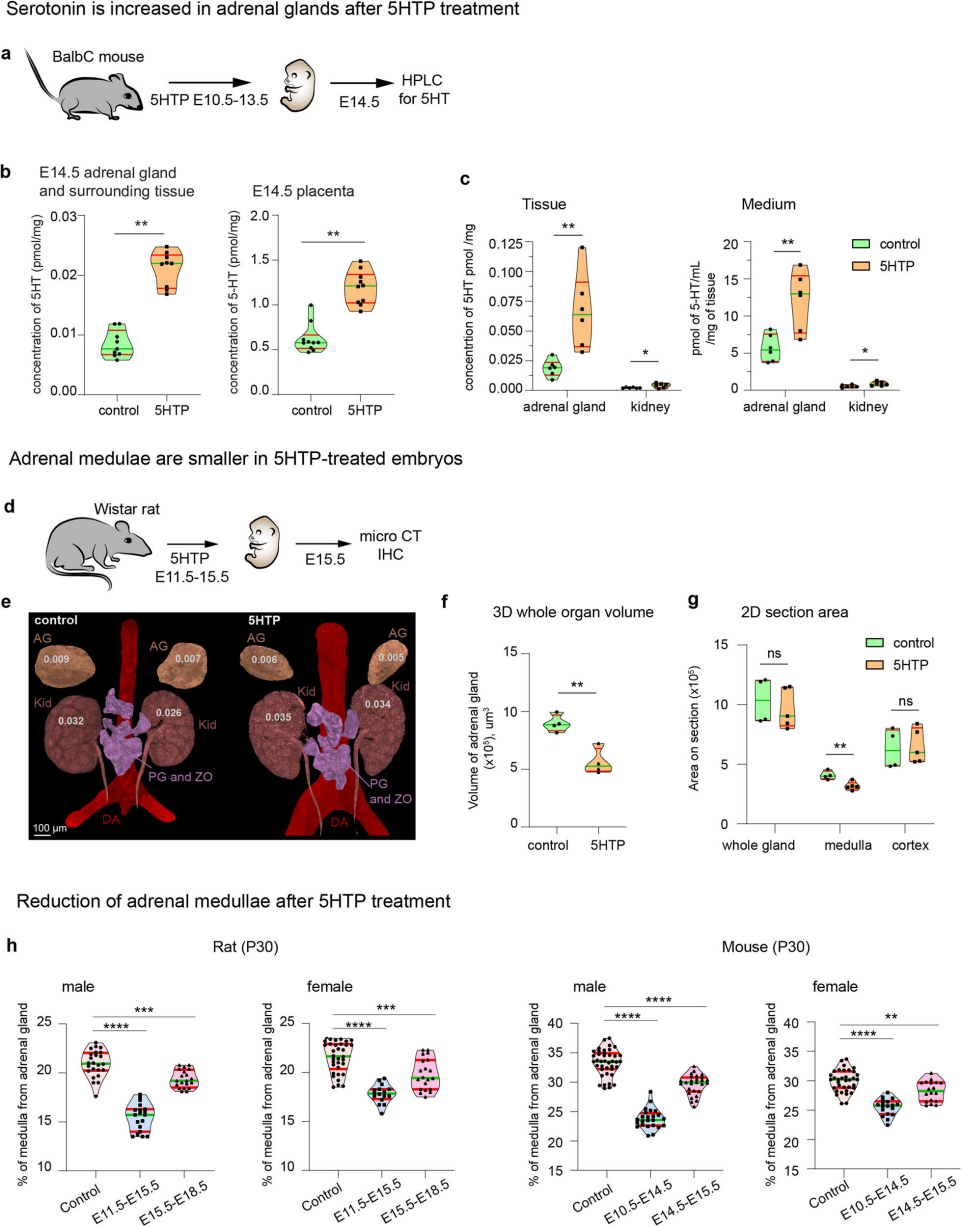
Administration of 5HTP, the immediate precursor of 5HT, to pregnant rodents reduces adrenal medulla in the progeny. **a** 5HTP was administered to pregnant BalbC mice at E10.5–E13.5. The embryos are collected at E14.5 for the HPLC-based analysis of 5HT. **b** Concentration of 5HT in the adrenal glands and surrounding tissues (left) and placentas (right) at E14.5 after 5HTP administration at E10.5–E13.5 stage. Unpaired double-sided t-test *p*-value ** < 0.001, biological *n* = 9. **c** Adrenal glands from E14.5 embryos release 5HT into the surrounding medium measured by HPLC-ED. Note: kidneys, used as a control tissue, do not release 5HT. Unpaired double-sided *t*-test *p*-value * < 0.05, **= 0.005, biological *n* = 6. **d** 5HTP was administered to pregnant Wistar rats at E11.5–E15.5 followed by embryo collection at E15.5 and analysis by X-ray computed microtomography (microCT). **e** microCT X-ray reconstruction of adrenal glands and kidney volume from E15.5 rat embryos obtained from females in control and 5HTP-treated groups. Volumes of the organs in µm^3^ (×10^5^). **f** Adrenal gland volume normalized to the volume of adjacent kidneys. Biological *n* = 4. Unpaired double-sided t-test *p*-value ** < 0.005. **g** Section areas of adrenal gland, adrenal medulla and adrenal cortex based on immunohistochemistry analysis with anti-TH immunostaining. Unpaired double-sided *t*-test *p*-value ns > 0.05, * < 0.05, biological *n* = 4 (control), 5 (5HTP). **h** Prenatal 5HTP exposure at the time of “bridge” cell differentiation (E11.5–E15.5 in rats and E10.5–E14.5 in mice) causes the decrease of postnatal adrenal medulla size in P30 animals, which was greater than decreased induced by 5HTP exposure after the time of “bridge” cell differentiation (E15.5–E18.5 in rats and E14.5–E15.5 in mice).  Green line in all violin plots—the median, red lines—quartiles. One-way ANOVA test with Dunnett’s multiple comparison test **p = 0.0017, ***p = 0.0002, ****p < 0.0001, biological *n* (adrenal gland) = 24 (control male rat), 19 (E11.5–E15.5 male rat), 20 (E15.5–E18.5 male rat), 35 (control female mice), 16 (E11.5–E15.5 female rat), 20 (E15.5–E18.5 female rat), 40 (control male mice), 24 (E10.5–E14.5 male mice), 27 (E14.5–E15.5 male mice), 35 (control female mice), 18 (E10.5–E14.5 and E14.5–E15.5 female mice). For all experiments normality is checked with Shapiro–Wilk test. AG: adrenal gland, Kid: kidney, PG and ZO: paraganglia and organ of Zuckerkandl, DA: dorsal aorta.

To reveal the effects of 5HT on the developing AM, we performed a gain-of-function experiment by administering its biochemical precursor, 5HTP, to pregnant rats (Fig. 2a). The administration of 5HTP enables to increase the physiological concentrations of 5HT in embryos without disruption of pregnancy caused by direct 5HT administration^31^. Moreover, 5HTP is converted to 5HT by the placenta^14^. As expected, administration of 5HTP to pregnant females led to a significant increase of 5HT concentration in the placenta and trunks of E14.5 embryos (Fig. 2b), and enhanced the release of 5HT by the fetal adrenal glands (Fig. 2c), as measured by HPLC-ED. Thus, the administration of 5HTP to pregnant rodent females causes a stable and physiological increase of 5HT concentration in embryos, as previously shown for other tissues including the uterus^32,33^.

Pregnant Wistar rats received 5HTP during E11.5–E15.5 stages, and the adrenal glands of E15.5 embryos were investigated in 3D by micro computerized tomography (microCT) (Fig. 2d). We observed a 37.2% reduction of the mean volume of adrenal glands in embryos upon 5HTP treatment (Fig. 2e, f). Immunohistochemical analysis of adrenal glands of littermates revealed a significant reduction of the AM, whereas the area of the adrenal cortex was similar in control and experimental offspring (Fig. 2g). The volume of kidneys in treated embryos did not change, confirming the lack of general developmental delay.

To make sure that the effect of 5HT on the AM size is consistent in a different rodent model, we analyzed the adrenal glands of mice and obtained consistent results (Fig. 2h). 5HTP treatment of pregnant rats and mice during the active differentiation of “bridge” cells into chromaffin cells resulted in a long-lasting reduction of AM size in embryonic and postnatal life in both species. Administration of 5HTP outside this critical time window led to a less pronounced effect (Fig. 2h). Thus, we were able to influence the size of the adrenal glands in rodent offspring through the elevation of 5HT levels in pregnant animals during a limited developmental time window corresponding to the peak of chromaffin cell generation.

**Fig. 3:**
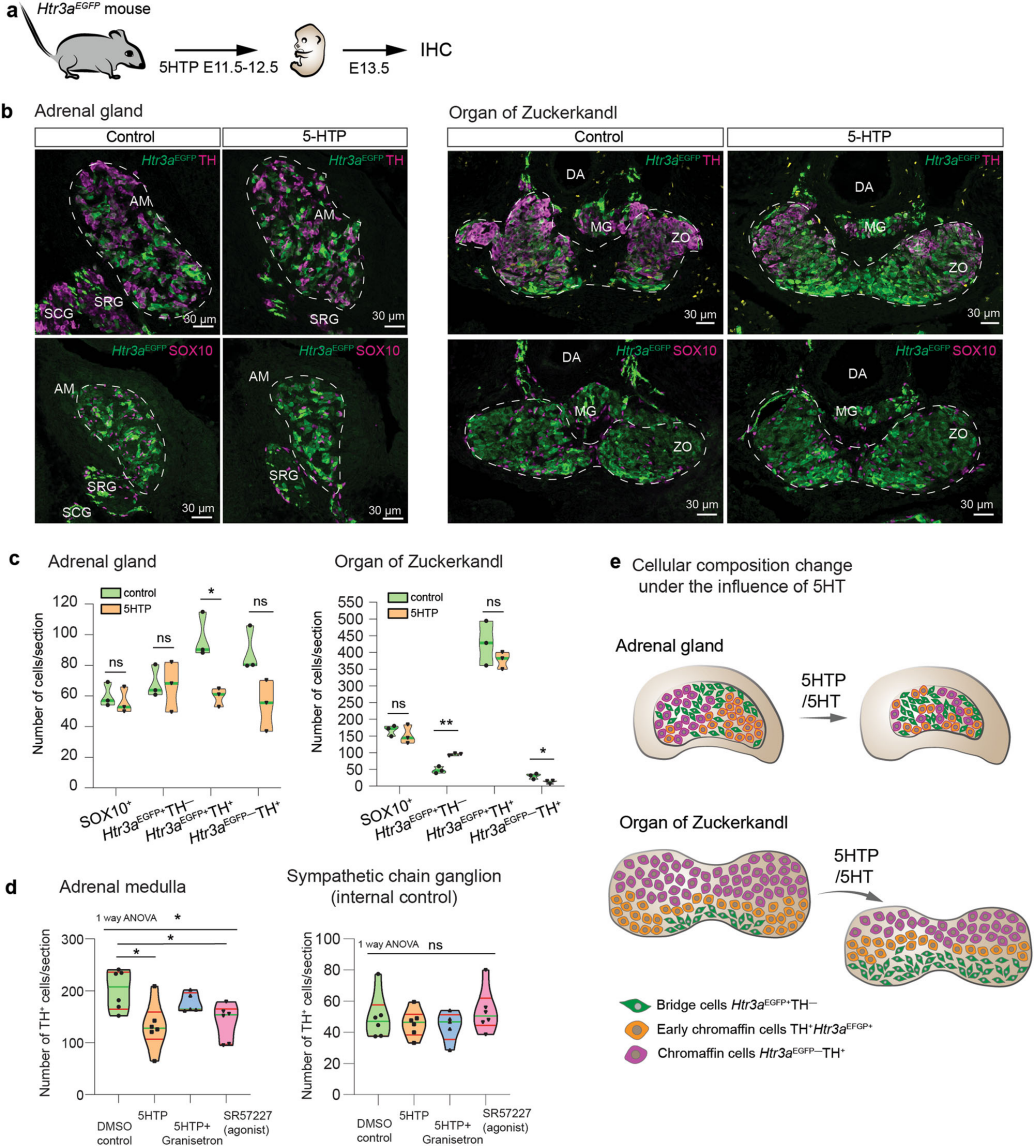
Administration of 5HTP to pregnant females influences chromaffin and “bridge” cells in embryonic chromaffin organs. **a** 5HTP was administered to pregnant *Htr3a*^*EGFP*+/−^ females, which was followed by the analysis of embryonic adrenals at E13.5 by immunohistochemistry. **b** Transversal section through the adrenal glands (left) and Organ of Zuckerkandl (right) immunostained for TH, EGFP (indicating expression of *Htr3a*), and SOX10. The sections were obtained from E13.5 embryos collected from *Htr3a*^*EGFP*+/−^ females from control and 5HTP-treated groups. **c** Cell numbers in adrenal medulla (left) and Organ of Zuckerkandl (right) at E13.5 in control and treated groups. Note that early chromaffin cells (*HTR3A*^EGFP+^/TH^+^) and mature chromaffin cells (*HTR3A*^EGFP−^/TH^+^) decrease, whereas SCPs (SOX10^+^) and “bridge” cells (*HTR3A*^EGFP+^/TH^−^) do not change in the treated group vs control in the adrenal medulla. At the same time, “bridge” cells (*HTR3A*^EGFP+^/TH^−^) accumulate and mature chromaffin cells (*HTR3A*^EGFP−^/TH^+^) decrease in the Organ of Zuckerkandl in the treated group. Cell number are presented as violin plots, where the green line indicates median, biological *n* = 3, Shapiro–Wilk test for normality and unpaired double-sided t-test *p*-value ns > 0.05, * < 0.05, ** < 0.002. **d** Cell number in adrenal medulla and sympathetic chain ganglia across four groups of E13.5 embryos treated during E11.5–E12.5 with: DMSO (control), 5HTP, 5HTP and Granisetron (HTR3A antagonist), SR57227 (HTR3A agonist). Note that cell numbers change in the adrenal medulla, but not in the sympathetic chain ganglion. Biological *n* = 6 (DMSO, 5HTP, SR57227), 5 (5HTP + Granisetron), one-way ANOVA AM *p*-value *0.0135, followed by Tukey multiple comparison test DMSO vs 5HTP *p*-value *0.0189, DMSO vs SR57227 *p*-value *0.0406; ANOVA SCG *p*-value ns 0.6265. **e** Changes in size and cellular composition in E13.5 adrenal medulla and Organ of Zuckerkandl in mice under the influence of increased 5HT (E11.5–E12.5). Cell numbers are presented as violin plots, the green line—median and the red lines—quartiles. Adrenal medulla (AM) and Organ of Zuckerkandl (ZO) are outlined by the dashed line in sections. SRG: suprarenal ganglion, SCG: sympathetic chain ganglion, MG: mesenteric ganglion.

To pinpoint the specific medullary population affected by the increased levels of 5HT in developing AM and ZO, we repeated the 5HTP treatment in the transgenic *Htr3a*^*EGFP*^ mice. In order to target the same consistent stages across species, 5HTP was administered to pregnant mice at E11.5–E12.5, corresponding to E13.0–E15.0 in rats^34^ (Fig. 3a). As a result, at E13.5, the numbers of early chromaffin cells (TH^+^*/Htr3a*^EGFP+^) and mature chromaffin cells (TH^+^*/Htr3a*^EGFP−^) were reduced by 39.1% and 38.8%, respectively (difference between mean values of control and 5HTP groups), in AM of the embryos from the treated mice (Fig. 3c). We did not detect a significant change in “bridge” cells number at E13.5. In the ZO, the number of “bridge” cells (*Htr3a*^EGFP+^/TH^−^) was twice higher, and the number of mature chromaffin cells (TH^+^*/Htr3a*^EGFP−^) was reduced (Fig. 3c). Schwann cell precursors (SOX10^+^) cell numbers were unaffected in both chromaffin organs (Fig. 3c).

To control for the systemic effect, we checked the cells in the sympathetic chain ganglia (SCG), which are directly derived from the migratory neural crest cells and do not transition through a “bridge” stage^6,35^. Thus, SCG served as an internal control for the changes in size of chromaffin organs. 5HTP administration did not influence the size or cellular composition of SCG (Supplementary Fig. 6). Therefore, the reduction of chromaffin organs is specific and occurs at the expense of chromaffin cells.

To confirm that the observed phenotype emerged due to the activation of HTR3A, we used SR57227 hydrochloride, a potent and selective HTR3A agonist. Administration of SR57227 to pregnant mice at E11.5 and E12.5 caused a 30.6% reduction (difference between mean values of DMSO control and SR57227 groups) of early and mature TH^+^ chromaffin cells in AM at E13.5 (Fig. 3d). This is similar to the effect caused by 5HTP administration to pregnant mice during the same developmental time. To rescue the phenotype caused by the elevated 5HT level, we co-administered granisetron, a HTR3A antagonist, in combination with 5HTP. In this condition, the number of TH^+^ cells in AM turned out to be similar (Fig. 3d), whereas the administration of 5HTP alone caused a 34.5% reduction (difference between mean values of DMSO control and 5HTP groups) of the chromaffin cells in AM. The SCG (representing a control tissue with different genesis) showed the same numbers of TH^+^ sympathoblasts in all groups (Fig. 3d). Therefore, the HTR3A activation mediated 5HT-dependent signaling in the development of the chromaffin cell lineage and caused the decrease of chromaffin cells in AM and ZO upon 5HTP treatment.

**Fig. 4:**
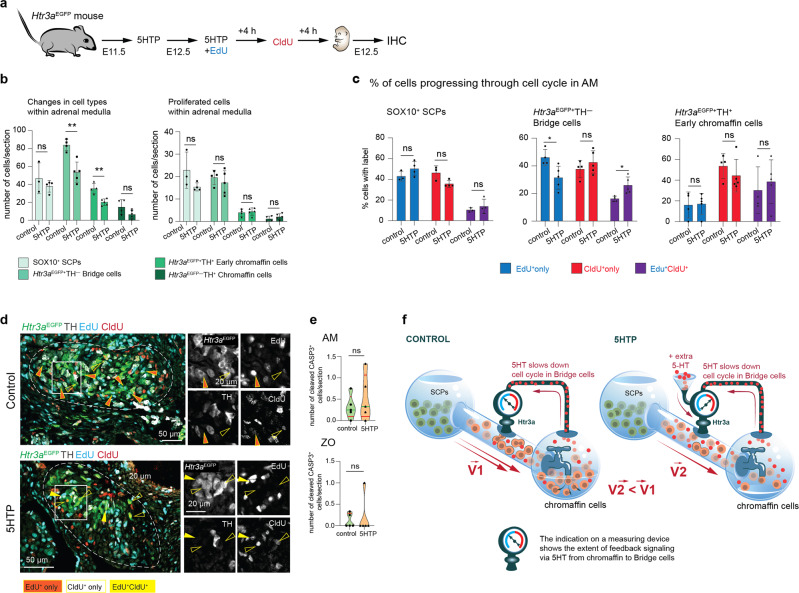
Prenatal 5HT influences cell cycle length of the “bridge” progenitors in embryonic adrenal glands. **a** Pregnant *Htr3a*^EGFP+/−^ females were administered 5HTP at E11.5; at E12.5, females received 5HTP together with 5-Ethynyl-2′-deoxyuridine (EdU); in 4 h females received 5-Chloro-2′-deoxyuridine (CldU); embryos were harvested in 4 h after CldU injection at E12.5. This allows identifying the proportions of cells, which incorporated EdU^+^ only, CldU^+^ only, or both thymidine analogues (EdU^+^CldU^+^). **b** Numbers of all cells (left) and numbers of proliferated cells (right) in populations of SCPs, “bridge” cells, early chromaffin cells, and mature chromaffin cells at E12.5 under the influence of 5HTP in adrenal medulla. Shapiro–Wilk test for normality and unpaired double-sided *t*-test *p*-value ns > 0.05, ** < 0.002, biological *n* = 3 (control SCPs), 4 (control other cell types), 4 (5HTP SCPs), 5 (5HTP other cell types). **c** Proportions of EdU^+^ only, CldU^+^ only, and EdU^+^CldU^+^ cells in populations of SCPs, “bridge” cells, and early chromaffin cells at E12.5 under the influence of 5HT in adrenal medulla. Note that the cell cycle lengthened in “bridge” cells in the treated condition, as there are more EdU^+^CldU^+^ “bridge” cells. Shapiro–Wilk test for normality and unpaired double-sided *t*-test *p-*value ns > 0.05, * < 0.05, biological *n* = 3 (control SCPs), 4 (control other cell types), 4 (5HTP SCPs), 5 (5HTP other cell types). **d** Transversal sections of adrenal glands from *Htr3a*^*EGFP*+/−^ embryos immunostained for TH, EGFP (indicating the expression of *Htr3a*), EdU, and CldU. Arrowheads point at EdU^+^ only, CldU^+^ only, and EdU^+^CldU^+^ cells. **e** Numbers of cleaved CASP3^+^ cells in adrenal medulla and Organ of Zuckerkandl. Note that the number of cleaved CASP3^+^ cells is exceptionally low. Cell numbers are presented in violin plots, the green line indicates median and the red lines are quartiles. Shapiro–Wilk test for normality and unpaired double-sided *t*-test (AM) and Mann–Whitney test (ZO), *p*-value ns > 0.05, biological *n* = 5. **f** Proposed mechanism of the paracrine/autocrine regulation of a cell cycle length in HTR3A^+^ “bridge” precursors by 5HT-releasing chromaffin cells in the developing adrenal medulla.

To further investigate the reasons for the observed reduction in chromaffin cell numbers, we checked the dynamics of cell cycle in various populations of cells in the chromaffin lineage. For this purpose, we administered consecutively 5-ethynyl-2′-deoxyuridine (EdU) and 5-chloro-2′-deoxyuridine (CldU) thymidine analogues at E12.5 with 4-h intervals to *Htr3a*^*EGFP*^ mice treated with 5HTP (Fig. 4a). At the time of CldU injection, EdU is not available for cells due to its rapid pharmacokinetics^36^. This approach allowed calculating the proliferation rate as well as the proportion of Edu^+^Cldu^−^, Edu^−^Cldu^+^, and Edu^+^Cldu^+^ cells.

Despite the overall numbers of proliferating (incorporating one or both thymidine analogues) cells among SCPs, “bridge” cells, early chromaffin cells or mature chromaffin cells did not show a significant change in comparison with control (Fig. 4b), the length of a cell cycle in “bridge” cells appeared significantly increased in the 5HTP-treated group (Fig. 4c, d, Supplementary Fig. 7a). Delayed cell cycle progression resulted in the reduced number of “bridge” cells observed at this stage, soon after the injection (Fig. 4b). Such reduction of “bridge” cells was not observed at E13.5, due to a fast turnover of “bridge” cells and subsequent recruitment of new “bridge” cells from SCPs. Moreover, 5HTP and 5HT are rapidly depleted after the injection-dependent concentration peak due to pharmacokinetics. At the same time, the dynamics of cell cycle progression in SCPs and early chromaffin cells in AM did not change in control vs treatment group (Fig. 4c). The majority of mature chromaffin cells were negative for EdU or CldU, as they temporarily exit cell cycle in agreement with previous studies^37^. Due to fast differentiation of chromaffin cells from progenitors in 14 h^7^, reduction of the progenitor pool has a major effect of chromaffin cell population. A majority of chromaffin cells do not proliferate at E12.5–E13.5 and are unable to compensate for the loss.

Contrary to the AM, in the E12.5 ZO, we observed a lower proportion and reduced absolute numbers of SOX10^+^ SCPs incorporating EdU only in the 5HTP-treated group (Supplementary Fig. 7), which suggests another mechanism of cell number control or delayed dynamics of 5HT’s effects in the ZO.

As we did not observe cleaved caspase-3 immunopositive (CASP3^+^) cells in any cell population in the AM and ZO (excluding exceptionally rare cases), the reduction of chromaffin cells in 5HTP-treated group cannot be mediated via increased apoptosis (Fig. 4e). Thus, the increase of 5HT causes a prolongation of the cell cycle of “bridge” progenitors leading to a decrease in their number, which results in a reduction of derived chromaffin cells (Fig. 4f).

**Fig. 5:**
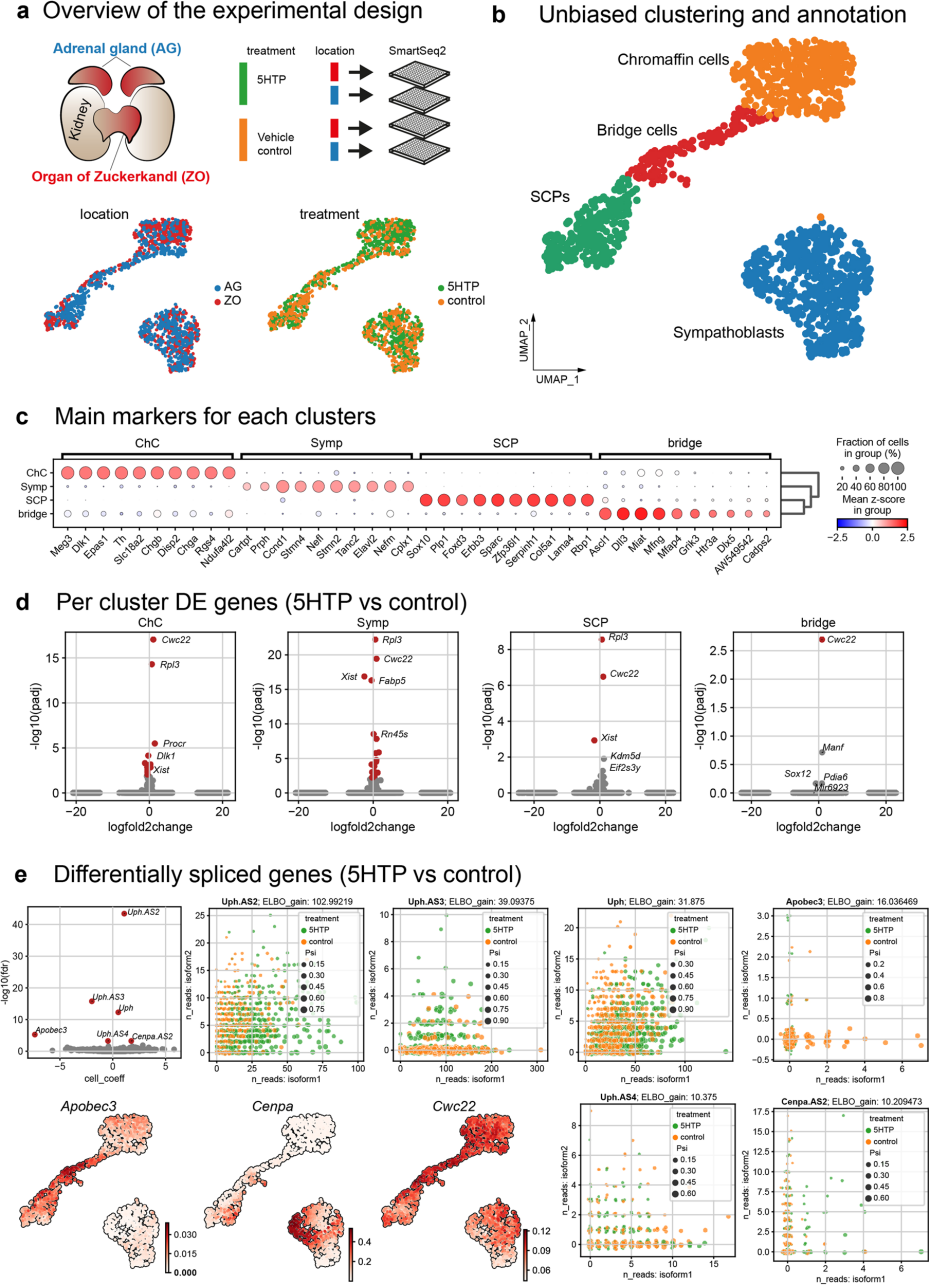
Single-cell transcriptomics reveal differential changes in genes related to RNA splicing and cell cycle upon 5HTP treatment. **a** Adrenal glands (AG) and Organ of Zuckerkandl (ZO) were dissected from E13.5 *Wnt1-Cre*^+/−^;*R26R*^*Tomato*+/wt^ embryos; the tissue was dissociated, and *Wnt1*^Tomato+^ cells were sorted into 384-well plates for Smart-seq2 sequencing. Note the absence of statistically-tested compositional effects (see the “Methods” section) between 5HTP-treated and control conditions, shown as UMAPs (bottom panels). **b** Joint UMAP embedding of cells from AG and ZO from both treated and control groups. **c** Main marker genes defining each cluster. **d** Differential gene expression per cluster in 5HTP-treated vs control groups. **e** Statistically significant differential spliced genes in 5HTP-treated vs control groups. Note that at least four long non-coding RNAs responsible for regulation of *Hand2* activity appeared differentially spliced (*Uph*, *Uph.AS2*, *Uph.AS3*, *Uph.AS4*) and well as *Cenpa*-related long non-coding RNA. The bottom: UMAP plots showing meaningful differentially spliced and differentially expressed genes.

Next, to dissect the potential changes of gene expression upon 5HTP administration, we performed single-cell RNA sequencing of the AM and ZO at E13.5 from 5HTP-treated and untreated embryos (Fig. 5a). We sequenced 1528 cells (both conditions combined) using Smart-seq2 technology, which allows an extraordinary deep sequencing of individual cells (7000/8000 genes per cell on average). The general composition, cell type proportions, and the developmental sequence of cell types (SCPs, “bridge” cells, chromaffin cells, and sympathoblasts) (Fig. 5b, c) remained the same upon 5HTP treatment. At the same time, we detected a reliable change in expression of a gene responsible for the regulation of splicing (*Cwc22*)^38,39^ (Fig. 5d, e). In line with this, we detected changes in differential splicing of several long non-coding RNAs (*Uph*, *Uph.AS2*, *Uph.AS3*, *Uph.AS4*) (Fig. 5e) controlling the expression of *Hand2*^40^, a transcription factor essential for the transition to the catecholaminergic program in chromaffin cells and sympathoblasts^41^. The other differentially spliced genes, *Apobec3* and long non-coding RNA *Cenpa.AS2*^42^, might be involved in the control of a cell cycle length in “bridge” cells, where they are enriched.

### The deficit of 5HT has no effect on chromaffin cells

As the elevated levels of 5HT lead to a decreased chromaffin cell numbers in vivo by delaying the cell cycle of precursor “bridge” cells, we expected to see the opposite effect in the case of reduced levels of 5HT. However, the previously reported genetic loss-of-function of the HTR3A receptor failed to show any abnormal phenotype in adrenal glands^43^. A potential explanation for these observations is that 5HT affects cell cycle progression in HTR3A^+^ “bridge” cells only when 5HT levels reach a certain threshold. Furthermore, HTR3A-dependent paracrine regulation may not be critical for the development of the adrenal gland, but is an important controller of excessive chromaffin tissue growth and pre-malignant states.

To explore how the deficit of 5HT affects chromaffin development, we analyzed several mouse models with a reduction of embryonic and maternal 5HT. *Pet1*^*−/−*^ embryos collected from *Pet1*^*+/−*^ females, as well as *Tph2*^*−/−*^ embryos collected from *Tph2*^*+/−*^ females, lack 5HT derived from the central nervous system of the mutant embryos, although maternal 5HT remains unchanged. The numbers of TH^+^ chromaffin cells did not differ in E15.5 *Pet1*^*−/−*^ and *Tph2*^*−/−*^ embryos in comparison with their *Pet1*^*+/−*^ and *Tph2*^*+/−*^ littermates serving as controls (Supplementary Fig. 8a, b). One of the major peripheral sources of 5HT in the embryo is represented by the enterochromaffin cells, which start to secrete 5HT at E15.5^10^ and therefore cannot contribute to the developing adrenal glands at E12.5–E13.5. Thus, the reduction of embryonic sources of 5HT is not critical for the development of chromaffin cells, while the extraembryonic 5HT might be important.

To address how a complete removal of 5HT from both maternal and embryonic sources affects the development of chromaffin cells in an embryo, we took advantage of the *Tph1*^*−/−*^;*Tph2*^*−/−*^, *Tph1*^*−/−*^;*Scl6a4*^*−/−*^, and *Tph1*^−/−^;*Tph2*^*−/−*^;*Scl6a4*^*−/−*^ mouse models, which lack the ability to produce central and peripheral 5HT and to selectively transport it into cells in both mothers and progeny. We analyzed E13.5 embryos from these mouse models for the number of chromaffin cells and 5HT^+^ cells in adrenal glands. At E13.5, the number of 5HT^+^ cells demonstrated 80% to 95% reduction in all knockout (KO) animals when compared with C57BL/6 controls (Supplementary Fig. 8c, d). The average number of TH^+^ chromaffin cells also appeared reduced in KOs (in *Tph1*^*−/−*^;*Tph2*^*−/−*^ for 11.4%, in *Tph1*^*−/−*^;*Scl6a4*^*−/−*^ for 27.1%, in *Tph1*^*−/−*^;*Tph2*^*−/−*^;*Scl6a4*^*−/−*^ for 31.2%, based on the difference between mean values) (Supplementary Fig. 8d). To check whether the reduction of chromaffin cells was specific, we measured the number of cells in SCG. The number of TH^+^ sympathoblasts in SCG was significantly lower in KO embryos in comparison with wild type embryos (in *Tph1*^*−/−*^;*Tph2*^*−/−*^ for 17.8%, in *Tph1*^*−/−*^;*Scl6a4*^*−/−*^ for 30.2%, in *Tph1*^*−/−*^;*Tph2*^*−/−*^;*Scl6a4*^*−/−*^ for 48.6%, based on the difference between mean values) (Supplementary Fig. 8e), which indicated a general developmental delay and reduction of the embryonic growth, independently of cell origin and due to the lack of maternal 5HT. Therefore, the reduction of chromaffin cells in 5HT deficient models is not specific to the adrenal medulla. Of note, the numbers of SCPs, were not changed in AM and SCG in control and KO embryos (Supplemetary Fig. 8f), because SCPs depend on the local innervation coming from elsewhere. The expression of *Htr3a* mRNA was evident in *Tph1*^*−/−*^;*Tph2*^*−/−*^;*Scl6a4*^*−/−*^ E13.5 adrenal glands, which indicated that “bridge” cells are still present in the KOs (Supplementary Fig. 8g). Overall, these experiments demonstrated that the reduction of maternal and embryonic 5HT has no specific effect on the number of chromaffin cells, contrary to the excess of 5HT during a critical developmental time window.

### High expression of HTR3A in tumorigenic neuroblastoma cells

**Fig. 6:**
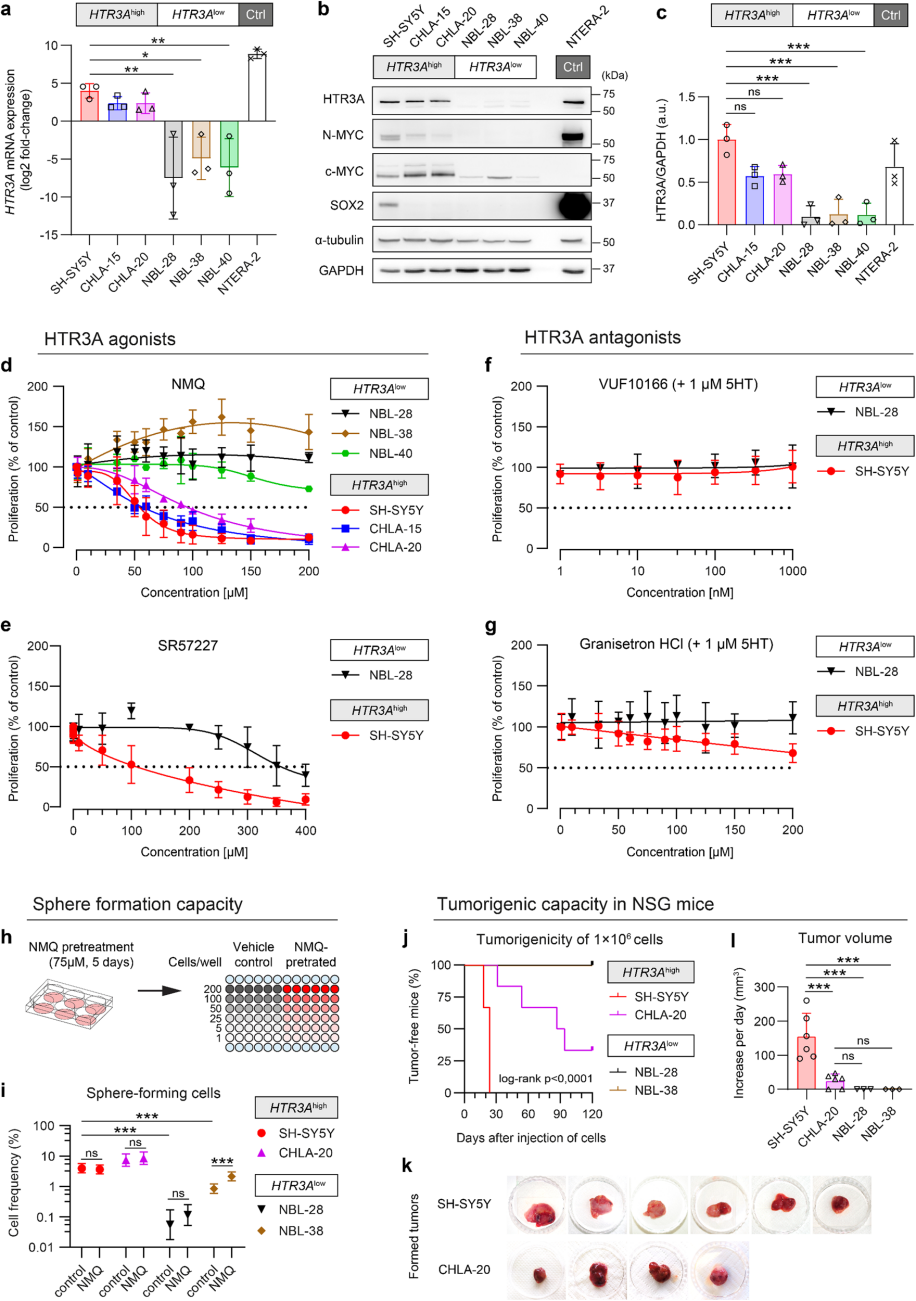
*HTR3A*^high^ neuroblastoma cells are highly tumorigenic and reduce their proliferation under excessive HTR3A stimulation. **a** Gene expression analysis by qRT-PCR revealed marked differences in relative expression of *HTR3A* among individual human neuroblastoma cell lines. NTERA-2 pluripotent embryonal carcinoma cells served as a positive expression control (Ctrl). Data presented as mean ± SD, biological *n* = 3, technical *n* = 3; *p < 0.05, **p < 0.01 using one-way ANOVA followed by Tukey’s post hoc test. **b** Western blotting showed differences in HTR3A protein levels, which also corresponded to the differences in expression of N-MYC, c-MYC, and SOX2 proteins between HTR3A^high^ and HTR3A^low^ groups. Blots are representative of three experiments. **c** Densitometric quantitation of the HTR3A protein expression confirms the defined HTR3A^high^ and HTR3A^low^ groups. Data presented as mean ± SD, biological *n* = 3; ***p < 0.01 using one-way ANOVA followed by Tukey’s post hoc test. **d**–**g** MTT assay on cells treated with agonists of HTR3A receptor (**d**, **e**) revealed significant dose-response decrease in proliferation of *HTR3A*^high^ neuroblastoma cells after 5 days of treatment with either N-methylquipazine dimaleate (NMQ, **d**) or SR57227 (**e**); treatments with HTR3A antagonists (**f**, **g**) did not exert a significant effect on cell proliferation. Data presented as mean ± SD, biological *n* = 3–5, technical *n* = 5. **h** Schematic depiction of limiting dilution sphere formation assay: neuroblastoma cells were pretreated with 75 µM NMQ or vehicle (DMSO) for 5 days and serially diluted in fresh serum-free media w/o the drugs at indicated numbers per well. **i** The frequencies of sphere-forming cells significantly differed between HTR3A^high^ and HTR3A^low^ cell lines, while NMQ pretreatment did not reduced sphere formation capacity of the tested cells. Data are shown as mean ± 95% confidence interval, frequencies, and probability were computed using ELDA software^79^. ***p < 0.01, *χ*^2^ pairwise test. **j**–**l** Only *HTR3A*^high^ neuroblastoma cell lines formed xenograft tumors in NOD/SCID gamma (NSG) mice (**k**). The higher levels of *HTR3A* expression in SH-SY5Y cells corresponded to the earlier onset of tumor formation (**j**) and increased tumor growth (**l**) when compared with CHLA-20 cells; ***p < 0.01 using one-way ANOVA followed by Tukey’s post hoc test.

To investigate the possible action of 5HT on HTR3A in the progression of tumors originating from sympathoadrenal cells, we analyzed several clones of human-derived neuroblastoma for *HTR3A* expression and tumorigenicity using an immunodeficient mouse model. Based on mRNA (Fig. 6a) and protein expression levels (Fig. 6b, c), the examined neuroblastoma cell lines could be characterized as either *HTR3A*^high^ (SH-SY5Y, CHLA-15, and CHLA-20), expressing markedly high levels of *HTR3A*, or *HTR3A*^low^ (NBL-28, NBL-38, and NBL-40), with only weak *HTR3A* expression. While all cell lines were negative for *MYCN* amplification (two copies of gene in the genome), HTR3A protein expression was associated with expression of major drivers of aggressive neuroblastomas, N-MYC and c-MYC^44,45^, or one of the core stemness factors SOX2 (Fig. 6b). Intriguingly, the same association was observed in NTERA-2 embryonal pluripotent carcinoma cells, which served as a positive control for HTR3A expression, and which are known to express high levels of N-MYC and share characteristics with early neural progenitors. To investigate the possible role of HTR3A receptor in regulation of proliferation in tumor cells, we treated the cells with the HTR3A agonists, N-methylquipazine dimaleate (NMQ) and SR57277, as well as antagonists, VUF10166 and granisetron HCl, in the presence of 5HT. HTR3A agonists dramatically limited proliferation of HTR3A^high^ cell lines, whereas they did not affect HTR3A^low^ cell lines, or the effects were seen only at much higher doses (Fig. 6d, e). No cleaved caspase-3 was detected after the treatment with NMQ, indicating that such treatment does not induce apoptosis (Supplementary Fig. 9). In contrast, there was no significant effect of HTR3A antagonists on cell growth of both HTR3A^high^ SH-SY5Y and HTR3A^low^ NBL-28 cell lines (Fig. 6f, g).

We also tested the ability of cells to form spheres to check their stem cell-like properties (Fig. 6h). HTR3A^high^ cells formed spheres more frequently in comparison with HTR3A^low^ cell lines. Importantly, pre-treatment with 75 µM NMQ for 5 days did not reduce sphere-forming capacity of both HTR3A^high^ and HTR3A^low^ cell lines. Therefore, the activation of HTR3A receptor does not compromise the stem-like state of neuroblastoma HTR3A^high^ cells, but only reduces their proliferation. When NMQ is removed the cells form spheres with the same or even increased efficiency (in case of NBL-38), as compared to vehicle-pretreated controls (Fig. 6i).

SH-SY5Y and CHLA-20 *HTR3A*^high^ cell lines formed large tumors in NOD/SCID gamma (NSG) mice, whereas *HTR3A*^low^ cells did not form xenograft tumors even in 4 months after injection (Fig. 6j, k). The tumor volume increase of the xenografts during the experiment (Fig. 6l) further demonstrated the association of *HTR3A* expression with the aggressive phenotype of neuroblastoma cells.

### High 5HT alters catecholamine-mediated behavior in progeny

**Fig. 7:**
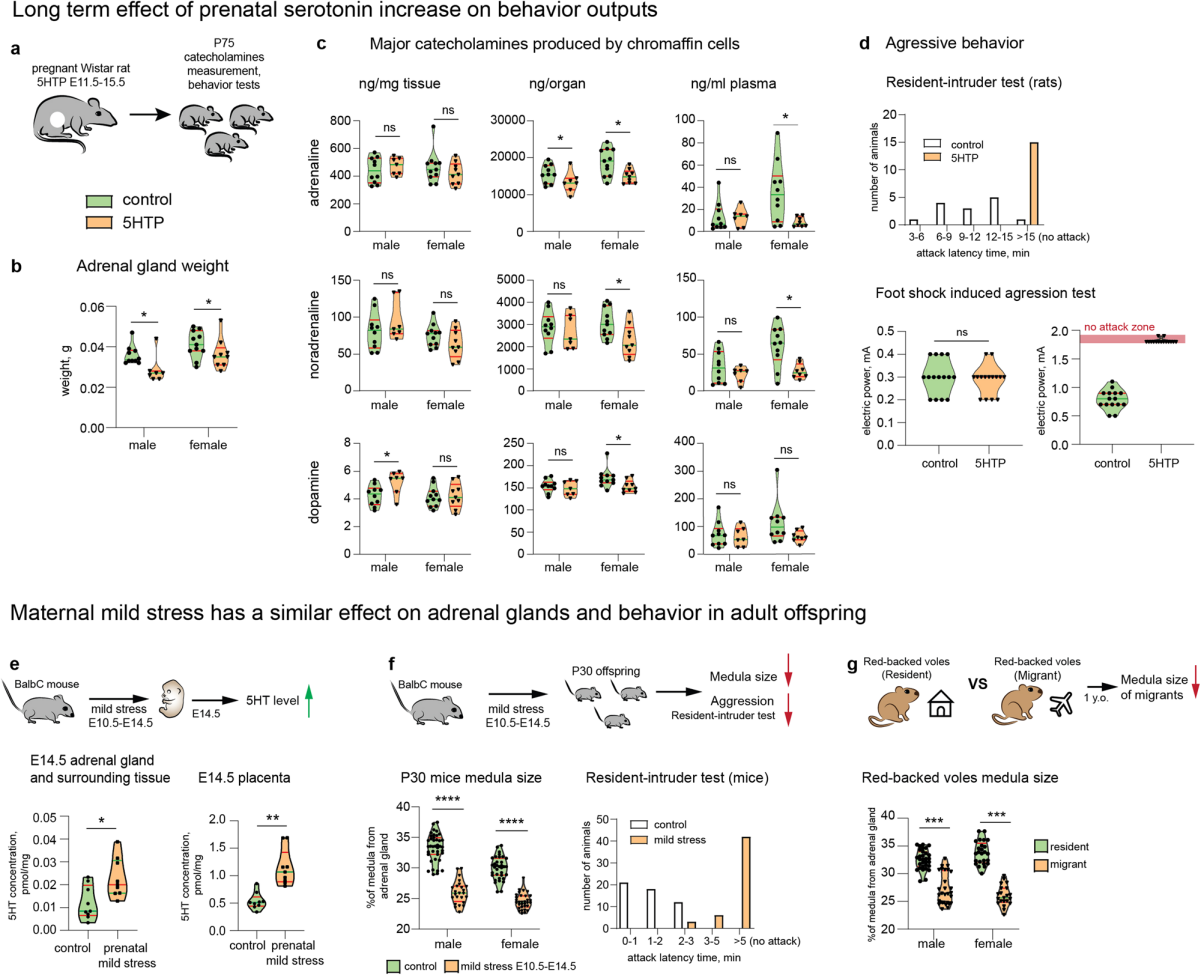
Embryo-to-adult effects of prenatal 5HT on adult behavior and adrenals are similar to the effect of stress induced in pregnant mothers. **a** Pregnant Wistar rats were administered 5HTP during E11.5–E15.5, and their offspring (P)75 was analyzed with the behavioral tests and catecholamine measurements. **b** The size of adrenal glands in animals from the 5HTP-treated females was significantly smaller then from control group. Mann–Whitney test, *p*-value * < 0.05, biological *n* = male: 11 (control), 8 (5HTP); female: 12 (control), 10 (5HTP). **c** Major catecholamines measured in ng/mg of tissue, ng/organ and ng/mL of plasma by HPLC-MS. Note: ng/mg of tissue reflects normal function of chromaffin cells, and does not change; ng/organ decreases in 5HTP-treated group. Mann–Whitney test, *p*-value ns > 0.05, * < 0.05, biological *n* = male: 11 (control), 8 (5HTP); female 12 (control), 10 (5HTP), for plasma *n* = male: 10 (control), 7 (5HTP); female 10 (female), 8 (female). **d** Aggression-assessing behavioral tests: “resident-intruder test” (top) and “foot shock-induced aggression test” (bottom) indicate the reduced aggression in the 5HTP-treated group. Mann–Whitney test, *p*-value ns > 0.05, biological *n* = 14 and 15 (control), 15 (5HTP group). **e** Pregnant BalbC mice were exposed to a mild stress (1 h restrain) at E10.5–E14.5, followed by 5HT measurements in the embryo trunks and placentas. Note: the 5HT levels were increased similarly to experiment with 5HTP-treatment (refer to Fig. 2a, b). Mann–Whitney test, *p*-value * <0.05, ** < 0.002, biological *n* = 9. **f** Pregnant BalbC mice were exposed to a mild stress (1 h restrain) at E10.5–E14.5, and their P30 offspring was tested for the size of adrenal medulla and aggression. Note: the proportion of adrenal medulla was significantly lower in the stress-induced group similarly to the 5HTP-treated group (refer to Fig. 2h). Shapiro–Wilk test for normality, unpaired double-sided *t*-test *p*-value, **** < 0.0001, *n* (adrenal gland) =40 (control male), 24 (1 h restrain male), 35 (control female), 32 (1 h restrain female). **g** The ratio of adrenal medulla of the adrenal gland in *C. rutilus*. Mann–Whitney test, *p*-value * < 0.05, *** < 0.001, biological *n* (adrenal gland) = male: 31 (resident), 29 (migrant); female: 25 (resident), 21 (migrant). In all violon plots the green line—median, the red lines—quartiles.

To evaluate the long-term effect of elevated levels of 5HT during prenatal development in rodents, pregnant Wistar rats were administered 5HTP *per os* during E11.5–E15.5, a stage critical for the transition of “bridge” cells to chromaffin cells. The offspring of treated mothers was maintained until postnatal day 75 (P75), when the behavioral tests and measurements of respective catecholamine levels in blood and adrenal glands were performed (Fig. 7a).

The total body weight of P75 animals was similar in treated and control groups (Supplementary Fig. 10a, b), whereas the weight of adrenal glands was significantly reduced in males and females from the 5HTP-treated group (Fig. 7b). In embryonically-treated adult females, this reduction of adrenals correlated with significantly lower amounts of adrenaline, noradrenaline, and dopamine in the adrenal glands, and lower adrenaline in blood plasma (Fig. 7c). In males from the 5HTP-treated group, smaller adrenal glands contained respectively less adrenaline, but the levels of noradrenaline and dopamine did not show any significant difference (Fig. 7c). To ensure that the observed reduction of adrenaline in males and the reduction of adrenaline, noradrenaline, and dopamine in females were not associated with accelerated catabolism by catechol-O-methyltransferase in blood, we measured catecholamine metabolites: metanephrine and normetanephrine (Supplementary Fig. 10c). These measurements confirmed that the decrease in adrenaline supply from the adrenal glands in the 5HTP-treated group was not due to the acceleration of adrenaline catabolism (Supplementary Fig. 10c). On the other hand, the concentration of normetanephrine was significantly higher in 5HTP-treated males, suggesting an enhanced catabolism of noradrenaline that is typically observed in major pheochromocytoma subtypes in human^46,47^. The elevation of normetanephrine is not observed in females. Of note, high metanephrine levels might not always reflect high catecholamine synthesis and following catabolism, as inhibition of monoamine oxidase may also lead to elevated levels of catecholamines/metanephrines, which we cannot rule out even though we consider this scenario unlikely based on the previous literature^48–51^. The system of catecholamines and their metabolism is complex and stretches beyond the production of adrenaline and noradrenaline in chromaffin organs. Therefore, the observed differences in males and females, as well as the only partial correlations with behavioral data might be due to other systemic regulatory mechanisms, which are not covered by this study.

In the next line of behavioral experiments, we assessed how the prenatal exposure to enhanced 5HT during the critical time window of chromaffin cell generation shaped the adaptive capacity of the offspring. According to the resident-intruder test and foot shock-induced aggression test, the males from 5HTP-treated mothers were less aggressive and did not defend their territory, compared with control males (Fig. 7d). Moreover, the experimental animals were more adaptive and flexible, demonstrating less anxiety and reduced stress-induced behavior (Supplementary Fig. 10d). These results are in line with in vivo measurements of catecholamines in adult mice revealing lower levels of adrenaline supplied by smaller adrenal glands (evident when measured as ng/organ, Fig. 7c) in animals prenatally exposed to elevated 5HT levels.

### Stress affects fetal 5HT, medulla size, and progeny behavior

The connection between embryonic 5HT levels and subsequent animal behavior allowed us to hypothesize a connection between the stress of a pregnant female, 5HT and the development of adrenals in progeny.

The following line of experiments based on the published method^52^ revealed that mild stress induced by 1-h-restrain of pregnant mice (E10.5–E14.5) significantly enhanced 5HT level in both placenta and fetuses (Fig. 7e, f), causing decreased medulla size in a progeny (Fig. 7e, f), similarly to 5HTP exposure (Fig. 2b, h). The resulting experimental progeny demonstrated less aggressive behavior according to the standard resident-intruder test: they showed cooperative behavior and reduced number of attacks on the intruder (Fig. 7f). Overall, the prenatally 5HTP-treated animals and the progenies from stress-induced mothers showed consistency in adrenal medulla reduction and behavioral changes. Although our data from mice and rats appear to be consistent, we do not claim that the effects of maternal stress on postnatal behavior are only rooted in decreased adrenals and embryonic influence from the mother. However, mild stress and 5HTP treatment during the same embryonic period result in similar behavioral outcomes in postnatal progeny, and are associated with a decreased AM size. Overall, the prenatal effects and postnatal effect are likely connected and correlate via the reduced number of chromaffin cells in adrenal glands.

### The ratio of medulla to cortex changes in migrating voles

As the experimentation on laboratory animals might not fully reflect the ecological and evolutionary situation, we committed to perform the analysis of adrenals in wild rodents with different well-documented intra-species modes of behavior. For this, we set out for an expedition to Yenisey Ecological Station “Mirnoe” (62.2 N; 89.0 W) in Siberia, to study the wild population of red-backed voles (*Clethrionomys rutilus*). *C. rutilus* represent a “cycling” population with periods of expansion and migration, with well-defined residents and migrants triggered by the spiking increase of density^53–55^. To check if there is a correlation between the size of adrenal medulla and resident vs migrant profile in wild *C. rutilus*, we measured the medulla size in representatives of residents and migrants in the year of a peak population density accompanied by enhanced migration activity (2020). The results showed that the migrant animals of both sexes are characterized by reduced medulla size as compared to residents (Fig. 7g), which might be connected to the fact that the increase in population density is associated with increased chronic stress^56^. According to our experiments with laboratory animals and previously published results^52^, increased level of stress in pregnant mothers elevates 5HT in the placenta and embryo. This, in turn, results in reduced chromaffin organs in the offspring, altered hormonal profiles and more cooperative and flexible catecholamine-controlled behavior.

## Discussion

Recent findings^6,8^ have challenged the older paradigm about the immediate origin of chromaffin cells from ventrally migrating neural crest cells^57^, and raised questions about how the numbers of chromaffin cells are established and controlled during embryonic and postnatal life. These findings introduced intermediate cell states intercalating into the trajectory from the neural crest to chromaffin cells. One state is represented by nerve-associated Schwann cell precursors^6,58^ giving rise to “bridge” cells, which in turn transit into chromaffin cells^6^.

Here we investigated how these intermediate cell types regulate their numbers and define the future size of the chromaffin population. We found that 32% of the chromaffin cells are 5HT positive as soon as they emerge at E12.5 in mouse and this number goes up to 77.8% at E14.5. The 5HT, which is derived from chromaffin cells and a small population of “bridge” cells, signals to neighboring HTR3A^+^ “bridge” cells, lengthening their cell cycle. This causes a reduction of adrenal medulla because less “bridge” cells become available for differentiation into non-dividing chromaffin cells. Being 5HT-sensitive, “bridge” cells are a part of a negative feedback loop controlling the size of the 5HT-releasing chromaffin population. Similar modes of paracrine feedback loops were shown for retinoic acid signaling^59^ or angiogenic growth factors^60^ which are known to control organogenesis. Overall, the paracrine and autocrine role of 5HT in developing chromaffin organs results in two important aspects related to health and survival: protection from chromaffin tissue overgrowth or neoplasia, and prevention of excessive catecholamines and catecholamine-controlled behavior. In humans, the sparse expression of *HTR3A* appeared in “bridge” population at weeks 5-to-7 according to RNAseq and at weeks 6 and 8 according to experimental validations with RNAscope. Despite the expression was detected in principle, it appeared low and at the border of detection, leaving a question about the role of *HTR3A* in human “bridge” cells open. Contrary to this, the expression of *HTR3A* in sympathoblasts showed a strong and consistent pattern, also in agreement with other studies^22^. Therefore, the human-specific role of the described paracrine regulation via 5HT and chromaffin progenitor-specific HTR3A is probable, although it requires further experimentation to be validated. Of note, avian chromaffin cells^29^ and the cells of the oxygen sensory organ (carotid body) are 5HT-positive as well^61^, and may employ the 5HT-dependent mechanism to control their numbers.

The single-cell transcriptomics analysis showed that elevated embryonic 5HT influences the expression of *Cwc22*—a key gene controlling splicing^38,39^, and also changes the levels of differentially spliced proliferation—and differentiation-related genes expressed by a “bridge” population (*Apobec3*, long non-coding RNAs controlling *Hand2* and potentially *Cenpa*).

The proposed mechanism of chromaffin cell number control via 5HT-HTR3A-dependent paracrine regulation is unidirectional, as the complete elimination of 5HT and pharmacological blockage of HTR3A receptor do not result in overgrowth of chromaffin cell organs. This goes in line with previous reports showing that the excess of 5HT has stronger effects on brain development as compared to the lack of 5HT^62,63^. For example, *Sert*^*−/−*^ mice demonstrate elevated levels of extracellular 5HT in the brain, which causes a number of structural abnormalities related to the role of 5HT during brain development together with depressive- and anxiety-like behavior^64^, with no changes in adrenal gland morphology and catecholamine release^65^. Conceptually similar results were obtained via inhibition of SERT with selective 5HT reuptake inhibitors at E8–E18 of mouse development^62,66^. On the other hand, *Pet1* KO and *Tph2* KO mice lack 5HT, but have structurally normal brains (although *Pet1* KO mice lack serotonergic neurons) with altered anxiety-related behavior^67,68^. These results are in line with our loss-of-function experiments, where the drastic decrease of embryonic and maternal 5HT did not yield any alteration of size of chromaffin organs, apart from the general reduction of an embryo size reported previously^11,12^. Thus, the 5HT-dependent control of chromaffin cell numbers protects only against the excessive growth and, potentially, tumor-permissive situations.

Consistent with this reasoning, chromaffin organs within the sympathoadrenal complex represent the sites of childhood tumor neuroblastoma, as well as pheochromocytoma and paraganglioma. The comparison of human *HTR3A*^high^ and *HTR3A*^low^ neuroblastoma cell lines revealed that cell lines with *HTR3A*^high^ expression level have higher tumor-initiating potential. Those cell lines had key characteristics of cancer stem cells and appeared tumorigenic in a mouse xenograft model system as well as formed significantly more spheres in vitro. In line with the in vivo cell cycle progression experiments, we managed to inhibit the proliferation rate of *HTR3A*^high^ neuroblastoma cells with a specific HTR3A agonist, which might be developed into a potential therapeutic strategy, especially in a combination with differentiation-inducing drugs^69–71^. Still, it might be wise to keep in mind the potential difference between tumor and healthy HTR3A^+^ cells, as the tumor cells might have additional, unpredictable effects following from HTR3A activation, and the relevance of 5HT paracrine regulation within tumors remains to be elucidated. Moreover, the origin of neuroblastoma is highly debatable^9,22,72–75^, and our results regarding the feedback loop mechanism involving 5HT and HTR3A in  “bridge” cells in vivo and in cancer cell lines should be interpreted with great care.

Hypothetically, beyond the anticancer-related roles, the 5HT-dependent chromaffin tissue control might have important behavioral, ecological and evolutionary dimensions. Indeed, in addition to local chromaffin cells synthesizing 5HT from 5HTP directly within chromaffin organs, the other major source of embryonic 5HT are represented by the maternal blood^76^ and placenta^14,77^. The biosynthetic enzymes TPH1 and DDC are produced in the syncytiotrophoblastic cell layer of the murine placenta, which is in line with previous observations of in vitro placental 5HT neo-synthesis at E10.5–E18.5 in mice. In line with this observation, human placental fetal villi demonstrated a similar biosynthetic capacity during early gestation^77^. The combination of different 5HT sources and the switch from systemic (extraembryonic) to the local source of 5HT were previously noticed during embryonic brain development in mice^14^. Similar to brain development, the presence of extraembryonic-derived 5HT in the embryonic circulation adds another variable to the equation of chromaffin cell number control. In fact, not only local paracrine/autocrine regulation might take place in developing chromaffin organs, but also systemic extraembryonic 5HT might influence the future size of the adrenal medulla. In turn, the intensity of 5HT synthesis in placenta depends on the availability of mother-derived biochemical precursor 5HTP and tryptophan. This opens a potential for a non-genetic control of adrenergic organ development in a progeny of mothers capable of tuning the levels of tryptophan and 5HTP. This tuning might depend on stress factors or health conditions^52,78,79^. Interestingly, chronic mild stress, excessive maternal inflammation, and hypoxia lead to the increased 5HT synthesis in the fetal placenta with increased output to the fetus, as was previously shown for rodents and humans^80^. In our experiments, prenatal mild stress in pregnant females resulted in elevated levels of 5HT in progeny, which reduced adrenal medulla and changed the offspring behavior similarly to the experiment with chemically (5HTP)-treated mothers. In this “chemical” in vivo experiment, we mimicked the maternal-dependent elevation of 5HT by introducing higher levels of 5HTP into pregnant females. As a result, we detected a reduced number of chromaffin cells, smaller adrenal medulla and decreased levels of catecholamines associated with changes in the behavior of the adult offspring.

One can admit that the molecular mechanisms controlling the size of chromaffin tissues are important for natural and artificial selection. Although we do not provide direct data supporting this idea, the low aggressiveness, changes in 5HT synthesis and degradation, and reduction of chromaffin organs were previously reported as a part of the so-called “domestication syndrome”, observed in a number of domesticated species^20,21^. In line with these domestication-associated behavioral patterns, our experimental rodents subjected to 5HT-driven reduction of adrenals showed less aggressive responses and altered levels of catecholamines. The individual levels of aggressive behavior are indeed related to how the animals react to a wide variety of environmental challenges including two major coping strategies—proactive and reactive^81^. Aggressive males typically express a more proactive type of behavioral response demonstrating rigid, cue-independent, and impulsive reactions and a tendency to defend their home territory. At the same time, non-aggressive reactive males are rather flexible, cautious, and open to the external cues, which can assist in variable or unpredictable environments, such as during migration^81,82^. Our results demonstrated that prenatal exposure to enhanced 5HT during the critical time window (resulting from maternal mild stress or availability of 5HTP) leads to the birth of progeny with a preferentially reactive type of a coping strategy. This suggests a possible non-genetic information transmission from mother to progeny via the 5HT-dependent developmental control of chromaffin organ size. Conceptually, a similar 5HT-based mother-to-progeny information transmission was identified in invertebrates^13,83,84^ and, given our results, might be a more general strategy in nature^85^.

Furthermore, the mechanism of 5HT-based mother-to-progeny information transmission might be more evident in a wild population under critical environmental stress. The oscillating population density in red-backed voles (*C. rutilus*) periodically reaches exceptional values and causes unprecedented social stress to individual animals^56^. This, along with other factors, forces the voles to segregate into residents and migrants, shaping the population cycles^86^. The difference between the animals forced to migrate and those who stay might involve a behavioral control of aggression, which is affected by the different size of chromaffin organs. We tested this hypothesis and found an association of the adrenal medulla size with the type of the coping strategy (resident—proactive and migrants—reactive) in wild rodents. This association supports the connection between stress in pregnant mothers, 5HT concentrations and the resulting size of chromaffin organs in progeny. Overall, the described mechanism of chromaffin cell number control via 5HT-sensitive precursor cells may provide a regulatory 5HT-mediated pathway of prenatal programming for long-lasting changes in progeny underlying the behavior of domesticated species as well as wild animals with active and reactive types of coping strategy. Future research should establish the role of genetic factors responsible for the variation of the chromaffin organs in wild animals as compared to the power of the 5HT-based mother-to-progeny information transfer mechanisms.

## Methods

### Animals and corresponding ethics

All experiments involving laboratory animals were done in accordance with European Convention on the Protection of Vertebrate Animals Used for Experimental and Other Scientific Purposes (Strasburg, 1986) and approved by the Ethics Committee for Animal Research of the Koltsov Institute of Developmental Biology (Russian Academy of Sciences, approval code: 22, approved on 15 March 2018) as well as in accordance with The Swedish Animal Agency’s Provisions and Guidelines for Animal Experimentation recommendations and approved by Ethical Committee on Animal Experiments (Norra Djurförsöksetiska Nämd, ethical permit N226/15).

Laboratory animals were kept in standardized conditions (24 ˚C, 12:12 h light–dark cycle, 40–60% humidity, food, and water ad libitum).

*Htr3a*^*EGFP*^ transgenic animals are Tg(Htr3a-EGFP)DH30Gsat/Mmnc) were received from MMRRC and provided by the J. Hjerling-Leffler laboratory (Karolinska Institutet, Stockholm, Sweden) (https://www.mmrrc.org/catalog/sds.php?mmrrc_id=273). Animals were kept as heterozygotes for the EGFP transgene. *Wnt1-Cre* (The Jackson Laboratory, stock number 009107), full strain name B6.Cg-Tg(Wnt1-cre)11Rth Tg(Wnt1-GAL4)11Rth/J) and reporter mice line *R26R*^*Tomato*^ (The Jackson Laboratory stock number 007914) were used for the study. As wild type animals Wistar Rat, BalbC, and C57Bl6 mice were used.

For all experiments, a single embryo was considered as a biological *n*, and the embryos from 1 to 2 litters were used in experiment to comply with the 3R policy about the usage of animals in research. Furthermore, the exact timing of the embryonic development varies depending on the time of conception and embryo implantation, which can be up to 12 h apart. The developmental difference within this time-window can affect the results and introduce the unwanted noise into the assessment of developing organs at E8–E14.5 stages. Based on our previous experience with such variation, pulling several litters into one comparison can result in much greater variability within the overall group, which can hide the true differences. Selecting and aligning the perfectly comparable litters requires the unnecessary sacrifice of higher numbers of without much of necessity. With this in mind, and with the goal to minimize the number of animals used, we focused on the analysis of adequate numbers of individual embryos (3–6) serving as biological replicates for our studies. For the majority of the experiments, we utilized 1–2 litters per experimental condition.

NOD/ShiLtSz-*scid/Il2rγ*^null^ mice were used as a NSG model. All NSG mouse experiments were conducted in accordance with a study (21379/2011-30) approved by the Institutional Animal Care and Use Committee of Masaryk University and registered by the Ministry of Agriculture of the Czech Republic as required by national legislation.

E15.5 embryos (gender was not identified) from *Pet1*^*−/−*^ knockout mice were received from Patricia Gaspar (INSERM: Paris, France). E15.5 embryos (gender was not identified) from *Tph2* knockout mice were received from Massimo Pasqualetti from (Dept of Biology, University of Pisa, Italy). E13.5 embryos (gender was not identified) from *Tph1*;*Tph2*, *Tph1*;*Scl6a4,* and *Tph1*;*Tph2*;*Scl6a4* knockout mice together with E13.5 embryos (gender was not identified) of C57BL/6 mice bred in the same facility were received from Natalia Alenina and Michael Bader (Max-Delbruck Center for Molecular Medicine (MDC), Berlin, Deutschland). Before shipment embryos were fixed in 4% paraformaldehyde in PBS (pH 7.4) at 4 °C 4–6 h depending on the embryonic stage. Samples were rinsed in PBS and placed in 30% sucrose in PBS for cryoprotection. Samples were sent in 30% sucrose in PBS solution incubated on ice and processed for immunohistochemical analysis after receipt.

All experiments involving wild animals red-backed voles *Clethrionomys rutilus* were approved by the Biomedical Ethics Commission of FSBI “Zakusov Institute of Pharmacology” (Russian Academy of Sciences, approval code: 01, dated 20 January 2017). Red-backed voles *C. rutilus* (Pallas, 1779) is not an endangered species. The wild representatives of red-backed voles *C. rutilus* were collected during August-September 2020 in the Yenisey ecological station “Mirnoe” (62.2 N; 89.0 W), Turukhansky region of Krasnoyarsk territory, within the Central Siberia Biosphere Reserve. The long-term population-ecological investigations demonstrated a 4-year cycle of population dynamics of Micromammalia which is stable in the conditions of Yenisey Central Siberia. *C.rutilus* population dynamic peak occurred in Central Siberia in 2020 (mean 29,1 animals per 100 traps per day in 2020, compared to mean 13,8 animals per 100 traps per day in 2018. Year monitoring of ecological station “Mirnoe”), and the collection period at the late summer\autumn considered the most relevant to estimate the wave dynamics in cycling population^55^. Representatives of *C. rutilus* collected in Sherman traps (live traps) in taiga (dark coniferous forest consisting of Siberian cedar, fir, pine, and larch) were considered as settled individuals or residents. Representatives collected in pitfall traps in Yenisey River valley (meadows without tree communities) were considered as migrants. Trapped species were settled individually in plastic boxes for 2 h. The animals were weighed and adrenals were dissected under inhalant isoflurane anesthesia (SomnoSiute system, Kent Scientific, USA). The gender, pregnancy and fertility status, approximate ages were determined visually after dissection. Altogether 24 females and 31 males of ~1 year old were used for the study.

### 5HTP administration to pregnant females and embryo collection

Three- to four-month-old females of Wistar rats, BalbC mice, and 2–4-month-old *Htr3a*^*EGFP*^ mice were placed in time-controlled mating and the day of plug was considered as embryonic day (E) 0.5 for mice, day of conception was considered as embryonic day (E) 0.5 for rats. 5-hydroxy-L-tryptophan (Sigma-Aldrich H9772) was dissolved in saline and fed (1 mg/kg BW, once a day) to pregnant rats through gavage or intraperitoneally injected (40 mg/kg BW, once a day) to pregnant mice females. At the stage of embryo harvest, the females were sacrificed by cervical dislocation after anesthesia with 2–3% isoflurane, embryos were eviscerated and placed in ice-cold PBS. The gender of embryos was not identified.

### HTR3A agonist and antagonist treatment

Three- to four-month-old pregnant BalbC females received intraperitoneal (i.p.) injections of the selective agonist of the HTR3A receptor, SR57227 hydrochloride (2 mg/kg BW, once a day) at E11.5 and E12.5 stages. Another group of animals was i.p. administered with the HTR3A antagonist, granisetron hydrochloride (2 mg/kg BW, once a day) in combination with 5HTP (40 mg/kg BW, once a day) at E11.5 and E12.5 stages. Drugs were purchased from Tocris (UK), dissolved in DMSO, and then diluted in sterile physiological saline. In the control group, mice received identical volumes of isotonic saline with DMSO (DMSO control). E13.5 embryos were fixed in 4% paraformaldehyde and processed for immunohistochemistry. The gender of embryos was not identified.

### MicroCT tissue preparation and analysis

E15.5 rat embryos (gender was not identified) were fixed in 4% paraformaldehyde in PBS (pH 7.4) at 4 °C for 6 h. Samples were dehydrated by incubation in the ethanol gradient solutions in PBS (30%, 50%, 70%); incubation was done at 4 °C with agitation for 24 h in each concentration. For contrasting the samples were transferred to 1.0% PTA (Phosphotungstic acid, Sigma-Aldrich, P4006) in 90% methanol and incubated with rotation at 4 ˚C with agitation for 3 weeks; the PTA solution was changed weekly. After contrasting, the samples were rehydrated through a methanol gradient (90%, 75%, 50% and 30%). After that, rehydrated embryos were shipped on ice to Brno University of Technology, Czech Republic for microCT scanning.

Samples were placed in polypropylene tubes and embedded in 1% agarose gel to minimize movement during microCT measurement. MicroCT scanning was performed using system GE phoenix v|tome|x L 240, equipped with a 180 kV/15 W maximum power nanofocus X-ray tube and high flat panel dynamic 41|100 with 4000 × 4000 pixels and a pixel size of 100 × 100 μm. The exposure time was 900 ms in 2000 positions over 360°. The microCT scan was carried out at 60 kV acceleration voltage and with 200 μA X-ray tube current. The beam was filtered by a 0.2 mm-thick aluminum filter. The voxel size of obtained volumes was 2.2 μm for all samples. The tomographic reconstructions were performed using GE phoenix datos|x 2.0 3D computed tomography software. Segmentation of structures was performed manually by a combination of software Avizo (Thermo Fisher Scientific, USA) and VG Studio MAX 3.2 (Volume Graphics GmbH, Germany).

### Thymidine analogues labeling during S-phase combined with 5HTP treatment

Double thymidine analogues labeling of cells in S-phase was based on the methods described previously^87^. 5-ethynyl-2′-deoxyuridine (EdU, Life Technologies, E10415) and 5-Chloro-2′-deoxyuridine (CldU, Sigma-Aldrich, C6891) were dissolved in PBS at stock concentrations 5 mg/ml and 5.2 mg/mL and, respectively, intraperitoneally injected to 2–4-month-old *Htr3a*^*EGFP*^ mice pregnant females in equimolar concentrations to 50 mg/kg body weight of EdU with 4-h interval. Females also received 2 injections of 5HTP 40 mg/kg or vehicle control at E11.5 and at E12.5. To minimize the number of 5HTP injections, at E12.5 and the EdU dose were combined in one injection solution. Embryos were harvested 4 h after CldU injection E12.5, gender of the embryos cannot be identified.

### Tissue preparation for immunohistochemistry

Whole embryos, dissected sympathoadrenal complexes were fixed in 4% paraformaldehyde in PBS at 4 ˚C with agitation for 2–6 h followed by rinse in PBS. After fixation samples were cryoprotected in 30% sucrose in PBS at +4 °C with agitation for 24 h. Samples were embedded in OCT and frozen at −20 °C. 14 µm serial sections were produced from each sample, collected on SuperFrost microscopy slides (Thermo Scientific) and kept at −20 °C before staining.

### Immunohistochemistry

Cryosections were brought to room temperature and dried for at least 2 h before antigen retrieval. Antigen retrieval was done by submerging the slides in 1× Target Retrieval Solution (Dako, S1699) in water, bringing the solution to boiling and letting it cool down for 40–60 min. Sections were washed three times for 10 min in PBS containing 0.1% Tween‐20 (PBST), encircled by Super PAP Pen (Invitrogen, 008899), and incubated at room temperature overnight with primary antibodies diluted in PBST in a humidified chamber. Then, sections were washed in PBST three times for 10 min and incubated with secondary antibodies and DAPI (5 µg/mL) diluted in PBST at RT for 90 min, washed again three times in PBST and mounted using Mowiol (Merck, 81381) mounting medium, prepared according to manufacturer’s instructions.

### Detection of thymidine analogues in combinations with IHC

Antigen retrieval was done by submerging the slides in 1× Target Retrieval Solution (Dako, S1699) in water, bringing the solution to boiling point, and cooling it down for 40–60 min. Sections were washed three times for 10 min in PBS. Sections were further permeabilized with 4% triton X-100 solution for 1 h followed by 3 washes 10 min in PBS.

EdU visualization was performed by click reactions. Sections were incubated in Click Buffer 1 (0.1 M Tris pH 7.5, 20 µL CuSO_4_ 100 mM, 5 µM Alexa Fluor 647 azide (ThermoFisher, A10277) (stock 10 mM in DMSO), 100 mM ascorbic acid) for 15 min with gentle rocking. Washed three times for 5 min in PBS and incubated with Click Buffer 2 (20 µL CuSO_4_ 100 mM, 40 mM ascorbic acid and 2 mM azidomethyl phenyl sulfide (Sigma-Aldrich) in PBS) for blocking of non-specific reactions of anti-BrdU (clone BU1/75) antibody with EdU^68^. The sections were incubated for 15 min with gentle rocking followed by 3 washes for 5 min in PBS. For CldU visualization with antibodies, a DNA denaturing step by 2N Hydrochloric acid (incubation at 37 °C for 40 min) is critical to allow the antibody to bind to DNA. The sections were neutralized by washing in 0.1 M borate buffer (pH 8.0) two times for 10 min followed by three washes in PBS for 5 min. Then sections were blocked in 5% normal donkey serum (Sigma-Aldrich), 0.1% Triton X-100 in PBS for 1 h. Primary antibodies were dissolved in 5% normal donkey serum (Sigma-Aldrich), 0.1% Triton X-100 in PBS and incubated with sections overnight at RT with gentle rocking. A combination of anti-BrdU (recognizes CldU) antibody and cell type-specific antibodies was applied. Next day, the sections were washed in PBS three times for 5 min and incubated with the solution of secondary antibodies and DAPI in PBS for 90 min at RT. After incubation, the samples were washed in PBS three times for 10 min at room temperature and mounted using Mowiol mounting medium, prepared according to manufacturer’s instructions.

### Primary and secondary antibodies

The following primary antibodies were used: rabbit anti-TH (1:1000, Pel-Freez Biologicals, #P40101-150), sheep anti-TH (1:2000, Novus Biologicals, #NB300-110), rabbit anti-serotonin (1:2000, ImmunoStar, #20080), chicken anti-GFP (1:600, Aves Labs Inc., #GFP-1020), goat anti-human SOX10 (1:800, R&D Systems, #AF2864), rabbit anti-KI67 (1:500, Thermo Scientific, #RM-9106), rabbit anti-Cleaved Caspase-3 (1:500, Cell signaling, Asp175), rat anti-BrdU (1:300, Abcam, BU1/75, also recognizes CldU).

For detection of the primary antibodies, secondary antibodies raised in donkey and conjugated with Alexa-405, -488, -555, and -647 fluorophores were used (1:1000, Molecular Probes, ThermoFisher Scientific). Goat anti-chicken conjugated to Alexa fluor-488 (1:600, Jackson ImmunoResearch, 703-545-155).

### RNA scope® in situ hybridization

Fluorescent in situ hybridization manual assay against *Pnmt* (probe 426421-C3) and *Htr3a* (probe 411141-C3) was performed using the RNAscope Fluorescent® Multiplex Assay kit according to manufacturer’s instructions (Advanced Cell Diagnostics). Immunostaining following the hybridization was performed as described above except for the antigen retrieval step.

### Microscopy

Images were acquired using LSM700, LSM 710, and LSM 880 confocal microscopes (Carl Zeiss, Germany) equipped with ×10, ×20, and ×40 objectives. Images were acquired in the .lsm format.

### Cell counts and area measurements

Cell counts and area measurements were done manually using the Cell counter plugin and measurement functions of ImageJ (2.1.0/1.53c) software. The area of adrenal gland section was calculated by surrounding the area based on DAPI signal. The area of medulla was calculated based on TH^+^ signal within adrenal gland. The area of cortex was calculated by subtraction of adrenal medulla area from the area of the whole gland per individual section. Three section per gland and 2 glands per embryo were evaluated.

Venn diagrams were built with https://www.meta-chart.com/venn#/your-charts free on-line platform and redrawn in Adobe Illustrator 25.2.1. The percentage of Sox10^+^*Htr3a*^EGFP+^ cells was calculated as a fraction of Sox10^+^*Htr3a*^EGFP+^ cells from the sum of all Sox10^+^ and all *Htr3a*^EGFP+^ at E13.5 and E14.5 in adrenal medulla.

### Measurements of relative medulla size in postnatal animals

The dissected adrenals of P30 BalbC mice and collected red-backed voles *Clethrionomys rutilus* were processed for whole-mount Benzyl alcohol/Benzyl benzoate tissue optical clearing method. The preparations were scanned using LSM 880 confocal microscope (Carl Zeiss, Germany) with a green channel determining the tissue autofluorescence. The optical section with maximal external volume was selected for relative medulla size analysis. The area of central medulla and the whole adrenal were measured using ImageJ software. Relative medulla size represented by the ratio: (medulla area/total area) × 100%.

### Re-analysis of single-cell transcriptomic data published by Furlan et al., 2017

We re-analyzed single-cell RNA-seq data of mouse adrenal gland from Furlan et al.^6^. Gene counts were obtained from GEO database (GSE99933). Gene count matrix was analyzed with a standard Seurat (version 3.0.2) workflow^88^. We used the original embeddings and clustering from ref. ^6^ (Figs. 5B and 5G), downloaded from the published pagoda apps: http://pklab.med.harvard.edu/cgi-bin/R/rook/nc.SS2_16_249-2/pathcl.json and http://pklab.med.harvard.edu/cgi-bin/R/rook/nc.SS2_16_250-2/pathcl.json (json slots embedding/data for the embedding and colcols/clusters/data for the cluster labels).

Seurat function FeaturePlot and DotPlot were used to plot gene expression in individual cells on the embedding and average gene expression in the clusters as dot plots, respectively.

### Single-cell RNA sequencing of mouse tissue by SmartSeq2

*Wnt1-Cre*^+/−^;*R26R*^*Tomato*+/wt^ E13.5 embryos prenatally treated with 5HTP or vehicle for control at E11.5 and E12.5 were harvested in ice-cold PBS. Adrenal glands and Organ of Zuckerkandl were dissected under the stereoscope equipped with a fluorescent light source. Tissue was added to 500 µL of 0.05% Trypsin/0.02% EDTA (Sigma, 59417-C) and incubated at 37 °C for 10 min. The tissue was triturated slowly with a P-200 pipette until complete dissociation. Trypsin was deactivated by adding 500 µL of PBS containing 10% FBS. The cells were centrifuged at 500 × *g* at 4 °C for 5 min. The cells were washed two times with PBS + 10% FBS followed by centrifugation. The non-single cell clusters of cells were removed by filtering through 40 μm-pore size cell strainers. Fluorescent cells were sorted in single cells mode into 384-well plates prefilled with lysis buffer according to a previously published protocol using a BD FACS Aria III Cell Sorter. One full 384-well plate was sorted and sequenced per organ and per treatment. Single-cell library preparation was done following Smartseq2 protocol^89^.

### Single-cell transcriptomics data analysis of SmartSeq2

First, generation of count matrices, QC and filtering was performed. The single-cell transcriptome data were generated at the Eukaryotic Single-cell Genomics facility at Science for Life Laboratory in Stockholm, Sweden. The samples were analyzed by first demultiplexing the .fastq files using deindexer (https://github.com/ws6/deindexer) using the NextEra index adapters and the 384-well plate layout. Individual .fastq files were then mapped to mm10_ERCC genome (https://www.ncbi.nlm.nih.gov/assembly/GCF_000001635.20/) using the STAR aligner version 2.7.5c using 2-pass alignment. Reads were filtered for only uniquely mapped and were saved in BAM file format; count matrices were subsequently produced. Estimated count matrices were gathered and converted to an anndata object. Cells with the following quantities were kept as high quality: having more than 10^5^ and less than 5 × 10^5^ transcripts, containing more than 4000 and less than 8000 detected genes and or less than 15% of ERCC reads. Cells with low number of reads, potential doublets, cells with high fraction of ribosomal and mitochondrial genes were removed from the analysis. The resulting filtered count matrix contained 1361 high-quality cells (out of 1528 total cells sequenced).

The preprocessing and initial analysis of the count matrix was performed without any batch correction, using scanpy python package v1.7.2, scFates python package v0.2.4, and scvelo python package v0.2.3. Highly variable genes were detected using pagoda2 approach (scFates.pp.find_overdispersed, default parameters). Cell cycle genes were removed from the list of high variable genes to remove their effect. PCA was performed on the scaled count matrix using the high variable genes (scanpy.pp.pca, default parameters). kNN graph (scanpy.pp.neighbors, n_neighbors = 30, n_pcs = 30, metric = “cosine”) was generated from PCA space, followed by UMAP embedding generation (scanpy.tl.umap, default parameters) and leiden clustering (scanpy.tl.leiden, Resolution = 0.3)^90^. Differential gene expression analysis was performed per cluster between treated cells and control, using Wilcoxon rank-sum test.

To detect possible differentially spliced genes between the two conditions, BRIE2 algorithm was employed^91,92^. Counting of the isoform-specific reads in each splicing event was performed for BAM files of the QC filtered cells, using filtered mouse annotation provided by the package maintainers. The annotation has the following characteristics: from GENCODE mouse vM17, exon skipping events located on autosome and chrX, not overlapped by any other AS-exon, surrounding introns are no shorter than a fixed length 100 bp. Length of alternative exon regions, between 50 and 450 bp. With a minimum distance, 500 bp, from TSS or TTS. Specific splice sites: surrounded by AG-GT, i.e., AG-AS.exon-GT. Splicing isoform proportion and variable splicing detection was performed in mode 3, by including a design matrix containing the both treatment and location as columns, location effect was removed as potential cofounder.

### Ethical aspects of procedures involving human tissue

Human pre-natal tissue was obtained from clinical routine abortions with oral and written consent from the patient. Swedish Ethical Research Authority and the National Board of Health and Welfare has approved the acquisition of human pre-natal tissue for research purposes (ethical reference is 2018/769-31 with the addendum EPN2019-04595). The material was donated for a general research purpose especially with the focus on neuronal and nervous system-related cell types. Measurement of crown-rump-length (CRL) and anatomical landmarks were used to determine the post-conception age.

### Human fetal cell isolation, storage in methanol, and rehydration

The tissue was received in ice-cold PBS and enzymatically digested to obtain the single-cell suspension. For this, tissue cut into smaller pieces was incubated with 500 μl 0.05% Trypsin/0.02% EDTA (Sigma, 59417-C) for 15 min at 37 °C with gentle swirling every 5 min. Gentle pipetting up and down was used to mechanically dissociate bigger pieces if any. 500 μl 10% FBS in PBS was added to cell suspension and the cells were pelleted at 500 × *g* for 5 min at 4 °C. Cells were washed two times with 1000 μL PBS, passed though the 35 μm cell strainer (Falcon, 352235), pelleted at 500 × *g* for 5 min at 4 °C and re-suspended in 100 μl 0.04% BSA in PBS. Ice-cold methanol (400 μL was added for fixation of the cells and the cells were stored at −80 °C. For the preparation of the library the cells were brought to +4 °C and pelleted at 1000 × *g* for 10 min at 4 °C. Cell pellet was re-suspended in 500 μL of rehydration buffer (1X DPBS (Gibco 14190144) containing 1.0% BSA (Sigma, B4287) and 0.5 U/μl RNAse Out (ThermoFisher Scientific, 10777019) followed by two washes with 500 μL of rehydration buffer. The rehydrated cell suspension was sorted to remove debris with BD FACS Aria Fusion instrument (BD Biosciences, San Jose, CA) equipped with 100 μm nozzle. After FACS cells were pelleted to obtain concentrated cell suspension with 700–1200 cells/μL.

### 10x Genomics RNAseq library preparation and sequencing of human cells

10x Genomics Chromium Single Cell 3ʹ protocol for Reagent Kits v3 was used for library preparation aiming to recover 5000–8000 cells. Sequencing was done on Illumina NovaSeq 6000 Sequencing System (NovaSeq 6000 S1 Reagent Kit or NovaSeq 6000 S2 Reagent Kit were used) with the standard recommended read set up for 10X Genomics libraries: Read 1: 28 cycles (Cell barcode and UMI), i7 index: 8 cycles (Sample index), Read 2: 91 cycles. The 10X single-cell transcriptome data were generated at the Eukaryotic Single-cell Genomics facility at Science for Life Laboratory in Stockholm, Sweden.

### Human single-cell transcriptomics data analysis with 10x Genomics

The count matrix for each sample was produced by Cell Ranger version 3.1.0 that processed, mapped, and counted raw sequencing data to the Cell Ranger GRCh38-3.0.0 genome and its corresponding annotation. Seurat package pipeline (v.4.0.2)^93^ was used for the downstream analysis. Genes expressed in fewer than ten cells in each dataset were removed from further analysis. To keep only high-quality cells, the cells with less than 2000 or more than 25,000 transcripts and the cells with less than 1500 detected genes per cell were omitted. The cells with high mitochondrial content (more than 10%) were also excluded from the analysis. The putative doublets were predicted by Scrublet^94^. The filtered datasets first were analyzed separately to extract the cells belonging to the neural crest-derived sympathoadrenal lineage and then integrated with Seurat function IntegrateData (2000 integration anchor features, 20 dims). The resulting integrated filtered dataset consisted of 3503 high-quality cells. The effects of cell cycle heterogeneity in gene expression were mitigated by regressing out the difference between G2M and S phase signatures by Seurat functions CellCycleScoring and ScaleData (vars.to.regress = “CC.Difference”). Then to perform a graph-based clustering and visualization by UMAP, the first 30 principal components and 30 nearest neighbors were used. Louvain clustering algorithm with resolution equal to 0.2 resulted into finding 13 clusters, two of them were removed due to containing the high doublet scores. The remaining clusters were re-analyzed using the same parameters except the resolution (30 PCs, 30 kNN, resolution = 0.1) and the resulting seven clusters were merged based on the expression of classical cell type-specific markers into four biologically meaningful clusters. To check whether the expression of *HTR3A* gene in the “bridge” cells is not noise-derived, the exact Fisher test was applied after removal of the sympathoblast clusters from the dataset.

### Behavioral studies

The tests were performed on 21 male and 25 females P75 Wistar rats, weighing 180–200 g. Animals were acclimatized 15 min a day for 5 days before the tests^95^.

Resident-intruder test was performed on rat and mice^81^. For the test on Wistar rats, each resident male was housed with the companion female in the resident cage (floor space of about half a square meter) for 1 week prior to testing. The companion females were sterilized 3 weeks before the test. The cages were not cleaned until the end of the experiment. Testing was performed once a day at 8:00 p.m. The companion female was removed from the cage and an unfamiliar male was introduced in the residential cage, followed by recording the behavior of the resident for 15 min. The time between the introductions of the intruder and the first clinch attack was considered as attack latency.

For the test on BalbC mice, male mice were singly housed for 1 week prior to testing. The cages were not cleaned until the end of the experiment. The male intruders were group-housed (five per cage) and matched with resident mice for approximate age and body weight. The unfamiliar male was introduced in the residential cage and behavior of resident mice was monitored during 5-min after exposure to male intruder. One trial per day was conducted at 8:00 p.m. The time between the introductions of the intruder and the first biting attack was considered as attack latency.

Foot-shock induced aggression test^96^ was performed on rats. Two male rats were placed on the electrode floor of the test chamber for both, pain sensitivity and aggression, and then the current was gradually increased at a rate of 0.1 mA/s. The threshold of pain sensitivity was determined by the minimum current at which the animals made the first squealing. The minimum value of the current that causes the typical upright defensive postures (threat posture) in a rat was considered as the threshold of aggressiveness. The test was stopped at 1.8–1.9 mA/s.

Elevated Plus-maze (EPM) test was performed on rats. The apparatus for EPM test was constructed from 2 horizontal arms 50 cm long and 10 cm wide crossing each other in the middle at 90° angle. Two opposing parts of the maze have 40 cm high walls “closed arms”. The maze was elevated 40 cm from the floor. The rat was placed in the middle of the apparatus facing one of the closed arms. The time the rat spent on the central part of the maze, in open and closed arms, and the number of entries to open and closed arms were recorded. The total time of one animal observation was 5 min^97^.

Novelty-induced hypophagia test was performed on rats. The apparatus for Novelty-induced hypophagia test is a cylindrical platform 97 cm in diameter, with a 42 cm high wall made from white plastic. The floor of the platform has the marks of the central circle, 23 cm in diameter and the middle part, 18.5 cm wide, divided into 6 equal size sectors and the peripheral part 18.5 cm wide, divided into 12 equal size sectors. The source of light was 100 W lamp 1 m above the floor of the platform. In the center of the platform, the food was placed. During 10 min of the test the duration of food take latency was measured.

Extrapolation Escape Task was performed on rats. In this task animals need to find an escape solution from an acute stress situation. The apparatus consists of a basket with an internal cylinder 25 cm high and 10 cm in diameter. The basket is filled with 21–23 °C water up to 2 cm from the bottom of the internal cylinder. Rats were placed in the internal cylinder and their behavior was observed during 2 min. The dive latency and time to find the escape ladder was registered.

### Catecholamine analysis

The catecholamine standards (norepinephrine (NE), epinephrine (E), and dopamine (DA)) and catecholamine metabolites (normetanephrine (NM), metanephrine (M)) were obtained from Sigma-Aldrich (USA). The catecholamine and catecholamine metabolites labeled internal standards (norepinephrine-d6, epinephrine-d3 and dopamine-d4, normetanephrine-d3, metanephrine-d3) were obtained from TRC (Canada). All reagents were of analytical grade. Methanol was obtained from Thermo Fisher Scientific (Fisher Scientific UK Ltd.). Ethyl ester was obtained from Panreac (Germany). 2-Aminoethyl diphenylborinate, Formic acid, Hydrochloric acid, Sodium hydroxide, Ammonium chloride were obtained from Sigma-Aldrich (USA). Water used in the entire analysis was prepared using Milli-Q water purification system from Millipore (UK).

A Shimadzu HPLC system (Japan) was used for chromatographic separation of analytes on an Eclipse XDB-C18 (150 mm, 4.6 mm, 5 mm) analytical column, maintained at 40 °C in a column oven. Gradient elution was used for the chromatographic separation of analytes. The mobile phase A: 0.1% formic acid in water, the mobile phase B: 0.1% formic acid in methanol.

A triple quadrupole mass spectrometer Shimadzu 8060 (Japan), equipped with electrospray ionization and operating in positive ionization mode was used for detection of analytes and ISs. For quantitation, multiple reaction monitoring (MRM) was used to monitor precursor-product ion transitions *m*/*z* 151.90 → 77.10 (norepinephrine), *m*/*z* 158.90 → 111.00 (norepinephrine-d6), *m*/*z* 183.90 → 107.00 (epinephrine), *m*/*z* 187.00 → 107.00 (epinephrine-d3), *m*/*z* 165.90 → 121.15 (normetanephrine), *m*/*z* 169.00 → 137.00 (normetanephrine-d3), *m*/*z* 153.90 → 91.10 (dopamine), *m*/*z* 157.00 → 94.00 (dopamine-d4), *m*/*z* 179.90 → 165.15 (metanephrine), *m*/*z* 183.00 → 168.00 (metanephrine-d3).

#### Calibration standards and quality control samples

Stock solutions of analytes (1 mg/mL) were prepared by dissolving accurately weighed reference standards in 0.1% HCl in water. Stock solutions of ISs (1 mg/mL) were prepared by dissolving accurately weighed reference standards in 0.1% formic acid in water. Calibration standards (CSs) and quality control (QC) samples were prepared by spiking blank sample (water) (10% of total volume of blank sample) with mixed stock solutions. CSs were made at concentration levels (Table 1).

**Table 1:** Composition of calibration standards.

№	Name	NM/D/E/M/NE concentration, ng/ml
1	Cal 1	1/50/5/1/5
2	Cal 2	10/100/25/10/25
3	Cal 3	50/250/50/50/50
4	Cal 4	100/500/100/100/100
5	Cal 5	500/750/250/500/250
6	Cal 6	1000/1000/500/1000/500

QC samples were prepared at two concentration levels (Table 2).

**Table 2:** Composition of quality control samples.

№	Name	NM/D/E/M/NE concentration, ng/ml
1	QСL	3/75/15/3/15
2	QСH	800/800/400/800/400

All the solutions (standard stock, CSs and QC samples) were stored at −20°C until use.

To extract catecholamines, adrenal glands were weighed, homogenized in 0.9% NaCl, and frozen in liquid nitrogen. Heparinized blood samples were collected from the heart and centrifuged. Samples were kept at −80 °C until the mass spectrometric analysis of catecholamines and their metabolites.

Prior to analysis, all frozen samples, CSs and QC samples were thawed in RT. To each glass tube (16 × 100 mm) 0.5 ml CSs, QC or sample; 20 µL of the internal standards mix and 0.8 mL of buffer containing a complexing agent (0.2% DPBA-ethanolamine ester and 5 g/L EDTA in 2 mol/L NH_4_ Cl–NH_4_ OH buffer, pH 8.5) was added. After mixing, the pH of the complexed sample preparation was checked with a pH probe or narrow range pH paper. If the pH was <7.5, it was adjusted with concentrated ammonia to be between 7.5 and 9.5. Ethyl Ester (1500 μL) was added to extract the analytes and vortex-mixed (10 min), and the sample was then centrifuged for 10 min at 3000 × *g*. The Ethyl Ester layer was removed (800 μL) and placed into a recovery vial (Waters Corp., Elstree, UK). The vial solution was then evaporated to dryness using a centrifugal vacuum evaporator (Eppendorf, USA). The samples were reconstituted in 200 μL (0.1% formic acid in water), and 2 μL was injected onto the column.

Quantification was performed using Shimadzu LabSolution software, version 5.3 (Japan) using the integration peak area ratio of analyte and labeled IS.

### 5HT measurement by high-performance liquid chromatography with electrochemical detection (HPLC-ED)

5HT concentration was determined after the following experiments: (1) Pregnant BalbC mice were subjected to mild stress based on a published method^52^ for 1 h daily from E10.5 to E13.5. 5HT was measured at E14.5 in placenta and fetal tissues; (2) Pregnant BalbC mice received 5HTP from E10.5 to E13.5. 5HT was measured at E14.5 in placenta and fetal tissues; (3) Pregnant BalbC mice received 5HTP from E10.5 to E13.5. At E 14.5 fetal adrenals and kidneys were dissected and incubated for 1 h in DMEM medium at 37 °C in a humidified 5% CO_2_/95% air atmosphere. About 16–20 adrenals and 10–12 kidneys were pooled per sample. After the incubation 5HT was measured in culture medium and fetal tissues.

A Shimadzu HPLC-ED LC-20ADsp (Japan) was used for chromatographic separation of analytes on a reversed-phase ReproSil-Pur column, ODS-3, 4 × 100 mm with pore diameter of 3 µm (Dr. Majsch GMBH, Germany) at a temperature of 30 °C and a flow rate of 1.0 mL/min. The mobile phase consisted of 0.1 M citrate–phosphate buffer containing 0.3 mM sodium octanesulfonate, 0.1 mM EDTA and 7% acetonitrile (all reagents purchased from Sigma) (pH 2.56). Decade II electrochemical detector (Antec Leyden, Netherlands) was equipped with a glassy carbon working electrode (+0.85 V) and an Ag/AgCl reference electrode. Collected tissues were homogenized in 10 volumes of 0.1 N HClO_4_ containing 3,4-dihydroxybenzylamine hydrobromide (internal standard, Sigma, St. Louis, USA) (25–250 pmol/mL) by an ultrasonic homogenizer (Sonopuls HD 2000.2, Bandelin, Berlin, Germany), centrifuged at 16,500 × *g* for 20 min, and 5HT in the supernatant were measured. Peaks corresponding to 5HT were identified according to elution time of the standard solution, and the contents of substances were estimated as a ratio of the peak area of the internal standard solution to that of the specimen using software LabSolutions (Shimadzu, Japan).

### Cell cultures

Patient-derived neuroblastoma cell lines NBL-28, NBL-38, and NBL-40 were established from tumor tissue samples^98,99^, with written informed consent obtained for our previous research project (IGA MZCR NR/9125-4), approved by the Research Ethics Committee of the School of Medicine, Masaryk University, Brno, Czech Republic (Approval No. 23/2005). According to Czech legal and ethical regulations governing the use of human biological material for research purposes, a new ethical assessment of this study is not necessary. In brief, the tumor biopsy was disinfected, cut into ~2 mm pieces, and placed into T25 flask with 1 mL of complete medium based on DMEM (PAA Laboratories, Linz, Austria) supplemented with 20% fetal calf serum (PAA), 2 mM glutamine and 1× penicillin/streptomycin (BioWhittaker, Inc., Walkersville, MD, USA) under 37 °C and 5% CO_2_. After specimen attachment, the volume of the medium was slowly brought up to 5 ml during 48 h. Once outgrowing cells reached 60% confluency, they were passaged and maintained. The SH-SY5Y neuroblastoma cell line was purchased from ECACC cat. # 94030304). CHLA-15 and CHLA-20 neuroblastoma cell lines were obtained from Alex’s Lemonade Stand Foundation Childhood Cancer Repository (cccells.org) and kindly provided by Dr. Michael D. Hogarty (Children’s Hospital of Philadelphia, PA, USA). Pluripotent embryonal carcinoma cell line NTERA-2 (clone D1) was purchased from ECACC (cat. # 01071221) and served as a control of HTR3A expression. Cells were cultured in DMEM/Ham’s F-12 medium mixture (1:1; all neuroblastoma cells) or DMEM (NTERA-2) supplemented with 10% (CHLA-15, CHLA-20, and NTERA-2) or 20% fetal calf serum (FCS), 2 mM l-glutamine, 100 IU/mL penicillin, and 100 μg/mL streptomycin (all from Biosera, Nuaillé, France), at 37 °C with 5% CO_2_. For all neuroblastoma cell lines, media were further supplemented with 1% of nonessential amino acids (Biosera) and in case of CHLA-15 and CHLA-20 also with 1× ITS-X (Gibco). All cell lines were routinely authenticated by STR profiling.

### qRT-PCR

Total RNA was extracted with the GenElute™ Mammalian Total RNA Miniprep kit including genomic DNA elimination step using the On-Column DNase I Digestion Set (both Sigma-Aldrich, St. Louis, MO, USA). For all samples, equal amounts of RNA (25 ng of RNA/1 μL of total reaction content) were reverse transcribed into cDNA using M-MLV (Top-Bio, Prague, Czech Republic) and oligo-dT (Qiagen Inc., Valencia, CA, USA) priming. Quantitative PCR was performed in 10 µL reaction volumes using the KAPA SYBR® FAST qPCR Kit (Kapa Biosystems, Wilmington, MA, USA) and 7500 Fast Real-Time PCR System and 7500 Software v.2.0.6 (both Life Technologies, Carlsbad, CA, USA). The expression of individual genes was assessed using at least three technical replicates from three biological replicates of each cell line. The heat shock protein gene HSP90AB1 was used as the endogenous reference control. Following primers (5′→3′) were used for this study: *HTR3A* (5-hydroxytryptamine receptor 3A) forward—AGGAAGCCAACCACCGTATC; *HTR3A* reverse—GTCCGTGGGGATGGACAACT; *HSP90AB1* (Heat shock protein 90 alpha family class B member 1) forward—CGCATGAAGGAGACACAGAA; *HSP90AB1* reverse—TCCCATCAAATTCCTTGAGC.

### Western blotting

Whole-cell extracts were collected using RIPA buffer and 30 µg of total proteins were resolved on 10% polyacrylamide gels and blotted onto PVDF membranes (Bio-Rad Laboratories, Munich, Germany). The membranes were blocked with 5% non-fat dry milk in Tris-buffered saline with 0.05% Tween-20 (Sigma) and incubated overnight with primary antibodies. The following antibodies were used: rabbit anti-HTR3A (1:5000, Abcam, #ab13897), rabbit anti-c-MYC (1:1000, Cell Signaling Technology, CST, #5605), rabbit anti-N-MYC (1:1000, CST, #84406), rabbit anti-SOX2 (1:1000, CST, #3579), rabbit anti-cleaved caspase-3 (1:1000; CST, #9664), rabbit anti-GADPH (1:10,000, CST, #2118), and mouse anti-α-tubulin (1:10,000, Abcam, #ab7291). The next day, the membranes were incubated for 1 h with HRP-linked secondary antibodies: goat anti-rabbit IgG (1:5000, CST, #7074) or horse anti-mouse IgG (1:5000, CST, #7076). Chemiluminescent detection was performed using Amersham ECL Prime (Cytiva, Marlborough, MA, USA) and either Azure 600 imaging system (Azure Biosystems, Dublin, CA, USA) or photosensitive film. GAPDH or α-tubulin served as loading controls. Protein band densities were quantified using ImageJ (Fiji) software (NIH, Bethesda, MD, USA), version 2.1.0/1.53c.

### Neuroblastoma xenografts in NSG mice

Eight-week-old female NSG (NOD/ShiLtSz-*scid/Il2rγ*^null^) mice were injected subcutaneously into the right flank with a suspension of 1 × 10^6^ enzymatically dissociated cells in 100 μL of serum-free DMEM. The mice were examined every three days over the period of 4 months for the presence of subcutaneous tumors. After the development of a tumor or after 4 months, the mice were sacrificed and surgically examined. The xenograft tumors were excised and photographed, and the final tumor volume was measured using the following formula: tumor volume (mm^3^) = length (mm) × width (mm) × width (mm) × 1/2.

### MTT cell proliferation assay

Neuroblastoma cells were seeded in 96-well plates at a density of 10^3^ cells/well in a defined serum-free medium: DMEM/F12 based medium (as detailed in Cell lines) w/o FCS, supplemented with 10 ng/mL EGF (Sigma-Aldrich), 20 ng/mL FGF2 (Sigma-Aldrich), and 1× B-27 supplement w/o vitamin A (Gibco). After 24 h, cells were treated by the addition of fresh medium with the selective HTR3A receptor agonists, N-methylquipazine dimaleate (NMQ; Tocris, cat. #0566) or SR57227 (Tocris, cat. #1205), or the HTR3A receptor antagonists, granisetron hydrochloride (Tocris, cat. #2903) and VUF 10166 (Tocris, cat. #10166). In case of HTR3A receptor antagonists, medium was further supplemented with 5HT (Merck, cat. #14927) to the final concentration of 1 µM to evaluate the effect of HTR3A receptor inhibition. The proliferation activity was analyzed after additional 5 days using 3-[4,5-dimethylthiazol-2-yl]-2,5-diphenyltetrazolium bromide (MTT) (Sigma) at a final concentration of 455 μg/ml^100^. The medium with MTT was replaced by 200 μL of DMSO per well after a 3 h incubation under standard conditions in order to solubilize the MTT product. The absorbance was measured at 570 nm with a reference absorbance at 620 nm wavelength using a Sunrise Absorbance Reader (Tecan).

### Limiting dilution sphere formation assay

Prior to sphere formation assay, cells were treated for 5 days with 75 µM NMQ or an equivalent concentration of vehicle (DMSO). Cells were then harvested and dissociated into single-cell suspension by Accutase (Biosera), re-suspended in a defined serum-free medium (detailed above) and serially diluted into ultra-low attachment 96-well plates (Corning, cat. #3474) to reach final cell densities of 200, 100, 50, 25, 5, and 1 per well. At least four technical replicates of each cell density were included per cell line and treatment group. Every three days, growth factors were replenished by addition of fresh defined medium. After a week, for each cell density, the fraction of wells containing neurospheres ≥50 µm in diameter was determined using an Olympus CKX41 light microscope with Lumenera Infinity 2 CCD camera and QuickPhoto Camera 2.3 system (PROMICRA, Prague, Czech Republic). Frequencies of sphere-forming cells among experimental groups were calculated and compared using ELDA software^101^.

### Statistical analysis

Statistical analysis was done using GraphPad Prism 8.1.1 software. All datasets were checked for normality with Shapiro–Wilk test, as this test reliably works for small datasets. For datasets with normal distribution double-sided unpaired t-test was applied. For datasets which failed a test for normal distribution, Mann–Whitney test was applied. For comparisons between multiple groups for one variable the one-way ANOVA test was applied (Fig. 3d, Fig. 6a, c, l, Supplementary Fig. 7d, e, f). In case one-way ANOVA test was showing significant differences, Tukey’s multiple comparison test was used for a robust pairwise comparison of groups with unequal size (every mean to every other mean) or Dunnet’s multiple comparison test for a pairwise comparison of groups to a control group.

In the analyses of statistical significance in catecholamine measurement as well as in behavioral tests (Fig. 7, Supplementary Fig.8) we have chosen the pairwise comparison within genders. The reason for it is that the treatment with 5HTP or 1-h restrain was applied at the stage, when the sexual dimorphism is not established in the forming adrenal glands and, consequently, the treatment equally affects males and females. However, adrenal glands have sexual dimorphism at postnatal stages and therefore must be analyzed separately, since the combined analysis of these groups can mask the effects of our prenatal treatment. We do not report a quantitative difference in catecholamines and catecholamine metabolites concentration. We have calculated statistical significance with Mann–Whitney test within genders between treated and control animals as this test is applicable to small samples and can evaluate statistical significance regardless of the normality of data distribution and homoscedasticity of the data. For these experiments the analysis was performed in SigmaPlot 12.1.

One of the values of catecholamine content in plasma in female 5HTP-treated group was removed based on the τ-criterion (blunder detection techniques). The outlier value from one animal may be caused by an error when taking a blood sample. Indeed, catecholamine release from adrenals is known to be very sensitive to various factors (in particular, stressful ones, at the time of sampling).

For all violin plots, median and quartiles are shown as those values work equally good for normally and not normally distributed data points.

### Reporting summary

Further information on research design is available in the Nature Research Reporting Summary linked to this article.

## Supplementary information

Supplementary Information

Reporting Summary

Peer review

## Data Availability

The raw and processed data of single-cell transcriptomic experiments generated in this study have been deposited in the GEO database under accession codes: GSE180861 (mouse), GSE195929 (human). The single-cell RNA-seq data of mouse adrenal gland from Furlan et al. (2017) used in this study are available in the GEO database under accession code GSE99933, mm10_ERCC genome used in this study is available in the RefSeq database under accession code GCF_000001635.20. The data other than RNA-seq data generated in this study are provided in the Source data file.

## Source data

Source Data

## Code availability

The code for reproducing the bioinformatic analysis can be found on the following GitHub repository: [https://github.com/LouisFaure/Adrenal5HTP_paper] (10.5281/zenodo.6247197).
