# Supplementary material for: Serotonin limits generation of chromaffin cells during adrenal organ development
Source: Nat Commun. 2022 May 25;13:2901. doi: 10.1038/s41467-022-30438-w (PMC9133002; doi:10.1038/s41467-022-30438-w)
Supplement: Supplementary file 1 — Supplementary Information [file 41467_2022_30438_MOESM1_ESM.pdf]

Supplementary Figure 1

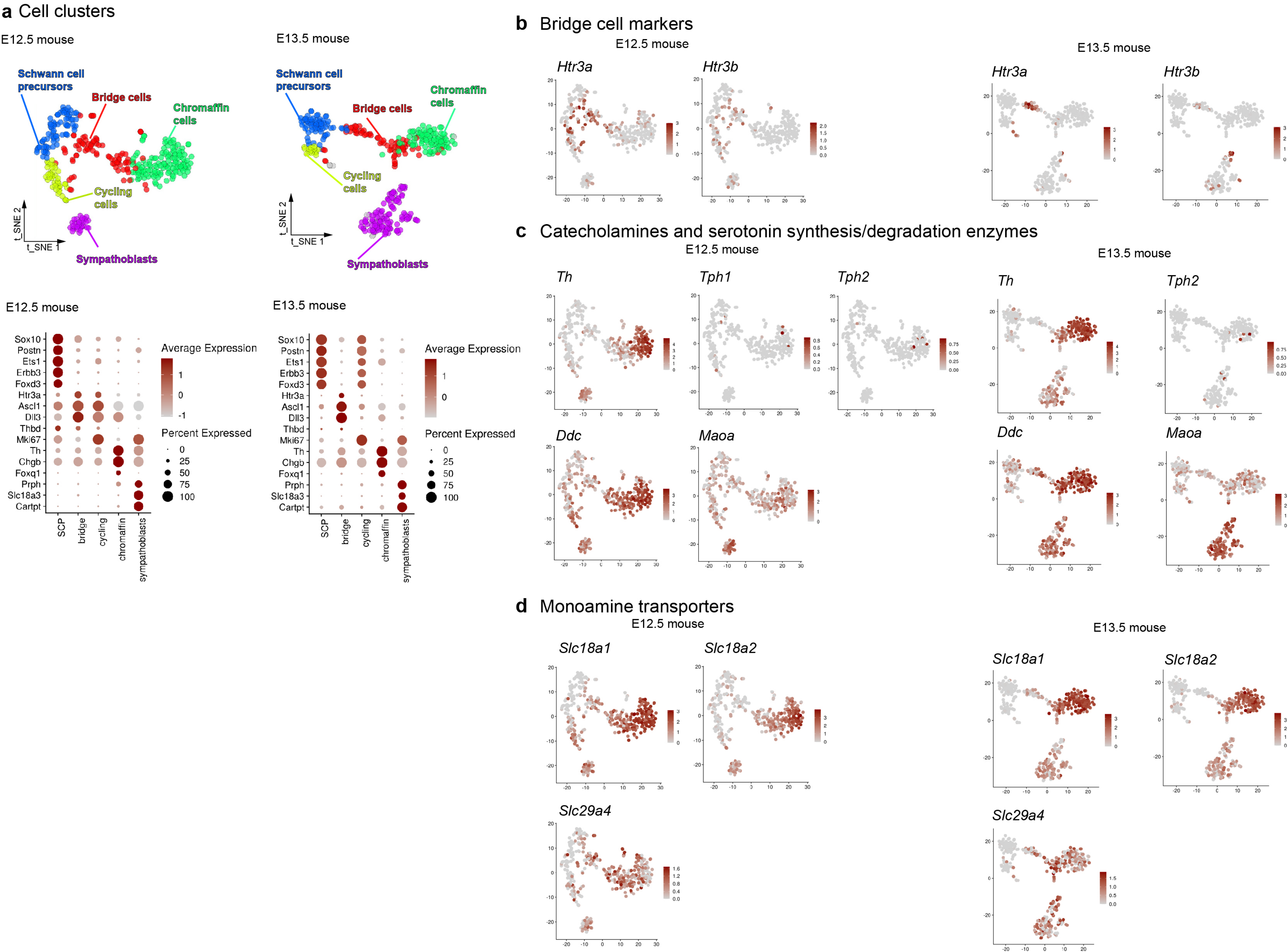

Supplementary Fig. 1 Gene expression associated with 5HT receptors, synthesis, degradation and transporters in the murine sympathoadrenal lineage at E12.5 and E13.5.

a, t-SNE embedding of single cell RNA profiles (top panels) and dot plot for major genes, defining clusters (bottom panels) in the murine sympathoadrenal anlagen at E12.5 and E13.5 previously published by Furlan et. al.[1] Clusters of cells are grouped and annotated based on the transcription programs. Note: a cell cluster is shown to connect Schwann cell precursors with chromaffin cells, named “bridge” cells. **b**, Expression of genes encoding for *Htr3a, Htr3b* is sporadically expressed in SCPs at E12.5 and specifically in the “bridge” cell cluster and in a minor portion of sympathoblasts. At E13.5 the expression of *Htr3a, Htr3b* is limited to the “bridge” cell cluster and a minor portion of sympathoblasts. **c**, Expression of genes encoding for catecholamine, serotonin synthesis and monoamine degradation in all cell clusters. **d**, Expression of genes encoding for monoamine transporters in all cell clusters.

Supplementary Figure 2

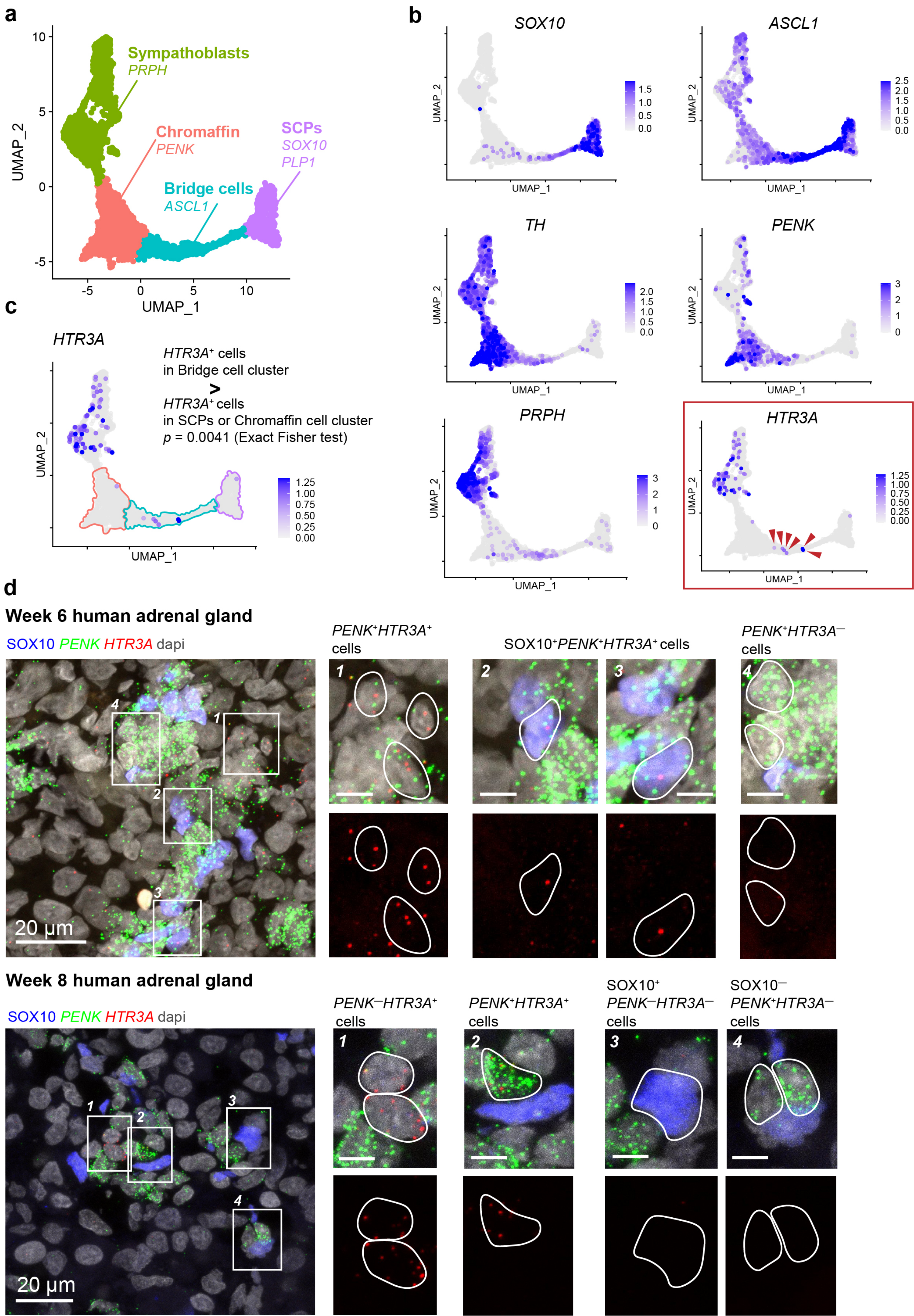

Supplementary Fig. 2 Expression of *HTR3A* during human adrenal gland medulla development.

a, UMAP embedding of single cell RNA profiles of cells from adrenal gland medulla primordium at 5-7 postconceptional weeks (pcw) with annotated cell clusters. SCPs - Schwann cell precursors. **b**, Expression of markers of the clusters and *HTR3A*. *HTR3A*$^{+}$ cells are shown by arrows in “bridge” cells cluster. **c**, Exact fisher test on the number of *HTR3A*$^{+}$ cells in “bridge” cell cluster in comparison with SCPs and Chromaffin cells. **d**, Combined immumohistochemistry against SOX10 (marker of SCPs) and RNAscope *in situ* hybridization for *PENK* (marker of chromaffin cells) and *HTR3A* on section from week 6 and week 8 of human adrenal gland. Scale bar on the insets is 5 µm.

Supplementary Figure 3

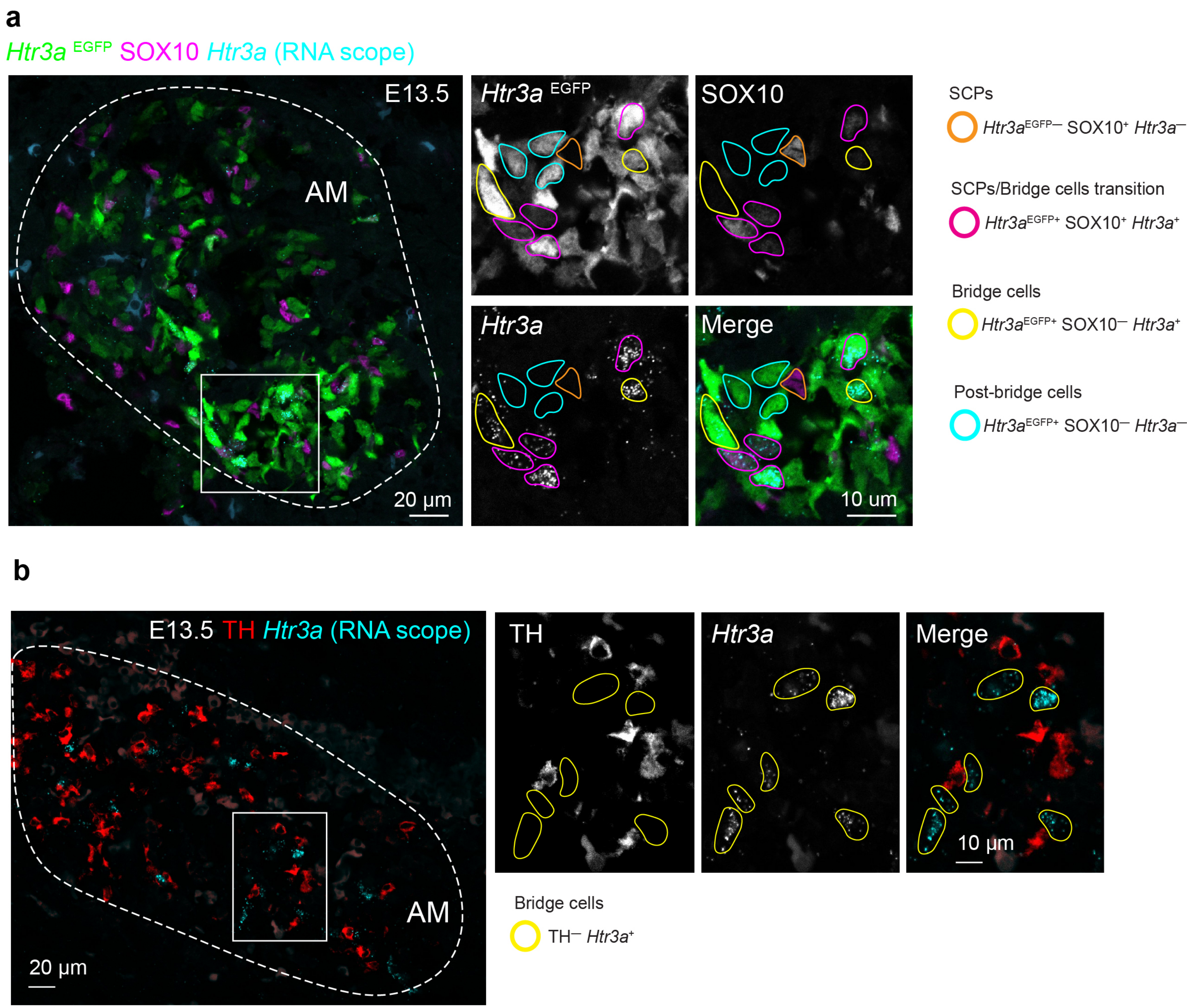

Supplementary Fig. 3 *Htr3a in situ* hybridization is associated with *Htr3a*^{EGFP}, SOX10 and TH expression. a, Combination of *Htr3a* mRNA *in situ* hybridization with immunohistochemistry for EGFP (recapitulating expression of *Htr3a*^{EGFP}) and SOX10 (a marker of Schwann cell precursors) on transversal sections of E13.5 adrenal gland from *Htr3a*$^{EGFP+/-}$ embryos. Some SCPs start expressing *Htr3a* mRNA at the SCPs/bridge cell transition. EGFP is retained in cells after active expression of *Htr3a* mRNA as indicated for "post-bridge" cells. **b**, Combination of *Htr3a* mRNA *in situ* hybridization with immunohistochemistry for TH (marker of chromaffin cells) shows that chromaffin cells do not actively express *Htr3a* mRNA. Adrenal medulla is outlined by a dashed line.

Supplementary Figure 4

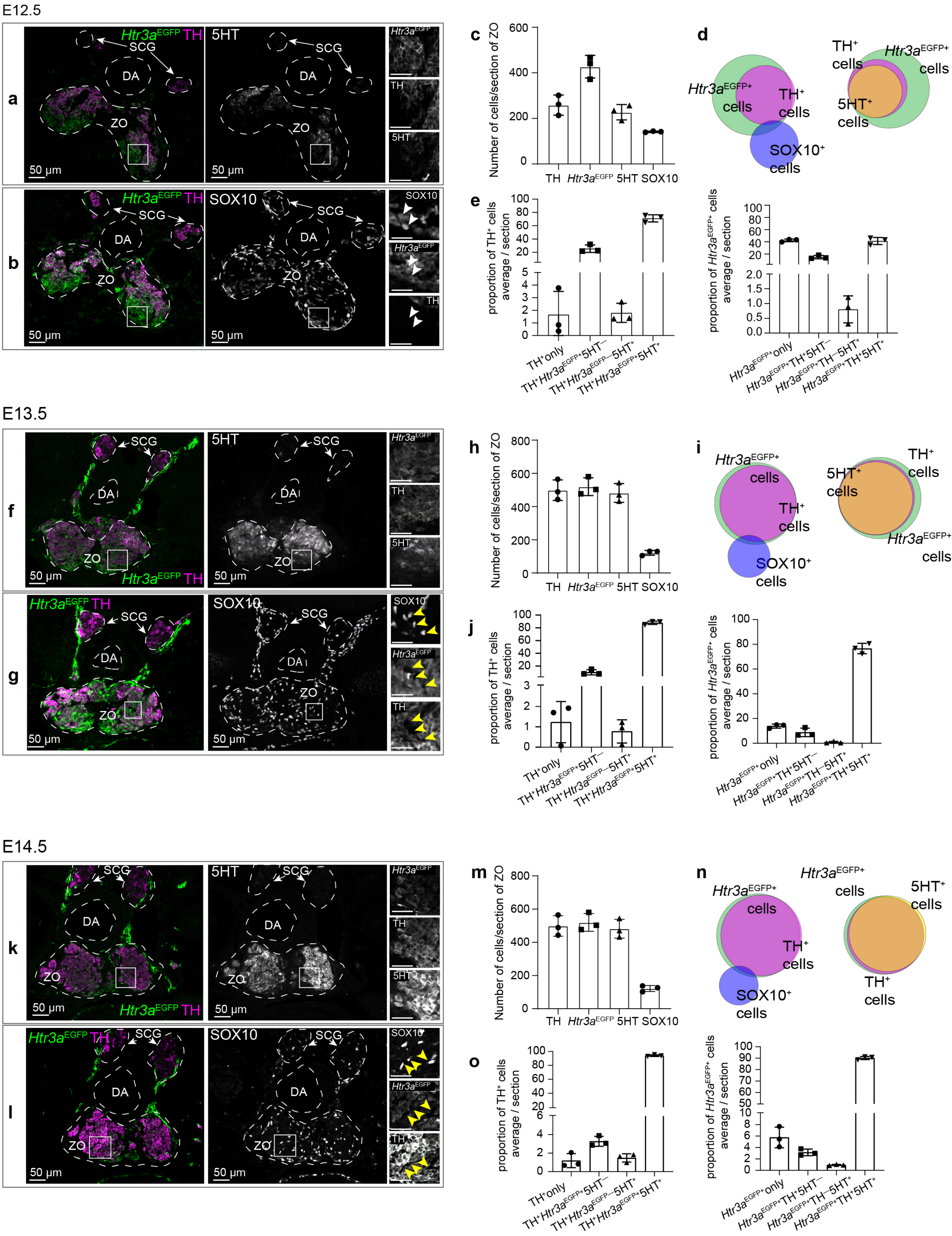

Supplementary Fig. 4 5HT-sensitive "bridge" cells and $5HT^{+}$ chromaffin cells are present in early Organ of Zuckerkandl. a, b Transversal section of Organ of Zuckerkandl (ZO) of *Htr3a*$^{EGFP+/-}$ embryos immunostained for TH (marker of chromaffin cells and sympathoblasts), EGFP (indicating expression of *Htr3a*) and either 5HT (5-hydroxytryptamine, serotonin) (**a**) or SOX10 (marker of SCPs) (**b**) at E12.5. White arrows point at $SOX10^{+}$/*Htr3a*$^{EGFP+}$/TH^{-} cells (**b**), indicating rapid generation of "bridge" cells. **c,** Cell numbers at E12.5. **d**, Venn diagrams of *Htr3a*$^{EGFP+}$, TH^{+}, $SOX10^{+}$ and $5HT^{+}$ cells at E12.5, indicating that 5HT expression is associated with the onset of TH expression. **e,** Proportions of *Htr3a*$^{EGFP+}$ and/or $5HT^{+}$ TH^{+} chromaffin cells (left plot), and proportions of TH^{+} and/or $5HT^{+}$ *Htr3a*$^{EGFP+}$ cells (right plot) at E12.5. **f, g** Immunohistochemistry on transversal sections of ZO of *Htr3a*$^{EGFP+/-}$ embryos stained for TH, EGFP and either 5HT (**f**) or SOX10 (**g**) at E13.5. Inserts in **f** show co-localization of *Htr3a*$^{EGFP+}$, TH^{+}, and $5HT^{+}$ territories. Yellow arrows point at $SOX10^{+}$/*Htr3a*$^{EGFP-}$/TH^{-} cells (**g**), indicating that SOX10 is not involved in "bridge" cell differentiation at E13.5. **h,** Cell numbers at E13.5. **i**, Venn diagrams of *Htr3a*$^{EGFP+}$, TH^{+}, $SOX10^{+}$ and $5HT^{+}$ cells at E13.5. **j,** Proportions of *Htr3a*$^{EGFP+}$ and/or $5HT^{+}$ TH^{+} chromaffin cells (left plot), and proportions of TH^{+} and/or $5HT^{+}$ *Htr3a*$^{EGFP+}$ cells (right plot) at E13.5. **k, l** Immunohistochemistry on transversal sections of ZO of *Htr3a*$^{EGFP+/-}$ embryos for TH, EGFP and either 5HT (**k**) or SOX10 (**l**) at E14.5. Inserts in **k** show co-localization of *Htr3a*$^{EGFP+}$, TH^{+}, and $5HT^{+}$ territories. Yellow arrows point at $SOX10^{+}$/*Htr3a*$^{EGFP-}$/TH^{-} cells (**l**) indicating the absence of "bridge" cells that may differentiate towards chromaffin cells. **m,** Cell numbers at E14.5. **n**, Venn diagram of *Htr3a*$^{EGFP+}$, TH^{+}, $SOX10^{+}$ and $5HT^{+}$ cells at E14.5. **o,** Proportions of *Htr3a*$^{EGFP+}$ and/or $5HT^{+}$ TH^{+} chromaffin cells (left plot), and proportions of TH^{+} and/or $5HT^{+}$ *Htr3a*$^{EGFP+}$ cells (right plot) at E14.5. Scale bars for insets=10 um. Quantifications are presented as Mean ± SD, biological n=3, technical n=3. Organ of Zuckerkandl (ZO), dorsal aorta (DA) and sympathetic chain ganglion (SCG) are outlined by a dashed line on all sections.

Supplementary Figure 5

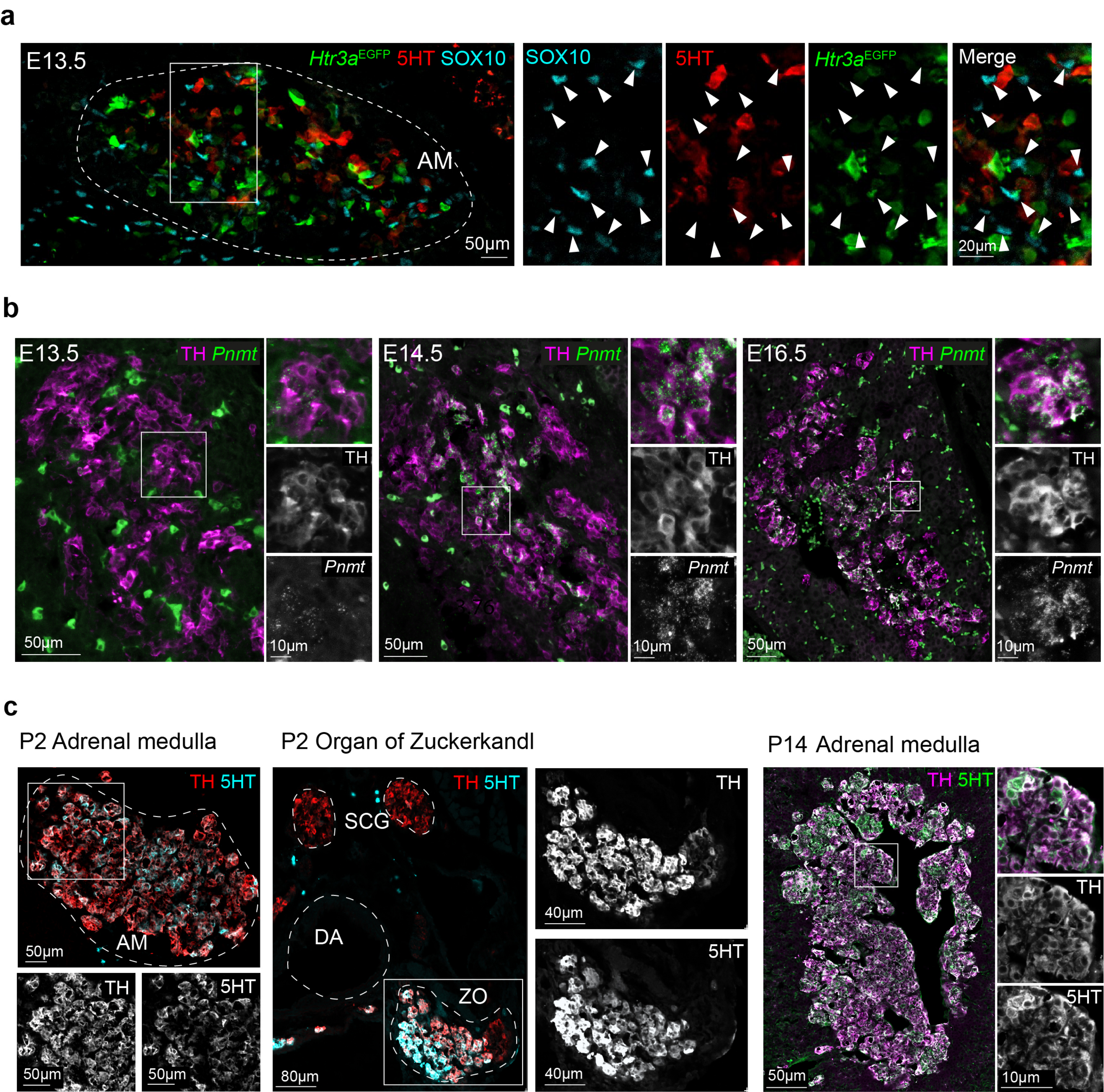

Supplementary Fig. 5 5HT content of SCPs and TH cells and chromaffin cell maturation. a, Immunohistochemistry on transversal sections of adrenal glands from E13.5 *Htr3a*$^{EGFP+/-}$ embryos for SOX10 (marker of SCPs), EGFP (indicating expression of *HTR3A*), and 5HT (5-hydroxytryptamine, serotonin). Arrowheads point at SOX10^{+}/*Htr3a*$^{EGFP-}$/5HT^{-} cells, indicating that SCPs are 5HT^{-} in adrenal glands. Adrenal medulla is outlined. **b**, Immunohistochemistry on transversal sections of adrenal glands from E13.5, E14.5, and E16.5 *Htr3a*$^{EGFP+/-}$ embryos for TH, EGFP combined with mRNA *in situ* hybridization for *Pnmt*. Note the onset of *Pnmt* expression at E14.5. **c,** Immunohistochemistry on transversal sections of adrenal glands and Organ of Zuckerkandl at postnatal day (P)2 and P14 for TH and 5HT. Note that all chromaffin cells at this stage are 5HT^{+}.

Supplementary Figure 6

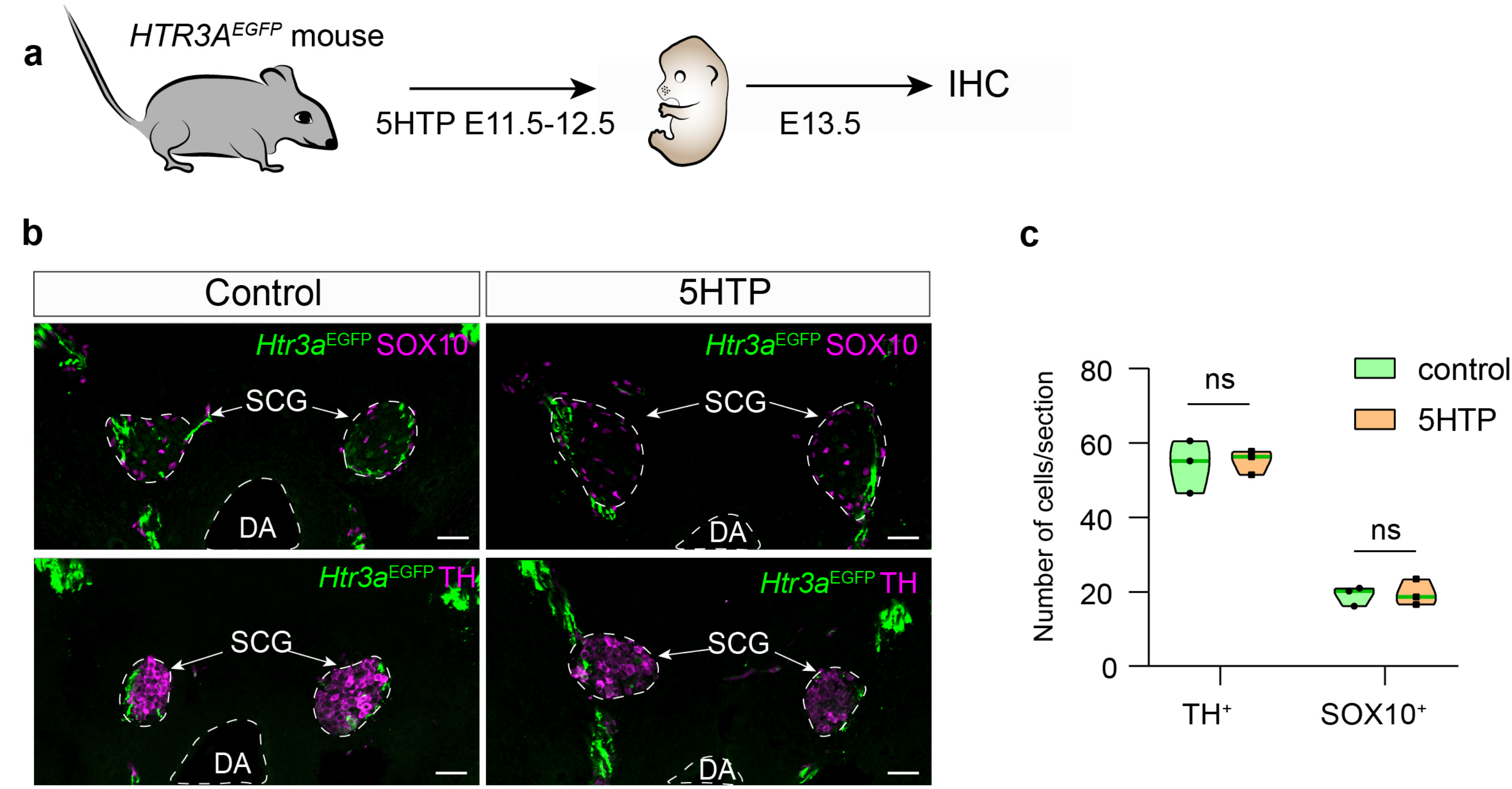

Supplementary Fig. 6 Sympathetic chain ganglia (SCG) of E13.5 embryos from 5HTP-treated females are not different in size in comparison with SCG from control embryos. a, Experimental design: 5HTP (5-hydroxytryptophan, the immediate precursor of serotonin) is administered to pregnant *Htr3a*$^{EGFP+/-}$ females at E11.5-E12.5. The embryos are collected at E13.5 for IHC-based analysis of cell numbers. **b**, Immunohistochemistry on transversal sections through sympathetic chain ganglia (SCG) for TH (marker of chromaffin cells and sympathoblasts), EGFP (indicating expression of *HTR3A*), and SOX10 (marker of SCPs) of E13.5 *Htr3a*$^{EGFP+/-}$ embryos collected from *Htr3a*$^{EGFP+/-}$ females from control and 5HTP-treated groups. **c**, Cell numbers are presented as violin plots, where green line indicates median, biological n=3, technical n=6. Shapiro-Wilk test for normality and unpaired double-sided t-test *p*-value ns > 0.05. Note: cell composition and the number of cells in SCG did not change in experimental conditions. Sympathetic chain ganglia (SCG) and dorsal aorta (DA) are outlined by dashed lines.

Supplementary Figure 7

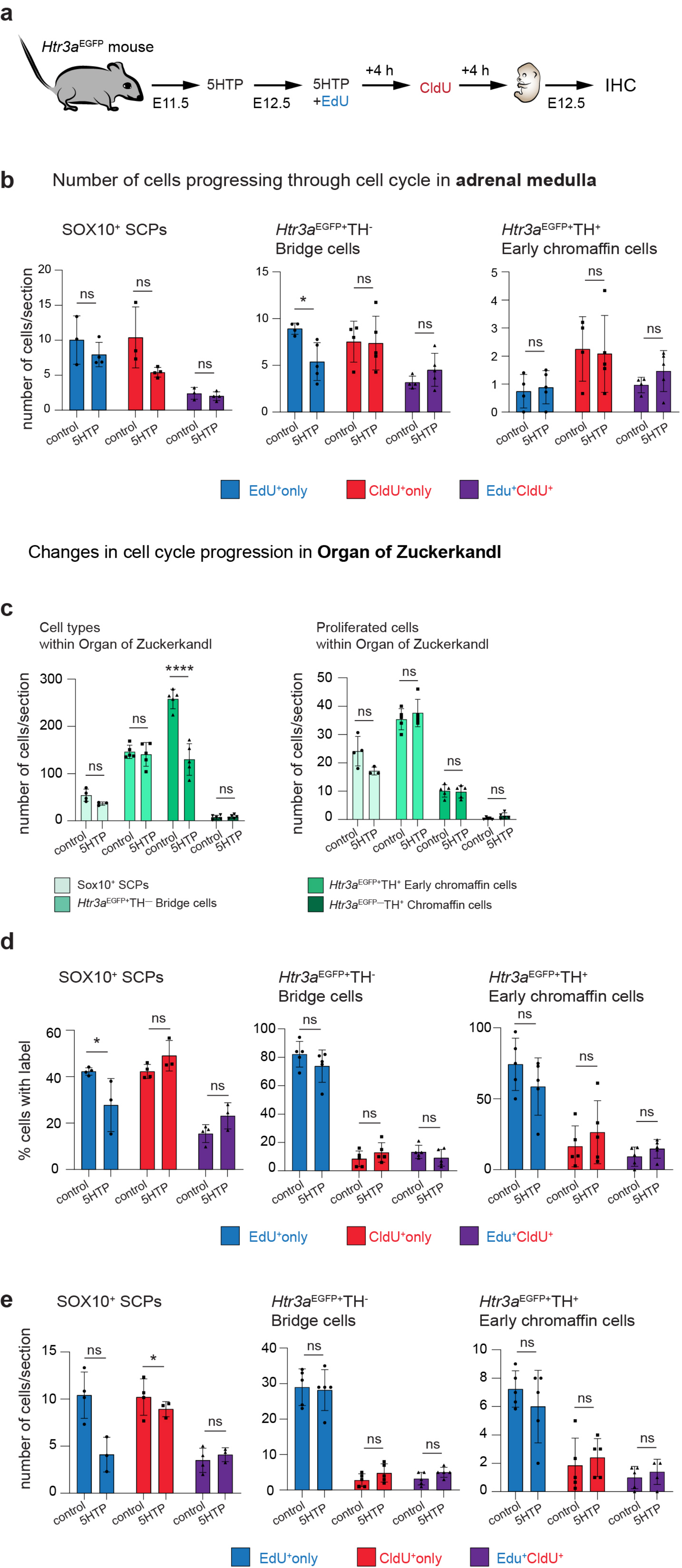

Supplementary Fig. 7 S-phase dynamics in adrenal medulla (AM) and Organ of Zuckerkandl (ZO). a, Pregnant *Htr3a*$^{EGFP+/–}$ females were administered 5HTP at E11.5; at E12.5, females received 5HTP together with 5-Ethynyl-2'-deoxyuridine (EdU); in 4 hours females received 5-Chloro-2′-deoxyuridine (CldU); embryos were harvested in 4 hours after CldU injection at E12.5. This allows identifying the proportions of cells, which incorporated EdU$^+$ only, CldU$^+$ only, or both thymidine analogues (EdU$^+$CldU$^+$). **b,** Numbers of EdU$^+$only, CldU$^+$ only, EdU$^+$CldU$^+$ cells in populations of SOX10$^+$ SCPs, *Htr3a*$^{EGFP+}$/TH$^–$ "bridge" cells and *Htr3a*$^{EGFP+}$/TH$^+$ early chromaffin cells under the influence of 5HT in AM. Shapiro-Wilk test for normality, unpaired double-sided t-test *p*-value ns > 0.05, * < 0.05, biological n = 3 (control SCPs), 4 (control other cell types), 4 (5HTP SCPs), 5 (5HTP other cell types). **c**, Numbers of all cells (left panel), numbers of proliferated cells (right panel) in populations of SOX10$^+$ SCPs, *Htr3a*$^{EGFP+}$/TH$^–$ "bridge" cells, *Htr3a*$^{EGFP+}$/TH$^+$ early chromaffin cells, and *Htr3a*$^{EGFP–}$/TH$^+$ mature chromaffin cells at E12.5 under the influence of 5HTP in ZO. Shapiro-Wilk test for normality, unpaired double-sided t-test *p*-value **** < 0.0001, biological n = 4 (control SCPs), 5 (control other cell types), 3 (5HTP SCPs), 5 (5HTP other cell types). Note: the number of early chromaffin cells (*Htr3a*$^{EGFP+}$/TH$^+$) decreased significantly under 5HTP treatment, but overall proportions of proliferated cells remain unaffected. **d**, Proportions of EdU$^+$only, CldU$^+$ only, and EdU$^+$CldU$^+$ cells in populations of SOX10$^+$ SCPs, *Htr3a*$^{EGFP+}$/TH$^–$ "bridge" cells and *Htr3a*$^{EGFP+}$/TH$^+$ early chromaffin cells at E12.5 under the influence of 5HTP in ZO. Note: cell cycle is lengthened among SCPs, as there are significantly less EdU$^+$only SOX10$^+$ SCPs. Shapiro-Wilk test for normality, unpaired double-sided t-test *p*-value ns > 0.05, * < 0.05, biological n = 4 (control SCPs), 5 (control other cell types), 3 (5HTP SCPs), 5 (5HTP other cell types). **e**, Number EdU$^+$only, CldU$^+$ only, and EdU$^+$CldU$^+$ cells in populations of SOX10$^+$ SCPs, *Htr3a*$^{EGFP+}$/TH$^–$ "bridge" cells and *Htr3a*$^{EGFP+}$/TH$^+$ early chromaffin cells at E12.5 under the influence of 5HTP in ZO. Shapiro-Wilk test for normality, unpaired double-sided t-test *p*-value ns > 0.05, * < 0.05, biological n = 4 (control SCPs), 5 (control other cell types), 3 (5HTP SCPs), 5 (5HTP other cell types).

Supplementary Figure 8

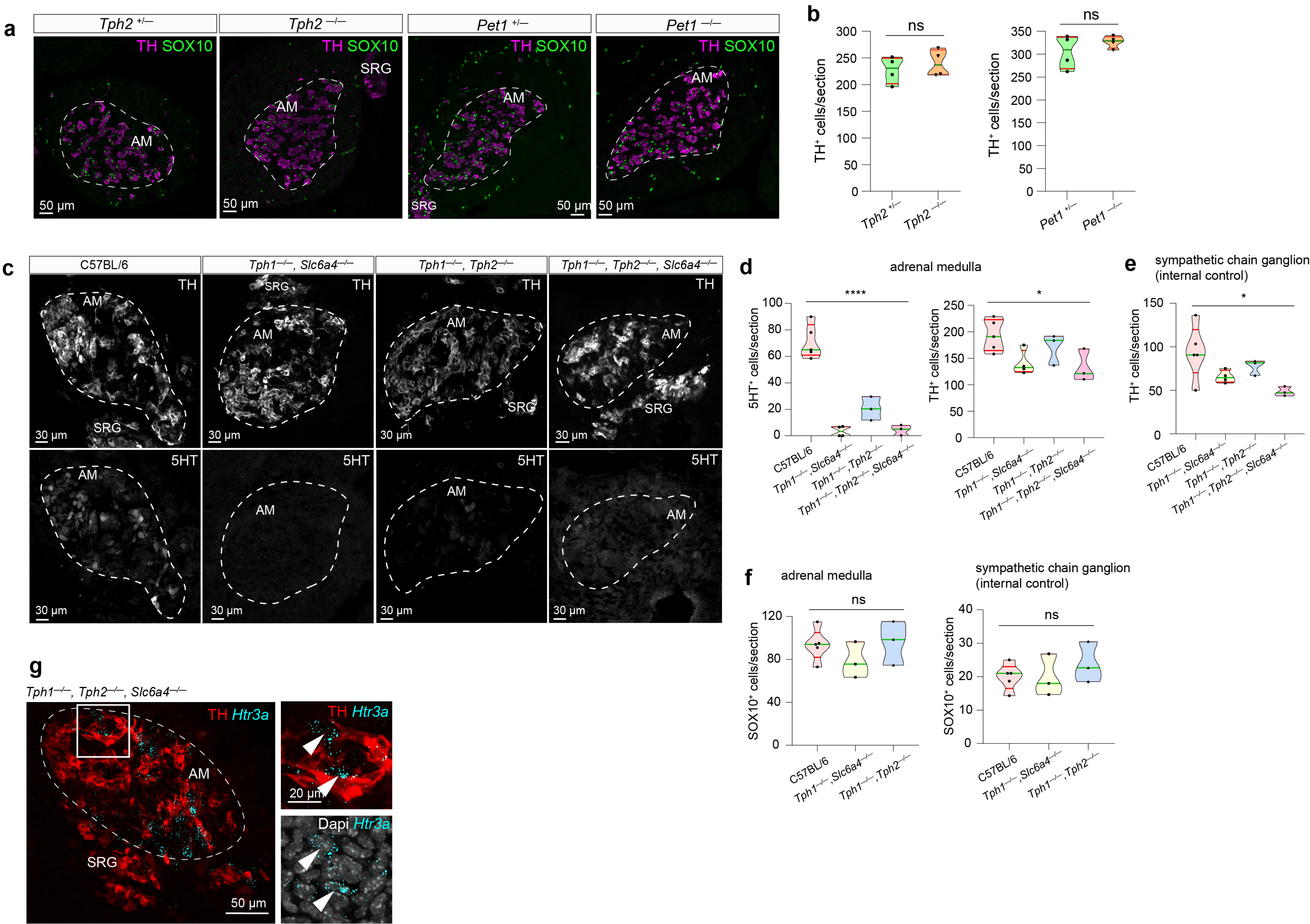

Supplementary Fig. 8 Genetic depletion of serotonin biosynthesis does not affect chromaffin cells beyond general growth retardation. **a**, Immunohistochemistry for TH and SOX10 on transversal sections through the adrenal gland of E15.5 *Tph2*$^{-/-}$ and *Tph2*$^{+/-}$ embryos; E15.5 *Pet1*$^{-/-}$ and *Pet1*$^{+/-}$ embryos . **b**, TH^{+} cell numbers in *Tph2*$^{-/-}$, *Tph2*$^{+/-}$ and *Pet1*$^{-/-}$, *Pet1*$^{+/-}$ are presented as violin plots, where green line – median, biological n=4, technical n=3-4. Shapiro-Wilk test for normality, unpaired double-sided t-test *p*-value ns > 0.05. **c**, Immunohistochemistry for TH and 5HT on transversal sections through the adrenal gland of E13.5 embryos of C57BL/6, E13.5 *Tph1*$^{-/-}$,*Scl6a4*$^{-/-}$ embryos collected from *Tph1*$^{-/-}$,*Scl6a4*$^{-/-}$ females, E13.5 *Tph1*$^{-/-}$,*Tph2*$^{-/-}$ embryos collected from *Tph1*$^{-/-}$,*Tph2*$^{-/-}$ females and E13.5 *Tph1*$^{-/-}$,*Tph2*$^{-/-}$,*Scl6a4*$^{-/-}$ embryos collected from *Tph1*$^{-/-}$,*Tph2*$^{-/-}$,*Scl6a4*$^{-/-}$ females. **d**, 5HT^{+} and TH^{+} cell numbers in adrenal medulla (AM) are significantly reduced. Cell numbers are presented as violin plots, where green line - median, red lines - quartiles. Ordinary one-way ANOVA *p*-value * < 0.05, **** < 0.0001. **e**, Quantification of TH^{+} cells on sections of sympathetic chain ganglia (SCG; internal control) shows similar reduction pattern indicating the general reduction of embryo size. Cell numbers are presented as violin plots, green line - median and red lines - quartiles. Ordinary one-way ANOVA *p*-value * < 0.05, biological n=5 (C57BL/6), 4 (*Tph1*$^{-/-}$,*Scl6a4*$^{-/-}$), 3 (*Tph1*$^{-/-}$,*Tph2*$^{-/-}$and *Tph1*$^{-/-}$,*Tph2*$^{-/--}$,*Scl6a4*$^{-/-}$) **f**, Quantification of SOX10^{+} cells on sections of AM and SCG (internal control) shows no change between in C57BL/6 embryos and KO embryos. Cell numbers are presented as violin plots, green line - median and red lines - quartiles. Ordinary one-way ANOVA *p*-value ns > 0.05, biological n= 5 (C57BL/6), 3 (*Tph1*$^{-/-}$,*Scl6a4*$^{-/--}$), 3 (*Tph1*$^{-/--}$,*Tph2*$^{-/-}$) **g**, Combination of immunohistochemistry for TH and mRNA *in situ* hybridization for *Htr3a* on transversal sections of adrenal gland of E13.5 *Tph1*$^{-/-}$,*Tph2*$^{-/--}$,*Scl6a4*$^{-/-}$ shows *Htr3a* expression in knockout embryos. Adrenal medulla (AM) is outlined by dashed line on all sections, SRG – suprarenal ganglion, arrows point at *Htr3a*$^{+}$/TH^{-} cells.

Supplementary Figure 9

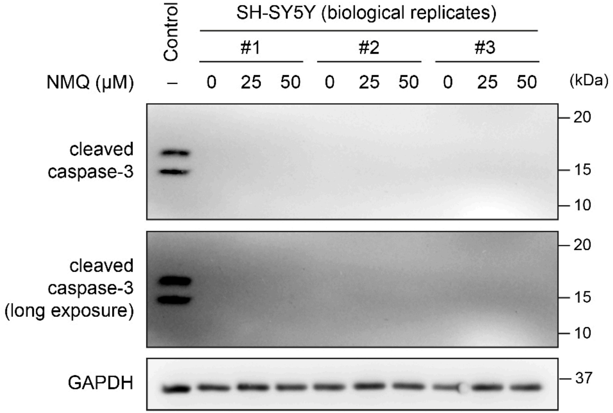

Supplementary Fig. 9: The HTR3A agonist N-methylquipazine dimaleate (NMQ) does not induce apoptosis in HTR3A^{high} SH-SY5Y cells. Western blotting analysis of whole cell extracts from SH-SY5Y cells treated with either vehicle (0 µM) or NMQ (25 µM and 50 µM) for 5 days. CHLA-20 cells treated with 100 nM staurosporine for 24 h served as a positive control for apoptosis induction (Control). Cleaved caspase-3, a marker of apoptosis, was not detected in NMQ-treated cells. GAPDH served as a loading control.

Uncropped western blot scans for Supplementary figure 9

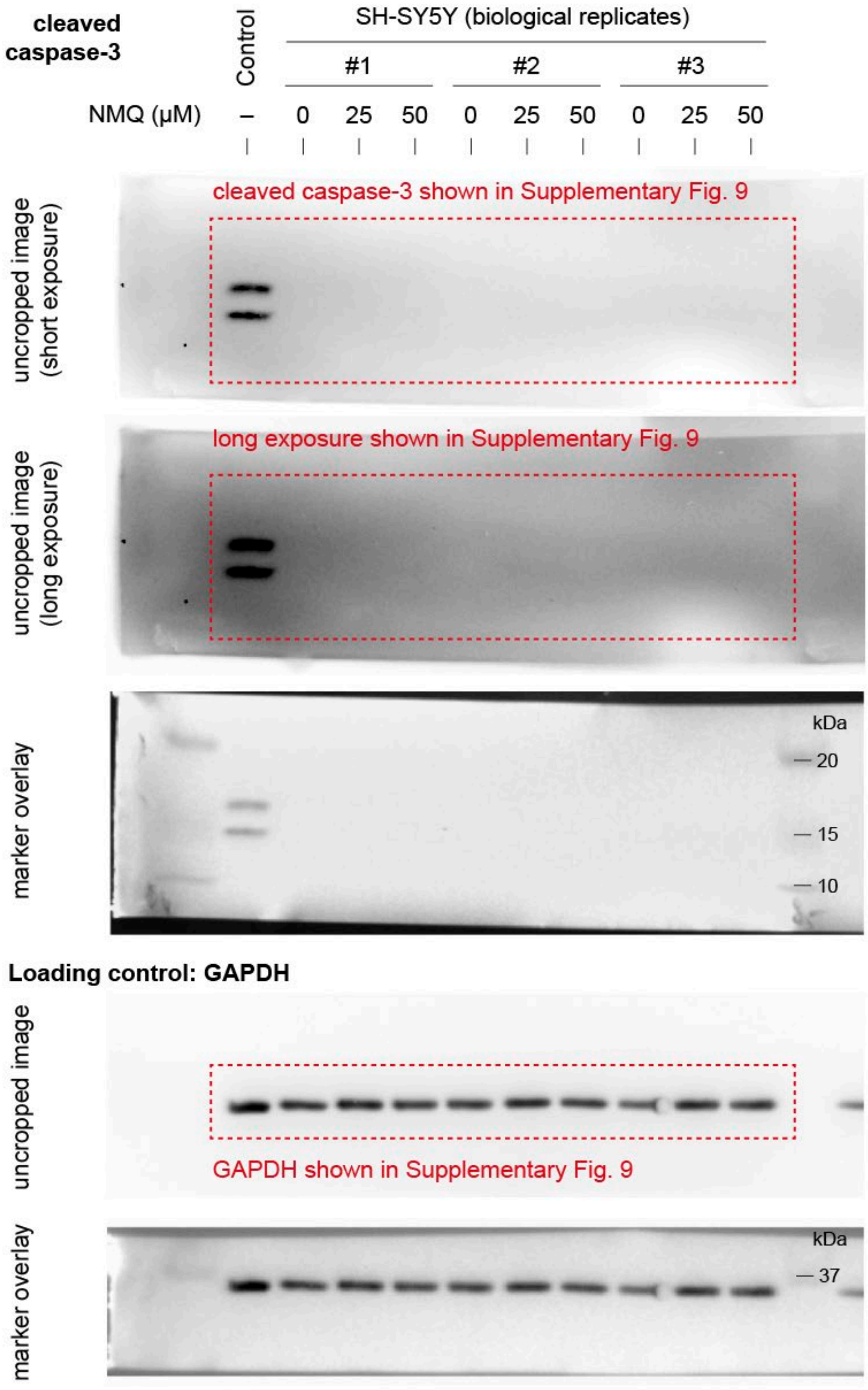

Supplementary Figure 10

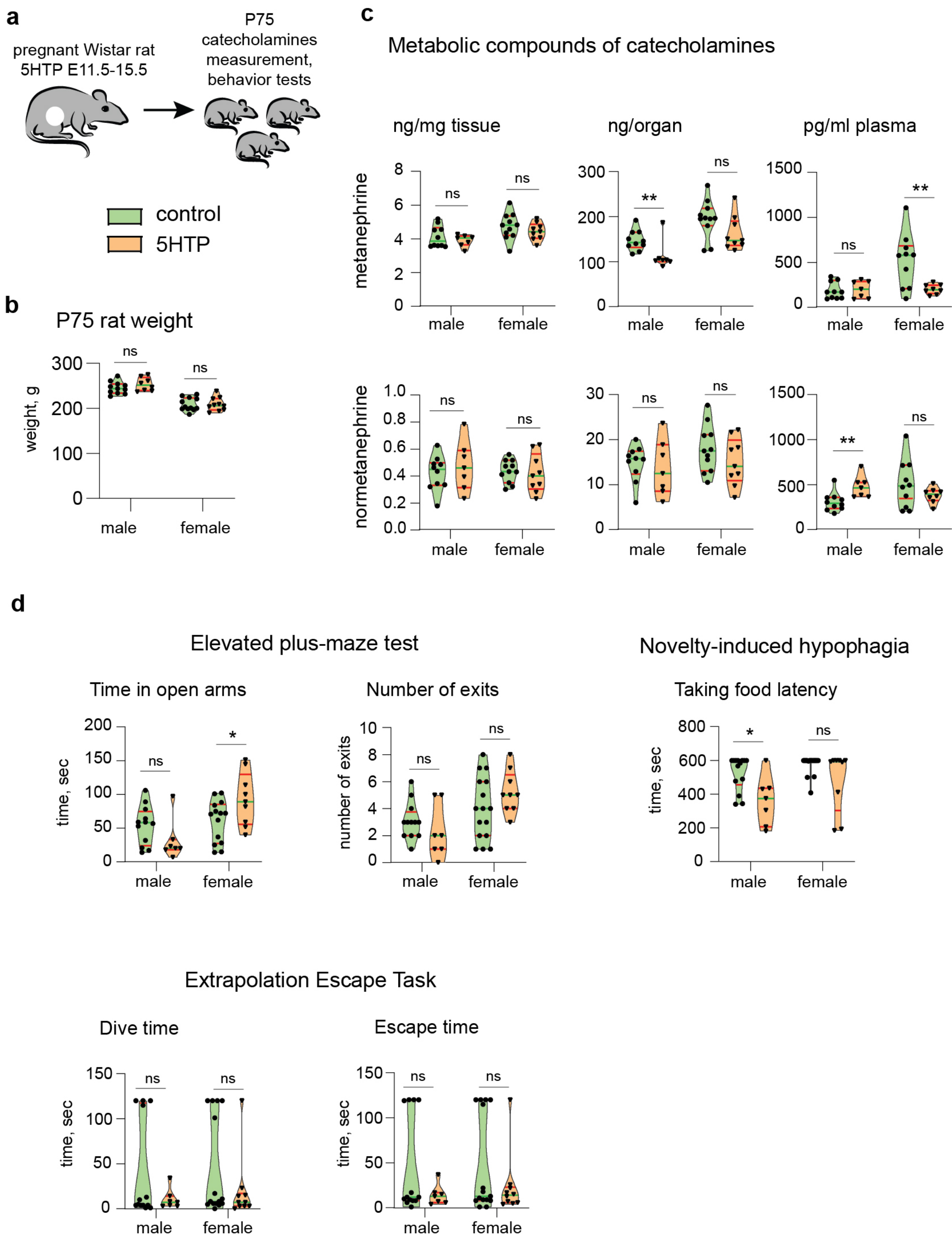

Supplementary Fig. 10 Catecholamine metabolites and anxiety behavior tests of P75 rats prenatally treated with 5HTP. a, Pregnant Wistar rats were administered 5HTP during E11.5-E15.5, and their offspring was allowed to grow up to post-natal day (P) 75 before behavioral tests and catecholamine measurements. **b**, The weight of the animals born from females in 5HTP-treated group was not different from weight of animals in the control group. The green line in violin plots indicates median, and the red lines indicate quartiles. Mann Whitney test, *p*-value ns > 0.05, biological n = 11 (male control), 8 (male 5HTP), 12 (female control), 10 (female 5HTP). **c**, Levels of catecholamine metabolites measured in ng per mg of tissue, ng per organ and pg per ml of plasma by HPLC. No increase of metanephrine was observed, while normetanephrine was slightly increased in males in pg per ml of plasma. Therefore, reduction of adrenaline is not due to catabolism of catecholamines. Green line on violin plots indicates median, red lines indicate quartiles Mann Whitney test, *p*-value ns > 0.05, ** < 0.002, biological n = 11 (male control), 8 (male 5HTP), 12 (female control), 10 (female 5HTP), for plasma measurement n = 10 (male control), 7 (male 5HTP), 10 (female control), 8 (female 5HTP). **d**, Effect of prenatal 5HTP treatment in behavioral tests: elevated plus maze test, novelty-induced hypophagia, and extrapolation escape task. Females in the 5HTP-treated group spend significantly more time in open arms of the maze. Novelty-induced hypophagia test shows that males in the 5HTP-treated need less time to start taking food in unknown environment. In extrapolation escape task both 5HTP-treated male and female groups lack the animals that take longer time to dive and to escape indicating even though the change is not significant. Mann Whitney test, *p*-value ns > 0.05, *< 0.05 biological n= 11 (male control), 8 (male 5HTP), 12 (female control), 10 (female 5HTP). Data presented as violin plots, where green line - median and red lines - quartiles
