# Supplementary material for: Serotonin limits generation of chromaffin cells during adrenal organ development
Source: Nat Commun. 2022 May 25;13:2901. doi: 10.1038/s41467-022-30438-w (PMC9133002; doi:10.1038/s41467-022-30438-w)
Supplement: Supplementary file 3 — Peer review [file 41467_2022_30438_MOESM3_ESM.pdf]

nature portfolio

Peer Review File

SEROTONIN LIMITS GENERATION OF CHROMAFFIN CELLS DURING ADRENAL ORGAN DEVELOPMENT

Open Access This file is licensed under a Creative Commons Attribution 4.0 International License, which permits use, sharing, adaptation, distribution and reproduction in any medium or format, as long as you give appropriate credit to the original author(s) and the source, provide a link to the Creative Commons license, and indicate if changes were made. In the cases where the authors are anonymous, such as is the case for the reports of anonymous peer reviewers, author attribution should be to 'Anonymous Referee' followed by a clear attribution to the source work. The images or other third party material in this file are included in the article's Creative Commons license, unless indicated otherwise in a credit line to the material. If material is not included in the article's Creative Commons license and your intended use is not permitted by statutory regulation or exceeds the permitted use, you will need to obtain permission directly from the copyright holder. To view a copy of this license, visit http://creativecommons.org/licenses/by/4.0/.

REVIEWER COMMENTS

Reviewer #1 (Remarks to the Author):

This is a very interesting manuscript that describes how serotonin affects the generation of chromaffin cells during adrenal gland development. The authors discuss how adrenal gland development is mediated by a novel feedback loop based on paracrine signaling when serotonin and its receptor are released by early, mature chromaffin cells. The overall direction of this manuscript is very positive, however, there are several problems that need to be addressed and are outlined below. There are statements that need corrections and tone adjustments, and the authors should focus on their area of expertise, which is developmental biology.

The introduction is too long and at times, hard to follow. The authors introduced too much information without a clear, logical sequence, which is confusing to the reader.

Line 80-81: The statements about the bladder should be deleted. The following sentence could be moved to the end of the introduction where the authors outline their hypothesis and objectives.

Furthermore, the statements and expressions used makes it clear that the authors have limited knowledge in certain fields. For example, in line 96-97, the authors use the word “emergence.” This word is almost never used when introducing or discussing information about tumors (specifically in this manuscript) and the authors should consider re-phrasing this. For example, when the authors describe neuroblastomas, I would recommend using the phrase “where these tumors are found.”

Line 96: “Most importantly” should be changed to “Furthermore” in order to enhance readability and flow.

When discussing the organ of Zuckerkandl, the authors should understand that this is a site for paraganglioma, not pheochromocytoma. Furthermore, neuroblastomas are mainly found in the adrenal gland, not the organ of Zuckerkandl. This is a weakness of the study since no pheochromocytoma cell lines were used when studying chromaffin cells (there are several pheochromocytoma cell lines that are readily available, such as MPC, PC12, MTT, and human pheo1). One of these cell lines will need to be added in present experiments and approaches.

Line 109: “the main neuroendocrine adrenergic organ” should be changed to “the main neuroendocrine organ” to reduce over-wording.

Results/discussion:

Line 149-155: The statement related to catecholamine production and PNMT must also be supported by the results related to tyrosine hydroxylase (see extended Fig. 3a-d). Furthermore, the statement related to the release of catecholamines and serotonin (lines 154-155) may not be accurate. The presence of an enzyme does not guarantee that, for example, catecholamines will be released.

Fig. 3f: Sympathetic ganglia is a very broad term and the authors should clarify which sympathetic ganglia they are talking about, and change the figure descriptions accordingly. What is the difference between sympathetic ganglia and mesenteric ganglion (line 231)?

What is the likely explanation of differences between the AM and ZO experiments shown in Figure 3? In other words, why did 5HT significantly changed early chromaffin cells in the AM but not in the ZO? I get the feeling that the authors circumvent explanations of "complicated" results and only focus on data that is nicely obtained from their experiments (e.g. see my comments related to catecholamines and anxiety tests in animals). Furthermore, the number of early chromaffin cells is increased in figure 3 (cartoon - OZ).

Line 284-286 should indicate 6c, not 6e. The results are expressed as ng/organ, however, the weight of the adrenal glands differs so this data is incorrect. It should be expressed per protein (e.g. mg) or per weight (e.g. ng). The plasma data is not useful and does not correlate with the tissue data – what did the authors want to say here? Is there a meaningful physiological impact? If there is an effect of serotonin, differences between males and females, differences between organs (although, these were not measured correctly) and differences in plasma would be expected, however, there is no consistent trend and the results are not convincing. Moreover, the data between noradrenaline and normetanephrine do not match. This is confusing.

I am very confused with the results from the pharmacological study. In the conclusion, the authors state: "These results are in line with the in vivo measurements and indicated the persistant reduction of stress hormones which are released from the chromaffin cells in animals prenatally exposed to the elevated 5-HT level." This is not true. Furthermore, the sentence is very difficult to understand and reflects practically no specific data. In males, differences have not been seen in plasma adrenaline, only mild decreases in noradrenaline, no supporting data for normetanephrine, and the behavioral studies on 6e-g oppose the catecholamine data. Males showed changes (compared to females) in the hypophagia test and escape task, however, these differences were not discussed or explained. Experimental animals quickly found ways to escape from water, however, this group had lower adrenaline and noradrenaline in either the adrenal gland or plasma. Your data does not fit. Are the authors trying to say that lower catecholamines reflect better and quicker escape plans, or the ability of an animal to free itself to obtain food, or something else? This is extremely confusing and needs a very good explanation. This also applies to the abstract (line 48) where the statements do not add value or help educate the reader. Figure 6f and 6g could show data for females first to keep consistent with figure 6c.

The data related to neuroblastoma cells is interesting and relevant, however, the authors describe that their results support the idea that neuroblastomas might resemble bridge progenitor cells. In

the experiments with AM, 5HTP decreases early chromaffin cells but not bridge cells (although, the cell number related to bridge cells in figure 3 is in question – did they decrease?), thus, your conclusion may not fit. Perhaps early chromaffin cells can be affected instead of bridge cells? If only proliferation is decreased, what would the effect of N-methylquipazine be on tumor growth in NSG mice?

The statement in lines 318 – 322 is not a useful way to begin a discussion. The authors should first directly state the best outcomes and results of the present study. Starting out with the stress response and avoidance behavior is not very tactical because this is a weak part of the study. You presented excellent results in other parts of this study, so the authors should focus on these. The same goes for the "migration" data – you did not study it here so it should not be emphasized.

If 5HT is increased in pregnant women, the authors should state its clinical relevance. As described on page 16, there are clinically relevant situations and the authors should focus on which ones are most important. Mild stress, moderate hypoxia, and vitamins have not been proven to be of major importance in adrenal gland-related tumors. The authors should attempt to treat animals with vitamins or exposed them to stressful conditions and see if they get similar results to their experiments. This would support their data and it would greatly contribute to the pathophysiology of adrenal tumors.

The statement in lines 386-287 is of interest but lacks the supporting data. You would need to perform the appropriate experiments, which could be feasible (line 392-393).

I am not sure whether the statements in line 396-405 support your data. It seemed to be contradictory when I read it.

Were the histopathological sections of the adrenal glands the same size? Note that adrenal gland (medulla) is changing the size. How were all of the results calculated?

The adrenal gland is not a major source of norepinephrine.

There are too many abbreviations, making it very difficult for readers to orient themselves when reading the text. I would recommend that the authors make a table with all of the abbreviations listed. Moreover, when you talk about TH+ cells in line 219 and figure 3f, do you mean both early chromaffin cells and mature chromaffin cells? As mentioned above – what are the differences between sympathetic ganglia and mesenteric ganglion? There are no differences to my best knowledge. If there are no differences, why are they listed separately?

Why is figure number 3 placed before figure number 2 in the text?

Minor:

Misspelling: catecholamine – in extended figure 1c.

Delete pediatric in line 385.

Decide whether you want to use sympathoadrenal or sympatho-adrenal and stay consistent.

Line 123: put the abbreviation for TH here.

Line 286: Figure 6e does not describe catecholamine levels.

Reviewer #2 (Remarks to the Author):

This paper presents interesting insight to the formation and functionality of chromaffin organs. The manuscript is based on previous work describing the transcriptional landscape and heightened expression of the serotonin receptor HTR3A in an intermediate “bridging cell” which links the developmental transition from schwann cell precursor to chromaffin cell. Overall the work is well presented and thorough. Whilst there are some inconsistencies between the mechanistic findings and neuroblastoma the work should be of wide interest.

Major comments:

1. The authors suggest a time-window in which 5-HT may play a role on the bridge cells to regulate chromaffin development. This is based on expression of EGFP driven from the Htr3a promoter. As the GFP is stable for some time, this work would be strengthened by repeating with antibodies and RNA expression analysis. Even better would be to treat pregnant mice with excessive 5-HTP at different ages to test this.

2. All of the histological analyses are performed in mice with rats used for many of the functional analyses. There needs to be at least some investigation in rat embryogenesis to provide confidence that the same developmental mechanisms are conserved across species. Analysis of adrenal glands

in rat embryos is limited to females yet males and females are included in the adult studies. There are also inconsistencies between the number of rat embryos used for microCT and histology – n=4 and 3 for these embryonic studies is low considering entire litters could be used. The decrease in area by microCT in rat embryos also outweighs the reduction in size of the medulla. The number if embryos used in Fig 3 also needs clarification. The title infers only one pregnant female was used with n=3 embryos.

3. It remains unclear how increased 5-HT leads to a lack of chromaffin cells in the medulla. EdU incorporation suggests no alteration in the proliferation of bridge cells, schwann cells or chromaffin cells, yet there are less cells overall. Without increased cell death, how is the overall cell number reduced, not just the proportion? Prior studies of chromaffin cell development have shown changes in cell cycle length and proliferative capacity which may be worth investigation. A 24 hr chase may be too long to uncover any changes in this experiment. The schematics in Fig 3 tend to exaggerate the data a bit too much (in C bridge cells should be the same, while early chromaffin cells should not be increased in e). If bridge cells fail to differentiate what happens to them?

4. The link to neuroblastoma is not consistent. The models used are well characterised as neuroblast models so the finding that some have higher levels of Htr3a is interesting. What does this mean for their origin? Are these more precursors of SG, SCP, bridge cells? Activation of HTR3A had no effect on bridge cell proliferation in vivo, yet activation in SH-SY5Y reduced their proliferation, which is inconsistent. Does this make them differentiate? This data is taken to infer that the 5-HT feedback might prevent excessive organ growth and tumour formation. However, it could also be possible that increased 5-HT blocks differentiation, driving tumour formation. One notable observation in your work that is not discussed, and should be, is the increase in Normetanephrine (incorrectly spelt in Figure) in male 5-HTP treated rats. This is a marker of pheochormocytoma in humans, and should be discussed.

Minor suggestions:

1. The last sentence of the abstract is ambiguous.

2. More information is needed for the expression patterns used to define chromaffin cells (immature or mature), bridge cells and schwann cells in the single cell data.

3. Sox10 immunostaining is not clear in some images (Fig. 1). Consider increasing contrast so this is easier to visualise.

4. PNMT expression is not clear and should be repeated with antibody staining.

5. Was there any impact on male rat emrbyos?

6. Reference to Fig 6e and 6c needs to be checked.

7. Titles not needed in the figures.

Reviewer #3 (Remarks to the Author):

In their manuscript “Serotonin limits generation of chromaffin cells during adrenal organ development” Kameneva et al report a novel finding where chromaffin cells through 5HT signaling during a sensitive period of embryonic development exert a paracrine effect onto bridge cells to regulate chromaffin organ size and function. This work contributes important and novel information of relevance to multiple fields including psychiatry and oncology. The work is clearly presented and of high quality, however there are some important details that limit my enthusiasm. Suggestions for improvement of the work follow:

1. The main manipulation in the paper, s.c. 5-HTP 20 mg/kg (w/o peripheral decarboxylase inhibitor), is not studied with regards to its pharmacokinetic and pharmacodynamic characteristics. Two papers are referenced (Udenfriend et al., 1956; Mitchell et al., 1983), but neither of them assessed 5-HTP or 5-HT levels in the embryonic compartment. Without proper references or original data, this is a “black box” manipulation.

2. Regarding extended figure 1, the authors should clearly state that these data have been previously published (Furlan et al., 2018). (also no single cell analysis methods section).

3. Provide for extended figure 1 numerical quantification of gene expression levels averaged by cluster. This will help clarifying the following issues:

a. In extended Figure 1B it seems that Htr3a/3b is not only expressed in bridge cells but also in SCPs, PC, and sympathoblasts at E12.5 while at E13.5 this expression is restricted to bridge cells, PCs and some sympahtoblasts. The authors should report this in the results section.

b. In extended Figure 1C it seems that DDC+ is not restricted to chromaffin cells only but it is also expressed in other cell types, i.e. bridge cells, PCs, and sympathoblasts at E12.5 and at E13.5. The authors only discuss DDC expression for chromaffin cells (line122). How does DDC expression in other cell types affect the synthesis of 5HT at these developmental stages?

c. Expression of Slc29a4 as well as other monoamine transporter genes seem not restricted to chromaffin cells either, but to extend to other cell types. Quantification and appropriate discussion of these findings should be mentioned. How does expression of these genes in other cell types affect the chromaffin cell paracrine modulation and the overall hypothesis? Could it be that bridge cells also exert an autocrine modulation via 5HT signaling?

4. In Figure 1 it is not how 5-HT colocalizes (it is also missing from the Venn diagram). Please provide a percentage where the numerator is 5HT and the denominator is either TH or Sox10/EGFP. Also, the analysis of the cell count should be explained in more detail. The authors should report the percentages of overlap for every cell type quantification and the statistics on the percentages rather than total numbers.

5. Th is primarily expressed in chromaffin cells, however DDC seems to be expressed by a larger cell population including bridge cells based on the transcriptomic data. How do the authors explain that chromaffin cells are the primary source of 5HT when also other cells have the potential to synthetize 5HT given their expression of DDC (especially as Tph loss of function experiments do not affect Sox10 cell numbers)? How do the authors rule out a possible autocrine regulation of 5HT onto bridge cells? What is the proportion of Sox10/5HT/HTR3A at E13.5? For this reason, the authors should p

Reviewer #4 (Remarks to the Author):

In this study, Kameneva et al. propose a novel feedback loop regulating the production of mature chromaffin cells in the adrenal gland. According to the authors' model, Schwann cell precursor (SCP)-derived bridge cells act as chromaffin precursor cells that express the serotonin receptor 5-hydroxytryptamine receptor 3A (HTR3A). HTR3A, in turn, is activated by 5-HT secreted by early mature chromaffin cells. Upon increase of 5-HT levels, the generation of mature chromaffin cells is reduced and consequently the size of the adrenal gland decreased. This then correlates with altered expression of stress hormones and slight behavioural changes. Dissecting the impact of this signalling pathway in development of the adrenal gland may also pave the way for a better understanding of chromaffin organ-derived cancers, such as neuroblastoma.

This is a potentially interesting study, as the proposed regulatory feedback loop is novel. However, there are several major issues that need to be addressed prior to publication of this study. In particular, the data supporting a regulatory feedback loop between bridge cells and chromaffin cells need to be substantially strengthened. Likewise, the link between the findings made in development and those obtained from the tumorigenesis assays has to be better established.

Main points:

1. Although Htr3a is proposed by the authors to be expressed specifically in bridge cells, HTR3AEGFP also marks early chromaffin cells labelled by TH. In fact, at the relevant stages, the vast majority of Htr3aEGFP expression overlaps with TH expression (Fig. 1). Therefore, virtually all data could be interpreted as an effect of autocrine serotonin signaling on chromaffin cells (rather than by cross-regulation between bridge cells and chromaffin cells). Indeed, unlike claimed in the manuscript (lines 213ff), the proportion of Htr3aEGFP-positive cells is NOT increased in the adrenal medulla upon 5-HTP treatment (Fig. 3c), suggesting that the reduction of chromaffin cells in these mice is due to a direct effect of 5-HT on chromaffin cells. The proportion of Htr3aEGFP-positive cells is only (relatively slightly) increased in the organ of Zuckerkandl (Fig. 3e), but there, early chromaffin cell numbers are not changed, again suggesting that the phenotype is NOT coupled with altered differentiation of bridge cells.

In the experiments shown e.g. in Fig. 3f (pharmacological treatment) or Fig. 5, only TH+ chromaffin cells are taken into account; i.e. these experiments are in agreement with a direct effect of serotonin on chromaffin cells and don't support a role of paracrine serotonin signaling from chromaffin cells to bridge cells, as proposed in the authors' model.

All in all, the proposed regulatory feedback loop between bridge cells and chromaffin cells is a central part of this study; therefore, it needs to be experimentally substantiated, e.g. by demonstrating 5-HT dependent effects on differentiation of isolated (Htr3aEGFP+/TH-) bridge cells in the absence of 5-HT effects on TH+ chromaffin cells.

2. Along these lines: does HTR3A protein expression overlap with Htr3aEGFP expression at E13.5 or is EGFP maintained despite downregulated HTR3A protein expression? The latter case would possibly help to support the authors' model.

3. In Fig. 1, 5-HT expressing cells should be included in the VENN diagrams; in case of paracrine signaling proposed by the authors, there should be little overlap between 5-HT and HTR3A expression. In case of autocrine signaling (my point 1 above), most 5-HT expressing cells would also be positive for Htr3aEGFP.

4. A key experiment used by the authors to support their model is displayed in Extended Data Fig.5: neither proliferation nor apoptosis seem to be changed upon 5-HTP treatment, supporting the idea that serotonin affects the transition from bridge cells to chromaffin cells. However, given the overall low numbers of proliferative (and apoptotic) cells, proliferation (and ideally apoptosis) has to be quantified in TH+ cells in order to exclude an effect of serotonin on chromaffin cells. “n” might have to be increased to reach statistical significance.

5. Based on Fig. 1, most Htr3aEGFP+ cells co-express TH at E13.5. How can the authors explain, therefore, that in the vitro experiment described in Fig. 4, Htr3aEGFP+ but not TH is reduced? The authors should exclude that 5-HT influences transcription/expression of Htr3aEGFP+ rather than cell numbers. For the quantification of these data, I feel that counting numbers of marker-positive cells, rather than measuring the volume of 3D reconstructed images would be more accurate.

In line 236-237, it is written that the TH+ structures did not differ across all experimental conditions. This is surprising, as in vivo the adrenal glands are smaller following 5-HT treatment. It would be good to comment on this discrepancy here.

6. The statistical analysis should be described in the Materials and Methods section. Importantly, a standard error of mean (SEM) cannot be calculated with only three animals, this should be corrected and for those experiments, for which the numbers cannot be increased, the standard deviation has to be shown.

7. In Figure 2d, area measurements are shown. However, according to the text, measurements of cortical thickness have been performed. This needs to be clarified.

8. In Figure 6, a difference in the response between genders is shown. Why could that be? This should be discussed here or in the discussion.

Importantly, in this figure, the behavioral studies show barely any difference across the conditions. The authors should show here the single data points and assess statistical significance of the data.

9. The link between the study on HTR3A during chromaffin cell development and the tumorigenesis experiments presented in Figure 7 is poorly established. First, the association of HTR3Ahigh expression and tumorigenesis is purely correlative; the cell lines could obviously carry different mutations, etc, that may well explain their differential behavior independently of serotonin

signaling. To substantiate these data, the authors need to perform functional experiments (e.g. CRISPR knockout of Htr3a in the high expressing cells lines) in order to show that loss of tumor growth is indeed due to reduced expression of this receptor. Moreover, they should analyse the differentiation status of the neuroblastomas in order to strengthen their hypothesis that 5-HT treatment affects differentiation of bridge cell-like cells in the tumors. Do such cells exist in neuroblastoma? In the Discussion, the authors refer to cancer stem cell properties; are HTR3Ahigh cells "de-differentiated" (e.g. TH-negative) and exhibit cancer stem cell properties? Furthermore, they should comment on whether there is a correlation between reduced cell proliferation and differentiation status. Are differentiated cells less proliferative and, thus, less tumorigenic?

Finally, on line 311, the authors state that the reduced proliferation of the HTR3Ahigh expressing neuroblastoma following 5-HT treatment correlates with their in vivo mouse data. However, they showed before that there is no detectable difference in proliferation in neither SCPs, bridge and chromaffin cells following 5-HT treatment in vivo (Extended Data Figure 5). This discrepancy needs to be further clarified.

Minor points:

The main and supplementary figures should be numbered.

In general, in the figure legends, the arrowheads should be explained.

In Figure 3f, a statistical analysis should be performed, as done in all the other graphs shown in this figure.

In Figure 4c, the dot plot symbols in the different conditions are barely visible, the size of the graph needs to be increased.

In the Extended Data Figure S6b and S6d, the y-axis should be labelled.

In Figure 6a, catecholamine is misspelled, this should be corrected.

In Figure 6c, other names are used for the catecholamines tested than in the text of the paper. This should be corrected and misspellings should be taken care of.

On lane 286, Figure 6e is referenced wrongly, it should be 6c.

We would like to cordially thank the reviewers for very deep and detailed analysis of our study and for helping us to improve it. We carefully followed all the advices and tried to answer every comment by performing new experiments.

Reviewer #1 (Remarks to the Author):

This is a very interesting manuscript that describes how serotonin affects the generation of chromaffin cells during adrenal gland development. The authors discuss how adrenal gland development is mediated by a novel feedback loop based on paracrine signaling when serotonin and its receptor are released by early, mature chromaffin cells. The overall direction of this manuscript is very positive, however, there are several problems that need to be addressed and are outlined below. There are statements that need corrections and tone adjustments, and the authors should focus on their area of expertise, which is developmental biology.

We are thankful for the careful and insightful analysis of our work, and we agree with all questions that arose during the analysis of our study. As advised, we introduced numerous correction, run new experiments and adjusted the tone where it was requested.

The introduction is too long and at times, hard to follow. The authors introduced too much information without a clear, logical sequence, which is confusing to the reader.

We made the introduction shorter and more focused.

Line 80-81: The statements about the bladder should be deleted. The following sentence could be moved to the end of the introduction where the authors outline their hypothesis and objectives.

The statements about the bladder were deleted. And the introduction was restructured according to reviewer's advice.

Furthermore, the statements and expressions used makes it clear that the authors have limited knowledge in certain fields. For example, in line 96-97, the authors use the word "emergence." This word is almost never used when introducing or discussing information about tumors (specifically in this manuscript) and the authors should consider re-phrasing this. For example, when the authors describe neuroblastomas, I would recommend using the phrase "where these tumors are found."

We agree, and the word "emergence" is removed, when we talk about tumors.

Line 96: "Most importantly" should be changed to "Furthermore" in order to enhance readability and flow.

We rephrased the sentence about neuroblastoma and pheochromocytoma. "Most importantly" is now removed.

When discussing the organ of Zuckerkandl, the authors should understand that this is a site for paraganglioma, not pheochromocytoma.

We agree, and we corrected our phrasing throughout the manuscript.

Furthermore, neuroblastomas are mainly found in the adrenal gland, not the organ of Zuckerkandl. This is a weakness of the study since no pheochromocytoma cell lines were used when studying chromaffin cells (there are several pheochromocytoma cell lines that are readily available, such as MPC, PC12, MTT, and human pheo1). One of these cell lines will need to be added in present experiments and approaches.

We introduced the requested corrections. We did not find the pheochromocytoma lines, which reliably express *Htr3a/Htr3b*, and we assumed that our mechanism is not in place in those pheochromocytoma cell lines (although it might be relevant to some specific subtypes of *in vivo* pheochromocytoma tumors). However, we found several *Htr3a/Htr3b* expressing neuroblastoma lines, and we were able to include them in our study.
Besides, we are convinced that conclusions based on experiments with only one pheochromocytoma cell line would be inherently biased. Given the heterogeneity of pheochromocytoma genetic backgrounds (Dahia PLM. Nat Rev Cancer. 2014, 14(2):108-19. doi: 10.1038/nrc3648.) and various origin of the pheochromocytoma models available (different species; some artificially immortalized), we cannot cover such heterogeneity and draw a scientifically sound conclusion, especially given an issue with *Htr3a* expression.

Line 109: “the main neuroendocrine adrenergic organ” should be changed to “the main neuroendocrine organ” to reduce over-wording.

This is corrected now.

Results/discussion:

Line 149-155: The statement related to catecholamine production and PNMT must also be supported by the results related to tyrosine hydroxylase (see extended Fig. 3a-d).

We thank the reviewer for this notion. We now show the co-localization of TH and *Pnmt* mRNA at E15.5 in mouse adrenal glands. Please see Supplementary Fig. 4 c. and the related part of the manuscript

“Moreover, at E14.5, a subset of TH+ chromaffin cells in AM undergoes further functional specialization as shown by the onset of expression of Pnmt gene encoding phenylethanolamine N-methyltransferase (PNMT) (Supplementary Fig. 4b), the enzyme responsible for converting noradrenaline to adrenaline.”

Furthermore, the statement related to the release of catecholamines and serotonin (lines 154-155) may not be accurate. The presence of an enzyme does not guarantee that, for example, catecholamines will be released.

We agree with reviewer concerns, and to address this issue we performed additional experiments to confirm the serotonin release from the embryonic adrenals. Adrenal

glands were dissected from E14.5 fetuses of control and 5HTP treated females and incubated for 1 h in culture medium, then serotonin was measured in tissue homogenates and medium. Fetal kidneys were used as control. Serotonin release from adrenals greatly exceeds the release from kidney. Thus, the adrenal glands release serotonin from $5HT^+$ cells on top of what is possible as a carryover of serotonin from the remaining blood. Moreover, prenatal exposure to 5HTP significantly increased the release of 5HT by fetal adrenal glands. These new results were included into Fig. 2c.

Furthermore, the release of catecholamines from fetal adrenal glands was demonstrated earlier (N. S. Bondarenko, L. K. Dilmukhametova, A. Yu. Kurina, A. R. Murtazina, A. Ya. Sapronova, A. P. Sysoeva, and M. V. Ugrumov. Plasticity of central and peripheral sources of noradrenaline in rats during ontogenesis. Biochemistry (Moscow) volume 82, pages 373–379 (2017)).

As the chromaffin cells (and very rare “bridge” cells) are the only cells in the glands, which contain serotonin, we concluded that the release must be real.

We improved the text of the revised manuscript:

“Thus, chromaffin cells in mice are capable of releasing both 5HT and catecholamines during embryonic development and later during postnatal life[26-30] *being involved into a new system of cell number control, which we specify below.”*

and

“As expected, administration of 5HTP to pregnant females led to a significant increase of 5HT concentration in the placenta and trunks of E14.5 embryos (Fig. 2b), and caused enhanced release of 5HT by the fetal adrenal glands (Fig. 2c), as measured by HPLC. Thus, the administration of 5HTP to pregnant rodent females causes a stable and physiological increase of 5HT concentration in embryos, as it was previously shown for other tissues including the uterus [32, 33]*”*

Fig. 3f: Sympathetic ganglia is a very broad term and the authors should clarify which sympathetic ganglia they are talking about, and change the figure descriptions accordingly. What is the difference between sympathetic ganglia and mesenteric ganglion (line 231)?

We agree and we clarified the exact type of sympathetic ganglia in the text, figures and figure legends. Talking about sympathetic ganglia, we referred to the sympathetic chain ganglia (SCG).

“To control for the systemic effect, we checked the cells in the sympathetic chain ganglia (SCG), which are directly derived from the migratory neural crest cells and do not transition through a “bridge” stage[6, 35]*. Thus, SCG served as an internal control for the changes in size of chromaffin organs. 5HTP administration did not influence the size or cellular composition of SCG (Supplementary Fig. 5). Therefore, the reduction of chromaffin organs is specific and occurs at the expense of chromaffin cells.”*

In another example in the Results section we say:

"The SCG (representing a control tissue with different genesis) showed the same numbers of TH+ sympathoblasts in all groups (Fig. 3d)"

The mesenteric ganglion is another type of sympathetic ganglia that belongs to paravertebral ganglia that are positioned more ventrally as compared to the ganglia of sympathetic chain and have different innervation targets. We kept mentioning the mesenteric ganglion in the Fig 3, as it appears next to ZO on the sections, and we had to discriminate between those sympatho-adrenal structures.

What is the likely explanation of differences between the AM and ZO experiments shown in Figure 3? In other words, why did 5HT significantly changed early chromaffin cells in the AM but not in the ZO? I get the feeling that the authors circumvent explanations of "complicated" results and only focus on data that is nicely obtained from their experiments (e.g. see my comments related to catecholamines and anxiety tests in animals). Furthermore, the number of early chromaffin cells is increased in figure 3 (cartoon - OZ).

We agree with the reviewer and we performed two major experiments to understand the differences between the AM and ZO, which could explain the phenotype.

1. We performed a single cell transcriptomics analysis based on Smart-seq2, the deepest and most expensive method of sequencing, to compare the differences in lineage trajectories, differentiation, cell populations in control and in treated conditions separately in ZO and AM. This major experiment showed no critical difference between AM and ZO in control and in 5HTP treatment when we did a very strict analysis including compositional defects and velocity of differentiation (using our experiences from Furlan et al., Science 2017, La Manno et al., Science 2018 and Soldatov et al., Science 2019). We did not show multiple statistical tests that delivered negative results due to their negative nature. Even our high competence in bioinformatics analysis of single cell data (please see the aforementioned papers for confirmation) did not help us to understand the difference between ZO and AM phenotype under 5HTP treatment. The differentiation trajectory and the structure of all populations, including SCPs, bridge and chromaffin cells remained the same in ZO and AM. At the same time, the comparison of cells isolated from 5HTP-treated and control AMs and ZOs from several embryos revealed significant differences in the expression of gene *Cwc22*, responsible for the regulation of splicing in 5HTP-treated vs control group, indistinctively of ZO and AM. The highest expression of *Cwc22* among other cell types was found in "bridge" cells. Consequently, we detected the significant changes in differential splicing of individual genes responsible for progression though the cell cycle (*Apobec3* and *Cenpa.AS2*). Moreover, we detected differential splicing of several long non-coding RNAs (*Uph*, *Uph.AS2*, *Uph.AS3*, *Uph.AS4*) (Fig. 5e) controlling the expression of *Hand2*, transcription factor responsible for differentiation of cells towards catecholamine synthesis phenotype. Therefore, deep analysis of individual trasncriptomes allowed us to gain important insights into the possible mechanism of 5HT action in chromaffin cell differentiation process, i.e cell cycle control in "bridge" cells, although it did not explain the phenotype differences between AM and ZO. Please, see new Figure 5.

2. To analyze the possible changes in cell cycle progression (reflected also in single-cell transcriptomic data), we performed another major experiment (hypothesizing that the length of cell cycle, but not the general differentiation steps might be responsible for the difference). For this, we labelled the developing AM and ZO in control and treatment with two sequential pulses of different nucleotides to measure the cell cycle properties. The results clearly showed that indeed, in AM the "bridge" cells had prolonged cell cycle, which leads to the smaller pool of progenitor cells generated at a given time, and consequently to a smaller number of early and mature chromaffin cells. Please, see new Figure 4.
However, in ZO the mechanism of cell number reduction appeared to be different. ZO's SCPs, but not "bridge" cells show prolonged cell cycle. Please, see new Supplementary Fig. 6. The presence of adrenal cortex and signals from the local tissue might influence the AM but not the ZO, thus delaying the cell cycle in "bridge" cells only in AM populations.

3. We added the following description of the results to the revised manuscript:

"Contrary to this situation in AM, in E12.5 ZO, we observed lower proportion and reduced absolute numbers of SOX10+ SCPs incorporating EdU only in a treatment group (Supplementary Fig. 6c), which suggests another mechanism of cell number control or delayed dynamics of the 5HT effects in ZO."

We also updated the cartoon in Figure 3 to show the decrease of AM and ZO in size at the expense of mature chromaffin cells. Please check the new panel Fig. 3e.

Line 284-286 should indicate 6c, not 6e.

This is corrected.

The results are expressed as ng/organ, however, the weight of the adrenal glands differs so this data is incorrect. It should be expressed per protein (e.g. mg) or per weight (e.g. ng).

We agree with the reviewer and we added the data expressed in ng/organ in addition to the previously shown data. Please see new Figure 7c and Supplementary Fig. 9c. The absence of significant difference between control and treated groups in such representation demonstrates the normal functional activity of chromaffin cells. The results are expressed in ng/organ in order to demonstrate a decrease in the catecholamine content in the organ despite the unchanged synthetic capacity of the adrenal gland, i.e. catecholamine synthesis per cell does not change, but the number of cells decreases in the experimental group, thus, leading to the overall reduction of catecholamine supply in the organism.

The plasma data is not useful and does not correlate with the tissue data – what did the authors want to say here? Is there a meaningful physiological impact? If there is an effect of serotonin, differences between males and females, differences between organs (although, these were not measured correctly) and differences in plasma would be expected, however, there is no consistent trend and the results are not convincing.

We apologize for the inconvenient data presentation in the previous version. In the revised manuscript, we present the data as violin plots, where the reviewer can clearly see the distribution of the individual data points. Please see new Figure 7c and Supplementary Fig. 9c. In the revised version, one can see that in females the reduction of adrenal gland weight correlates with significantly reduced amount of adrenaline, noradrenaline and dopamine expressed in ng/organ as well as reduced concentration of adrenaline and noradrenaline in plasma.
In males, the reduction of adrenal gland weight correlates with the significant reduction on adrenaline (the major catecholamine with the highest concentration in blood as compared in noradrenaline and dopamine) expressed in ng/organ. Please, see the new figure Figure 7 panels a-c.
We made the description of correlation between adrenal gland size and catecholamines clear in the revised version by introducing the following text:

“The total body weight of P75 animals was similar in treated and control groups (Supplementary Fig. 9a-b), whereas the weight of adrenal glands was significantly reduced in males and females from the 5HTP-treated group (Fig. 7b). In embryonically-treated adult females, this reduction of adrenals correlated with significantly lower amounts of adrenaline, noradrenaline, and dopamine in the adrenal glands, and lower adrenaline in blood plasma (Fig. 7c). In males from the 5HTP-treated group, smaller adrenal glands contained respectively less adrenaline, but the levels of noradrenaline and dopamine did not show any significant difference (Fig. 7c).”

Moreover, the data between noradrenaline and normetanephrine do not match. This is confusing.

The previous version of the manuscript lacked the explanation of the purpose of catecholamines metabolites measurement. Metanephrine and normetanephrine are metabolites of adrenaline and noradrenaline and show the rate of catecholamine metabolism. We measured these compounds to ensure that the observed reduction of adrenaline and noradrenaline was due to the reduced number of catecholamine producing chromaffin cells and not due to the increased catabolism of the major catecholamines. Since the concentration of metanephrine was not changed in 5HTP treated vs control males and females, the observed reduction of adrenaline is explained by reduced number of chromaffin cells and not the enhanced catabolism.
At the same time, the concentration of normetanephrine is significantly higher in 5HTP treated males and can be explained by the enhanced catabolism of noradrenaline. This might be responsible for the downward trend observed in plasma noradrenaline in males. Males can have different metabolic ratios from the females when it comes to catecholamines, as they have different response to stress.

We introduced the following text:

“To ensure that the observed reduction of adrenaline in males and the reduction of adrenaline, noradrenaline and dopamine in females were not associated with accelerated catabolism, we measured catecholamine metabolites: metanephrine and normetanephrine (Supplementary Fig. 9c). These measurements confirmed that the decrease in adrenaline supply from the adrenal glands in adult animals from the 5HTP-treated group was not due to the acceleration of adrenaline catabolism

(Supplementary Fig. 9c). On the other hand, the concentration of normetanephrine was significantly higher in 5HTP-treated males, which might indicate the enhanced catabolism of noradrenaline. As the elevation of normetanephrine is not observed in females, this might signify the sexual dimorphism in endocrine metabolic systems and might explain the behavioral differences observed in males and females."

Finally, to be careful and avoid strong conclusions related to these data, we added the following discalimer into the main text:

"Because the system of catecholamines and their metabolism is complex and stretches beyond the production of adrenaline and noradrenaline in chromaffin organs, the observed differences in males and females, as well as the only partial correlations with behavioral data might be due to other systemic regulatory mechanisms, which are not covered by this study."

I am very confused with the results from the pharmacological study. In the conclusion, the authors state: "These results are in line with the in vivo measurements and indicated the persistant reduction of stress hormones which are released from the chromaffin cells in animals prenatally exposed to the elevated 5-HT level." This is not true. Furthermore, the sentence is very difficult to understand and reflects practically no specific data. In males, differences have not been seen in plasma adrenaline, only mild decreases in noradrenaline, no supporting data for normetanephrine, and the behavioral studies on 6e-g oppose the catecholamine data.

1.In the Figure 7c it is evident that there is a reduction of adrenaline in 5HTP treated males and females measured as ng/organ (adrenal gland). Moreover, in females the significant reduction is observed for noradrenaline and dopamine measured in ng/organ. At the same time, we admit that we do not observe the significant reduction of adrenaline and noradrenaline measured as ng/ml plasma in males, as we observe in females. The differences in observed phenotype can be explained by sexual dimorphism of catecholamine catabolism in plasma, as we know that the males have a stronger reaction to stress as compared to females.
2. The concentration of normetanephrine is significantly higher in 5HTP treated males and can indicate the enhanced catabolism of noradrenaline. This might be responsible for the downward trend observed in plasma noradrenaline in males.
3. We substantially revised and improved the behavioral tests. We now employed two additional test that were designed to assess the aggressive behavior. Those test are done exclusively on males and our results show a clear cut behavioral changes in 5HTP prenatally treated males. In the resident-intruder test and the foot shock induced aggression test, 5HTP prenatally treated males showed no aggressive behavior in comparison with control males. This sharp behavioral changes are direct consequences of the reduced overall adrenaline supply for the organism measured in ng/organ.
4. When it comes to the test performed previously, which reviewer is interpreting as contradicting, we do not see them as such, because they test for adaptive and flexible behavioral patterns like exploration of the unknown territory, coping with stress and avoidance. Novelty induced hypophagia and extrapolation escape task show that 5HTP prenatally treated males possess such characteristics, as they spend significantly less time to take food and avoid uncomfortable (being in the water) situation and we believe, it is a direct consequence of reduced adrenaline supply from

the smaller adrenal glands. These data are now described in light of the reactive and proactive coping strategies, descried in a greater detail in the answer for reviewer's next question.

5. To finally accommodate the reviewer's comment, we changed the text in the revised version of the manuscript. Now the statement reads in the following way:

"Moreover, the experimental animals turned out to be more adaptive and flexible, demonstrating less anxiety and reduced stress-induced behavior (Supplementary Fig. 9d). These results are in line with in vivo measurements of catecholamines in adult mice revealing lower levels of adrenaline supplied by smaller adrenal glands (evident when measured as ng/organ, Fig. 7c) in animals prenatally exposed to the elevated 5HT levels."

Males showed changes (compared to females) in the hypophagia test and escape task, however, these differences were not discussed or explained. Experimental animals quickly found ways to escape from water, however, this group had lower adrenaline and noradrenaline in either the adrenal gland or plasma. Your data does not fit. Are the authors trying to say that lower catecholamines reflect better and quicker escape plans, or the ability of an animal to free itself to obtain food, or something else? This is extremely confusing and needs a very good explanation. This also applies to the abstract (line 48) where the statements do not add value or help educate the reader. Figure 6f and 6g could show data for females first to keep consistent with figure 6c.

We thank the reviewer for a very insightful comment and we were puzzled ourselves with this situation. To resolve the conundrum, we designed additional behavioral tests aiming to discriminate between impulsive and aggressive behavior vs more plastic and adaptive strategies, utilizing the concepts of so called "coping strategies". The concept of coping strategies is well recognized and is used for the description of the animal behavior in laboratory animals and in the wild populations of rodents. Please refer to the following publications [1, 2]. According to this theory, aggressive animals with higher levels of catecholomines typically express a more **proactive** type of behavioral response demonstrating rigid and impulsive reactions which results, for example, in their tendency to defend their home territory. At the same time, non-aggressive **reactive** animals are rather flexible, cautious, and open to the external cues, which can assist in variable or unpredictable environments. Both of these behavioral characteristics (proactive and reactive) are dependent on physiological and neuroendocrine status in rodents. It has been demonstrated that individuals with low sympathetic reactivity expressed reactive behavior.

Following this line of thinking, we have performed additional behavioral test to analyze the degree of aggressive, proactive behavior in prenatally 5HTP treated animals. Please see new results in Fig. 7 d, f. According to our new data, the rats from 5HTP treated group were much less aggressive in comparison to control group, as they expressed no attack behavior in the resident-intruder test and in the foot shock induced aggression test (Fig. 7d). Therefore, prenatal exposure to increased serotonin leads to reduced size of adrenal gland, which manifests in the lower levels of adrenaline and changes the behavior of the animals towards a reactive type.

In line with this, the previously presented experiments can be explained by the fact that prenatally 5HTP treated animals are more flexible in decision making, more curios and express less fear of the unknown environment. In elevated plus-maze test prenatally 5HTP treated females spend significantly longer time in the open arms of the maze. In novelty induced hypophagia males needed significantly less time to take food. In extrapolation escape task the group of animals that needed shorter time for diving and escaping was proportionally higher. All these results (even though males and females showed sexually dimorphic performance in tests) strongly suggest that 5HTP-treated animals demonstrate more flexible and adaptive behavior.

Finally, to test if adrenal medulla size correlates with the coping strategies in natural conditions, we performed an expedition to Siberia and studied the wild rodent population. Indeed, the concept of coping strategies is often applied to the analysis of population dynamics in wild rodents. It is well known that colonies of wild voles have “cycling” structure with the periods of colony size growth changing with the periods of extensive migration aiming to occupy new territories and reduce the pressure of overpopulations. From previous studies we know that coping strategies of the animals that emigrate and animals that reside in the original areal are different. To test how this corresponds to the size of adrenal medullae, we collected adrenal glands from the resident and migrant wild red-backed voles during the year of peak migration (2020). The comparison of relative adrenal medulla size between the animals in “resident” and “migrant” groups showed that the “migrant” animals had significantly smaller medullas in adrenal glands.

This observation ties together the results of experiments presented in the revised manuscript and places the observed connection between elevated levels of maternal serotonin and the size of adrenals in progeny into the new ecological perspective. Elevated levels of serotonin in mothers lead to the reduction of chromaffin cells in progeny, which is reflected in hormonal balance of adult progeny and changes in their behavioral patterns. Furthermore, we report similar behavioral phenotype in progeny after the simulated mild stress in pregnant laboratory animals (new Fig. 6 e,f). The increased population density of wild rodents is associated with increased levels of stress, which leads to the increase in 5HT (as known from the literature and our own new data). This might, in turn, result in the birth of a generation, which is capable of exploring new territories due to their altered hormonal balance and more adaptive behavior. Therefore, it is reasonable to speculate that observed 5HT-HTR3A link might be acting as a way of prenatal programing of offspring with more flexible and adaptive coping strategy to allow for colony segregation on “residents” and “migrants” necessary to maintenance of the appropriate colony size.

The data related to neuroblastoma cells is interesting and relevant, however, the authors describe that their results support the idea that neuroblastomas might resemble bridge progenitor cells. In the experiments with AM, 5HTP decreases early chromaffin cells but not bridge cells (although, the cell number related to bridge cells in figure 3 is in question – did they decrease?), thus, your conclusion may not fit. Perhaps early chromaffin cells can be affected instead of bridge cells? If only proliferation is decreased, what would the effect of N-methylquipazine be on tumor growth in NSG mice?

1. We understand the reviewer's concern, and indeed one of the key ideas is that "bridge" cells might be relevant to neuroblastoma and neuroblastoma cell of origin. During this revision, several strong manuscripts were published that strongly support these ideas based on the single cell transcriptomics data comparisons. For instance, please see Jansky et al. Nat Genetics 2021. The Jansky et. al. team showed that neuroblastoma tumors contain malignant subpopulations, which are transcriptionally most similar to the human embryonic "bridge" cells.

We mention this is the text of revised Introduction and Discussion:

"Moreover, another recent study suggested the involvement of "bridge" chromaffin progenitors in neuroblastoma initiation [9, 10]*."*

"The comparison of human $HTR3A^{high}$ *and* $HTR3A^{low}$ *neuroblastoma cell lines revealed that cell lines with* $HTR3A^{high}$ *expression level have higher tumor-initiating potential, as those cell lines appeared tumorigenic in a mouse xenograft model system and formed significantly more spheres in vitro. These are key characteristics of cancer stem cells, and this observation may point to the previously unrecognized cell states responsible for tumor aggressiveness, in line with recent findings of similarity of genetic profiles of* $HTR3A^{+}$ *"bridge" cells and neuroblastoma subpopulations* [8-10]*"*

2. Next, although the chromaffin cells reduce their numbers after 5HTP treatment, this is due to the upstream defect in the "bridge" cells. According to our new data that we added during this revision, "bridge" cells slow down their cell cycle, which leads to the deficit of precursor cells ("bridge" cell numbers are significantly smaller in 5HTP treated group in AM as you can see in the new figure 4b) and subsequent lack of chromaffin cells, which barely proliferate on their own and do not enter apoptosis. Please find new data in revised Figure 4.

3. Also, we have now removed the referred part of the manuscript (Line 313-315 in the previous version), as we agree with the reviewer that such interpretation of HTR3A expression in neuroblastoma would still be oversimplifying. Tumor cells differ from healthy cells including developing progenitors. The way how tumor cells might differ in terms of interpreting *Htr3a*-mediated signal requires full scale project investigation.

To be careful, we mentioned this in the text of the Discussion:

"In line with the in vivo cell cycle progression experiments, we managed to inhibit the proliferation rate of $HTR3A^{high}$ *neuroblastoma cells with a specific HTR3A agonist, which might be developed into a potential therapeutic strategy, especially in a combination with differentiation-inducing drugs* [63-65]*. Still, it might be wise to keep in mind the potential difference between tumor and healthy* $HTR3A^{+}$ *"bridge" cells, as the tumor cells might have additional, unpredictable effects following from HTR3A activation."*

Next, our further experiments with dual thymidine analogs labeling of cell cycle show that only "bridge" cells ($Htr3a^{EGFP+}TH^{-}$ cells in AM) are affected by 5HTP treatment as this cell population is the only one that shows slowing down of the cell cycle (Fig. 4b,c). In line with these results, additional sphere assay experiments show that activation of HTR3A receptor does not compromise the stem cell-like state of

neuroblastoma HTR3A^{high} cells, but only reduces their proliferation (Fig. 6i, Supplementary Fig. 8). The pre-treatment with HTR3A agonist N-methylquipazine dimaleate (NMQ) for 5 days did not reduce sphere forming capacity of both HTR3A^{high} and HTR3A^{low} cell lines (Fig. 6i). Thus, enhanced HTR3A activity over 5 days results in marked inhibition of proliferation (as evident also from MTT results, Fig. 6d,e) but does not induce differentiation nor apoptosis of neuroblastoma cells. Although we have not tested the effects of NMQ on tumor growth *in vivo*, these results suggest that treatment with HTR3A agonists as monotherapy would very likely fail to improve neuroblastoma cure rates, but these agents show potential to be utilized in combination therapies, possibly with differentiation-inducing drugs.

The statement in lines 318 – 322 is not a useful way to begin a discussion. The authors should first directly state the best outcomes and results of the present study. Starting out with the stress response and avoidance behavior is not very tactical because this is a weak part of the study. You presented excellent results in other parts of this study, so the authors should focus on these. The same goes for the "migration" data – you did not study it here so it should not be emphasized.

We are grateful for the advice and we now revised the discussion following this logic. We removed the confusing first sentence.

If 5HT is increased in pregnant women, the authors should state its clinical relevance. As described on page 16, there are clinically relevant situations and the authors should focus on which ones are most important. Mild stress, moderate hypoxia, and vitamins have not been proven to be of major importance in adrenal gland-related tumors. The authors should attempt to treat animals with vitamins or exposed them to stressful conditions and see if they get similar results to their experiments. This would support their data and it would greatly contribute to the pathophysiology of adrenal tumors.

We thank the reviewer for pointing this direction. We took this advice seriously and conducted the experiments that show that pregnant mice exposed to mild stress conditions (1 h restrain) during the specific time window of adrenal gland development have similar to increased serotonin effect on the development of adrenal glands. The adrenal glands of the offspring of stressed females are smaller than in control group. This phenotype is highly likely to be mediated by the increased levels of serotonin in placenta and in fetuses in stressed females according to our new experimental measurements (Fig.2). Please see the new results in revised Figure 7. Although the pregnant women often take SSRIs to treat pregnancy associated depression, this condition is very complex, and we would like to avoid speculating too much about this in our rather developmental and mechanistic study.

The statement in lines 386-287 is of interest but lacks the supporting data. You would need to perform the appropriate experiments, which could be feasible (line 392-393).

To support our hypothesis, we have performed additional experiments with HTR3A agonists, N-methylquipazine dimaleate (NMQ) and SR57277. Both HTR3A agonists markedly inhibited proliferation of HTR3A^{high} neuroblastoma cells in a dose-response manner (Fig. 6d,e). These results clearly support the perspective that the 5HT-HTR3A axis is a mechanism preventing excessive proliferation and modulation of HTR3A

activity in HTR3A^{high} neuroblastomas might be potentially exploited in therapeutic strategies.

I am not sure whether the statements in line 396-405 support your data. It seemed to be contradictory when I read it.

We now revised this part of the discussion making sure that we present it as a hypothesis and stating that we do not have direct data supporting it

"Hypothetically, the molecular mechanisms controlling the size of chromaffin tissues are important for natural and artificial selection. Although we do not provide the direct data supporting this idea, the low aggressiveness, changes in 5HT synthesis and degradation, and reduction of chromaffin organs were previously reported as a part of the so-called "domestication syndrome", observed in a number of domesticated species [21, 22]*. In line with these domestication-associated behavioral patterns, our experimental rodents subjected to 5HT-driven reduction of adrenals showed less aggressive responses and altered levels of catecholamines."*

Were the histopathological sections of the adrenal glands the same size? Note that adrenal gland (medulla) is changing the size. How were all of the results calculated?

1. We agree with reviewer's concerns, and we were very careful with such analysis. First of all, we checked the proportion of medullary tissue in the whole adrenal gland. Secondly, we of course also measured the absolute surface area of the medulla on multiple sections of the analyzed glands. Thirdly, the major observation of adrenal gland size difference in control vs treatment comes from the fine 3D reconstruction of micro CT scans (updated figure 2 d-g).

2. To be more precise, the measurement of adrenal cortex and medulla was done on 2D scans of multiple adrenal gland sections. The areas of the sections of the entire adrenal glands did not show the reliable reduction in the 5HTP-treated group due to the fluctuations of cortical structures. Indeed, the significant reduction of the medulla area becomes compensated by the variability of the cortex area and does not result in the significant reduction of the whole adrenal gland area. Despite the whole area of adrenal glands did not show significance in decrease due to such variability, the medulla area was significantly smaller in the 5HTP-treated group. In line with this, when we compared the 3D volumes of the entire adrenal glands from treated and control groups, we found a significant difference in 5HTP-treated group, where the entire gland appeared smaller. Thus, for comparison of the total gland volume, the 3D analysis turned out to be more sensitive as expected.

3. Also, in case of the following experiments we calculated the ratio between medulla and cortex to normalize the measurement for the high variability of adrenal glands size within one organism (left to right asymmetry) and between different organisms.

The detailed description of how results were calculated were added to the method section.

“Cell counts and area measurements were done manually using the Cell counter plugin and measurement functions of ImageJ (2.1.0/1.53c) software. The area of adrenal gland section was calculated by surrounding the area based on DAPI signal. The area of medulla was calculate based on TH+ signal within adrenal gland. The area of cortex was calculated by subtraction of adrenal medulla area from the area of the whole gland per individual section. 3 section per gland and 2 glands per embryo were evaluated.”

“The optical section with maximal external volume was selected for relative medulla size analysis. The area of central medulla and the whole adrenal were measured using ImageJ software. Relative medulla size represented by the ratio: (medulla area/total area) × 100%.”

The adrenal gland is not a major source of norepinephrine.

We carefully went through the literature to accommodate this concern and found this particular study from Prof. M.V. Ugrumov laboratory:
A. R. Murtazina, Y. O. Nikishina, N. S. Bondarenko, A. Ja. Sapronova, M. V. Ugrumov. Signal Molecules during the Organism Development: Central and Peripheral Sources of Noradrenaline in Rat Ontogenesis. *Doklady Biochemistry and Biophysics, 2016, Vol. 466, pp. 74–76. etc.*
This study states that the adrenal gland and organ of Zuckerkandl is the major source of embryonic norepinephrine. However, we understand that there are alternative opinions including the synaptic release and spillover of norepinephrine in other parts of peripheral nervous system. Thus, we toned down this entire part.

There are too many abbreviations, making it very difficult for readers to orient themselves when reading the text. I would recommend that the authors make a table with all of the abbreviations listed.

We are thankful for this comment, and we made the list of abbreviations, genes and proteins names in the revised manuscript.

Moreover, when you talk about TH+ cells in line 219 and figure 3f, do you mean both early chromaffin cells and mature chromaffin cells?

For the experiment described in the former line 219, we refer to both early and mature chromaffin cells collectivelly. We now made it clear in the revised manuscript:

” Administration of SR57227 to pregnant mice at E11.5 and E12.5 caused a 30.6% reduction (calculated between mean values of DMSO control and SR57227 groups) of early and mature TH+ chromaffin cells in AM at E13.5 (Fig.3d)”

We now clarified the exact markers of early chromaffin cells and mature chromaffin cells. Please see the example of the text:

“Therefore, the Htr3a^{EGFP} mouse strain can be used to trace the transition from the intermediate “bridge” cell population to the chromaffin cells by assessing the

proportions of TH$^{—}$/Htr3a^{EGFP+} "bridge" cells, TH^{+}/Htr3a^{EGFP+} early chromaffin cells and TH^{+}/Htr3a$^{EGFP—}$ mature chromaffin cells"

This distinction was clearly possible when we analysed the section of adrenal glands from *Htr3a^{EGFP}* mice.

As mentioned above – what are the differences between sympathetic ganglia and mesenteric ganglion? There are no differences to my best knowledge. If there are no differences, why are they listed separately?

The reviewer is correct that mesenteric ganglion belongs to the broad company of sympathetic ganglia. We now clarified that when we referred to sympathetic ganglia we referred to sympathetic chain ganglia in particular. To avoid any confusion, in the revised version of the manuscript, we changed "sympathetic ganglia" to "sympathetic chain ganglia". Mesenteric ganglion differs from the sympathetic chain ganglia by the location and innervation targets. We outlined the mesenteric ganglion because it appears on the sections with organ of Zuckerkandl.

Why is figure number 3 placed before figure number 2 in the text?

We fixed the order of the figures.

Minor:

Misspelling: catecholamine – in extended figure 1c.

We fixed this.

Delete pediatric in line 385.

We fixed this.

Decide whether you want to use sympatho-adrenal or sympatho-adrenal and stay consistent.

We made it consistent.

Line 123: put the abbreviation for TH here.

This is fixed.

Line 286: Figure 6e does not describe catecholamine levels.

This is fixed.

Reviewer #2 (Remarks to the Author):

This paper presents interesting insight to the formation and functionality of chromaffin organs. The manuscript is based on previous work describing the transcriptional landscape and heightened expression of the serotonin receptor HTR3A in an intermediate “bridging cell” which links the developmental transition from schwann cell precursor to chromaffin cell. Overall the work is well presented and thorough. Whilst there are some inconsistencies between the mechanistic findings and neuroblastoma the work should be of wide interest.

We are grateful for reviewer’s precious time and careful assessment of our study, and especially for the useful advices that undoubtedly helped us to improve our work.

Major comments:
1. The authors suggest a time-window in which 5-HT may play a role on the bridge cells to regulate chromaffin development. This is based on expression of EGFP driven from the Htr3a promoter. As the GFP is stable for some time, this work would be strengthened by repeating with antibodies and RNA expression analysis. Even better would be to treat pregnant mice with excessive 5-HTP at different ages to test this.

We agree, and now we performed two new experiments according to the reviewer recommendation:

1.The RNA in situ hybridization for *Htr3a* receptor gene was performed on the sections of adrenal glands from E13.5 control embryos exposed to 5HTP during E11.5-E12.5. In both cases, our calculations show that *Htr3a* RNA is strongly expressed mainly by *Htr3a*$^{EGFP+}$ cells and in a small subset of Sox10^{+} cells that differentiate towards “bridge” fate as predicted by single cell data. Please check new panel Supplementary Fig.5 a, which shows consistency of RNA and *Htr3a*^{EGFP} signal in “bridge” cells.

Unfortunately, we found no reliable antibody against HTR3A, which also initially forced us to start using *Htr3a*^{EGFP} mouse model.

2. Following, the advice of the reviewer, we treated pregnant mouse and rat females with the precursor of serotonin 5HTP during the most active differentiation of “bridge” cells into chromaffin cells and outside of this productive time window. We observed a much smaller reduction of the adrenal medulla, when 5HTP was given to the pregnant females after the most active “bridge” cells differentiation. Please check the new figure 2h and the following description in the updated text:

“5HTP treatment of pregnant rats and mice during the active differentiation of “bridge” cells into chromaffin cell resulted in a long-lasting reduction of AM size in embryonic and postnatal life of both species. Administration of 5HTP outside of this critical time window led to a less pronounced effect (Fig. 2h). Thus, we were able to influence the size of the adrenal glands in rodent offspring through the elevation of 5HT levels in pregnant animals during a limited developmental time window corresponding to the peak of chromaffin cell generation.”

2. All of the histological analyses are performed in mice with rats used for many of the functional analyses. There needs to be at least some investigation in rat embryogenesis to provide confidence that the same developmental mechanisms are conserved across species.

We agree, and now we show histological analysis of rats (figure 2d-h) and functional analysis of mice (Figure 2 a-c, Figure 6e, f) in the revised version of the manuscript. We also performed exactly similar experiments on mice and rats showing that the observed phenotype is consistent across these species (Fig 2h). The results are discussed in more detail below:

"5HTP treatment of pregnant rats and mice during the active differentiation of "bridge" cells into chromaffin cell resulted in a long-lasting reduction of AM size in embryonic and postnatal life of both species. Administration of 5HTP outside of this critical time window led to a less pronounced effect (Fig. 2h). Thus, we were able to influence the size of the adrenal glands in rodent offspring through the elevation of 5HT levels in pregnant animals during a limited developmental time window corresponding to the peak of chromaffin cell generation."

Analysis of adrenal glands in rat embryos is limited to females yet males and females are included in the adult studies.

We are sorry for the unclear description of the results in the previous version and we confirm that the analysis of embryos was gender neutral as we used both males and females embryos. We apologize for any misunderstanding. When we write "5HTP treated females" we refer to pregnant females, which contain embryos of both sexes.

There are also inconsistencies between the number of rat embryos used for microCT and histology – n=4 and 3 for these embryonic studies is low considering entire litters could be used. The number if embryos used in Fig 3 also needs clarification. The title infers only one pregnant female was used with n=3 embryos.

For comparison based on microCT and immunohistochemistry, we used the whole rat litters, which contained 8 embryos in the control group and 9 embryos in the treatment group. 4 embryos from each litter were used for microCT; 4 and 5 embryos from control and treatment respectively were used in histological analysis. As this analysis in rats showed the significant difference, which we later reproduced in several litters of mice, we kept the original data.

The decrease in area by microCT in rat embryos also outweighs the reduction in size of the medulla.

We apologize for the unclear presentation of the results in the previous submission, and we would like to explain the differences in micro-CT-based **volume** measurements and **area** measurements on 2D sections. The volume changes as a cubic function, whereas the area changes as a square function. Therefore, the decrease in volume appears stronger and naturally outweighs the decrease in the measured area. We now introduced the clarifying subtitles into the Figure 2f,g.

3. It remains unclear how increased 5-HT leads to a lack of chromaffin cells in the medulla. EdU incorporation suggests no alteration in the proliferation of bridge cells, schwann cells or chromaffin cells, yet there are less cells overall. Without increased cell death, how is the overall cell number reduced, not just the proportion? Prior studies of chromaffin cell development have shown changes in cell cycle length and proliferative capacity which may be worth investigation. A 24 hr chase may be too long to uncover any changes in this experiment.?

We are very thankful the reviewer's insightful suggestion and we were inspired to perform a new major experiment to measurement the cell cycle progression in different cell populations building adrenal glands. We gave two pulses of thymidine analogs EdU and CldU at 4-hour intervals together with the 5HTP treatment to label the cells during S-phase of the cell cycle and to analyze the proportions of cells, which incorporated EdU only, CldU only, and both nucleotides. The difference in proportions of labelled cell reflected the changes in cell cycle progression. The results are summarized in the new figure 4.We started the treatment at E11.5 with one dose of 5HTP, and we gave the second dose of 5HTP and consecutive pulses of thymidine analogs at E12.5. We collected embryos in the evening of E12.5, therefore embryos analyzed for this experiment are different stage from embryos presented on Fig. 3 and Supplementary Fig. 5 (E13.5), and we expect to see slightly different numbers of cells.
The proportions of cells incorporated EdU only was lower and the proportion of cells which incorporated both EdU and CldU was higher among "bridge" cells in AM of embryos from 5-HTP treatment group. At the same time the number of proliferating cells (cells that incorporated at least one label) was not different among SCPs, "bridge" cells, early chromaffin cells and mature chromaffin cells. These results allowed us to conclude that "bridge" cells have prolonged cell cycle during our observation period under the treatment with 5HTP. Therefore, as reviewer correctly hypothesized, the change in cell cycle length results in less precursor cells being available for differentiation (please see significantly decreased cell numbers of "bridge" cells in addition to early chromaffin cells in AM at E12.5 according to our new data shown in figure 4b). This prolonged cell cycle of progenitors causes the downstream deficit of chromaffin cells and results in the reduced size of adrenal gland medulla. No changes in cell cycle progression was observed for other cell populations. It is also important to mention that there are negligible cells, which incorporate EdU or CldU among mature chromaffin cells, as they temporarily exit cells cycle at the beginning of maturation.

To make sure we did not miss any important difference between treated and control conditions, especially when it comes to the differentiation dynamics and the structure of transitory populations, we performed another unbiased experiment using single cell transcriptomics. Our single cell sequencing was based on Smart-seq2, the deepest and most expensive method of sequencing, and we aimed to compare the differences in lineage trajectories, differentiation, cell populations in control and in 5HTP-treated conditions. As a result, we revealed that the differentiation trajectory and the structure of all populations (identity and the proportions of cell types), including SCPs, "bridge" and chromaffin cells remained the same in treated and untreated conditions. At the same time, the comparison of cells isolated from 5HTP-treated and control AMs and ZOs from several embryos revealed significant differences in differential splicing of genes responsible for progression though the cell cycle (*Apobec3* and *Cenpa.AS2*). This change in differential splicing might be mediated by *Cwc22* gene, responsible for the regulation of splicing, which is differentially expressed in 5HTP-treated vs control group and has the highest expression in "bridge" cells among the other cell types.

Therefore, deep analysis of individual transcriptomes is in agreement with the observed 5HT-mediated changes in ”bridge” cells cell cycle progression. Please, see new Figure 5.

The schematics in Fig 3 tend to exaggerate the data a bit too much (in C bridge cells should be the same, while early chromaffin cells should not be increased in e).

We agree and we updated the scheme in Fig. 3 to better reflect the experimental observations.

If bridge cells fail to differentiate what happens to them.

Because of our new experiments (double thymidine analogues injection (Figure 4) and single cell transcriptomic analysis of cells from 5HTP-treated vs control embryos (Figure 5), we abandoned the version of differentiation failure in “bridge” cells. Instead, we detect the slower cell cycle and resulting slower (reduced) accumulation of the terminally differentiated chromaffin cells.

4. The link to neuroblastoma is not consistent. The models used are well characterized as neuroblast models so the finding that some have higher levels of Htr3a is interesting. What does this mean for their origin? Are these more precursors of SG, SCP, bridge cells?

We thank the reviewer for this important question.
1. During the revision time, several studies compared human embryonic sympatho-adrenal populations (SCPs, bridge, chromaffin and sympathetic) to the spectrum of neuroblastoma tumors. The results of such new comparisons predicted that some populations of malignant neuroblastoma cells show strongest similarity to embryonic “bridge” cells (please see Jansky et al., 2021 Nature Genetics and a perspective by Hermann Rohrer in the same issue for details). However, it does not necessarily mean that these neuroblastomas originate from “bridge” cells, and the malignant cells can simply re-enact the “bridge” genetic program for their benefits.
2. The cell lines that we used in this study, were also compared to the embryonic single cell datasets, and SH-SY5Y line with high *Htr3a* expression was found to be similar to sympatho-adrenal cluster of progenitors (Boeva and van Groeningen 2017 Nature Genetics).

Both of these arguments improve the connection between “bridge” cells, *Htr3a* and neuroblastoma. We introduced a new text into the manuscript:

“Moreover, another recent study suggested the involvement of “bridge” chromaffin progenitors in neuroblastoma initiation [9, 10]*.”*

“The comparison of human HTR3Ahigh and HTR3Alow neuroblastoma cell lines revealed that cell lines with HTR3Ahigh expression level have higher tumor-initiating potential, as those cell lines appeared tumorigenic in a mouse xenograft model system and formed significantly more spheres in vitro. These are key characteristics of cancer stem cells, and this observation may point to the previously unrecognized cell states responsible for tumor aggressiveness, in line with recent findings of similarity of genetic profiles of HTR3A^{+} “bridge” cells and neuroblastoma subpopulations[8-10]*.”*

Activation of HTR3A had no effect on bridge cell proliferation in vivo, yet activation in SH-SY5Y reduced their proliferation, which is inconsistent. Does this make them differentiate? This data is taken to infer that the 5-HT feedback might prevent excessive organ growth and tumour formation. However, it could also be possible that increased 5-HT blocks differentiation, driving tumour formation.

> We agree that the differences in HTR3A expression among neuroblastoma cell lines are intriguing, especially as HTR3A^{high} expression associated with more aggressive neuroblastoma phenotypes. Moreover, strengthening the biological significance of our findings, we have now included two more neuroblastoma cell lines, CHLA-15 and CHLA-20, and performed most of the experiments again with the whole panel of six neuroblastoma cell lines (Fig. 6; further details in the letter). While it would be speculative to discuss the cell-of-origin of HTR3A^{high} neuroblastoma cell lines, all HTR3A^{high} cell lines were sensitive to HTR3A-specific agonists, which inhibited proliferation of these neuroblastoma cells (Fig. 6d,e). This is in agreement with the new results of our cell cycle analyses using dual labeling by thymidine analogs which showed that only "bridge" cells (Htr3a^{EGFP+}TH^{-} cells) are affected by 5HTP treatment as this cell population was the only one that shows a slowdown of the cell cycle (Fig. 4b,c) without an induction of apoptosis (Fig. 4e). In line with these experiments, HTR3A agonists also did not induce apoptosis in neuroblastoma cells (Supplementary Fig. 8). To investigate the possibility that the excessive stimulation of HTR3A may block differentiation of neuroblastoma cells, we performed new experiments utilising limiting dilution sphere formation assay as a well-accepted *in vitro* test of undifferentiated/tumor-initiating cells. However, our results clearly show that HTR3A agonists do not affect differentiation of HTR3A^{high} neuroblastoma cell lines, as N-methylquipazine (NMQ)-pretreated cells maintained similar sphere forming capacity as vehicle-pretreated controls (Fig. 6i). Together, our data suggest that 5HT-HTR3A axis limits proliferation of both "bridge" cells and HTR3A^{high} neuroblastoma cells by interfering with the cell cycle progression. We conclude 5HT-dependent HTR3A activity in "bridge" cells explain the observed changes in cell numbers and overall size of the developing adrenal medulla and we hypothesize that disruption of 5HT-HTR3A axis may potentially lead to tumor-permissive conditions.

One notable observation in your work that is not discussed, and should be, is the increase in Normetanephrine (incorrectly spelt in Figure) in male 5-HTP treated rats. This is a marker of pheochormocytoma in humans, and should be discussed.

> We agree the reviewer, and now we discuss this result in the revised manuscript:
>
> *"To ensure that the observed reduction of adrenaline in males and reduction of adrenaline, noradrenaline and dopamine in females were not associated with accelerated catabolism, we measured catecholamine metabolites metanephrine and normetanephrine (Supplementary Fig. 9c). These measurements confirmed that the decrease in adrenaline supply from adrenal gland in adult animals in the 5HTP-treated group was not due to an acceleration of adrenaline catabolism (Supplementary Fig. 9c). On the other hand, the concentration of normetanephrin was significantly higher in 5HTP-treated males, which might indicate the enhanced catabolism of noradrenaline, and is typically observed in major pheochromocytoma subtypes in human [46, 47]."*

Minor suggestions:

1. The last sentence of the abstract is ambiguous.

   These last sentence of the abstract is removed.

2. More information is needed for the expression patterns used to define chromaffin cells (immature or mature), bridge cells and Schwann cells in the single cell data

   During the revision we performed new extensive single cell RNA sequencing and the new data is presented in the new Fig.5 with the characterization of genes used to annotate the clusters (new Fig. 5). The cell clusters were consistent with previously published by Furlan et. al., 2017. Additionally, we added the dot plots with 3-5 genes per cluster that we used to annotate the clusters presented from previously published dataset (Furlan et. al., 2017). Please see updated panel a Supplementary Fig. 1.

3. Sox10 immunostaining is not clear in some images (Fig. 1). Consider increasing contrast so this is easier to visualize.

   We improved the quality of Sox10 staining in Fig.1 and Supplementary Fig.3

4. PNMT expression is not clear and should be repeated with antibody staining.

   We chose a very sensitive and reliable fluorescent RNA in situ hybridization with RNA scope technology to visualize the cells on the onset of *Pnmt* expression. During revision we repeated *Pnmt* mRNA *in situ* hybridization for three stages (E13.5, E14.5, and E16.5) to show a reliable signal. Please see the new Supplementary Figure 4. Unfortunately, the batch of PNMT antibody that we used previously was finished and this antibody in discontinued. We did not find any other reliable type of this antibody.

5.Was there any impact on male rat embryos?

   We never discriminated the gender in the embryological studies. Thus, both sexes are always included, without any specific distinction.

6.Reference to Fig 6e and 6c needs to be checked.

   References to these panels in the mentioned figure are now fixed. —

7.Titles not needed in the figures.

   We generally agree with the reviewer. However, our figures are complex and not easy to grasp, which we tested on our co-workers. Thus, after experimenting with figure design, we came to the conclusion that the titles in the figures are indeed helpful and assist the understanding when the separate experiments are combined in one large figure. We deiced to keep the most critical titles as logical separators.

Reviewer # 3

We thank the reviewer for the raising very interesting and important questions. We did our best to answer them experimentally and we sincerely hope that our responses will make the reviewer happy.

1. The main manipulation in the paper, s.c. 5-HTP 20 mg/kg (w/o peripheral decarboxylase inhibitor), is not studied with regards to its pharmacokinetic and pharmacodynamic characteristics. Two papers are referenced (Udenfriend et al., 195; Mitchell et al., 1983), but neither of them assessed 5-HTP or 5-HT levels in the embryonic compartment. Without proper references or original data, this is a "black box" manipulation.

1. We agree with the reviewer that it is important to show that the treatment of pregnant females with precursor of serotonin indeed results in elevation of serotonin in the embryonic compartment. For these purpose we performed new measurements of 5HT in placenta and E14.5 fetuses following the administration of 5HTP in mouse. Our new results indicate the reliable increase of 5HT in embryos followed by 5HTP administration. Please check the new figure 2b and the following text:

   "… administration of 5HTP to pregnant females led to a significant increase of 5HT concentration in the placenta and trunks of E14.5 embryos (Fig. 2b), and caused enhanced release of 5HT by the fetal adrenal glands (Fig. 2c), as measured by HPLC"

2. We are thankful to the reviewer for pointing that the embryos were not assessed in the cited papers, and we apologize for providing the wrong references. Therefore, we performed our own experiments reported above, and we also included the citations of two papers, which show that 5HT concentration changes in murine uterus after 5HTP administration (Udenfriend et al., 1957, Bogdansky et al., 1958).
   This is how the text is appearing now:

   "Thus, the administration of 5HTP to pregnant rodent females causes a stable and physiological increase of 5HT concentration in embryos, as it was previously shown for other tissues including the uterus [32, 33]"

2. Regarding extended figure 1, the authors should clearly state that these data have been previously published (Furlan et al., 2018). (also no single cell analysis methods section).

We apologize for unclear reference of the fact that the data presented on Supplementary figure 1 was previously published. In the revised manuscript we clearly indicate that the data was previously published:

"Firstly, to address the role of 5HT signaling system in adrenal gland development, we analyzed the expression of related enzymes and receptors using single cell transcriptomics dataset of chromaffin and sympathetic development at E12.5 and E13.5 stages published previously [3] (Supplementary Fig. 1a)"

And in the figure legend

"t-SNE embedding of single cell RNA profiles (top panels) and dot plot for major genes, defining clusters (bottom panels) in the murine sympathoadrenal anlagen at E12.5 and E13.5 previously published by Furlan et. al.[1]"

We substantially revised the method section to explain how the diagrams were obtained:

"Re-analysis of single cell transcriptomic data published by Furlan et al., 2017

We re-analyzed single cell RNA-seq data of mouse adrenal gland from Furlan et al., 2017 [6] . Gene counts were obtained from GEO database (GSE99933). Gene count matrix was analyzed with a standard Seurat (version 3.0.2) workflow[78]. We used the original embeddings and clustering from [6] (Figures 5B and 5G), downloaded from the published pagoda apps: http://pklab.med.harvard.edu/cgi-bin/R/rook/nc.SS2_16_249-2/pathcl.json and http://pklab.med.harvard.edu/cgi-bin/R/rook/nc.SS2_16_250-2/pathcl.json (json slots embedding/data for the embedding and colcols/clusters/data for the cluster labels).

Seurat function FeaturePlot and DotPlot were used to plot gene expression in individual cells on the embedding and average gene expression in the clusters as dot plots, respectively."

3. Provide for extended figure 1 numerical quantification of gene expression levels averaged by cluster. This will help clarifying the following issues:

Following the reviewer's recommendation, we provided the numerical quantification for the dot plots and individual genes plotted on the *t-SNE* embedding. Please check the updated Supplementary figure 1. We also generated the new single cell transcriptomics datasets, aiming to reveal the effects of 5HTP treatment. The new dataset is in consistency with previously published data from Furlan et al.. Please see new Figure 5.

a. In extended Figure 1B it seems that Htr3a/3b is not only expressed in bridge cells but also in SCPs, PC, and sympathoblasts at E12.5 while at E13.5 this expression is restricted to bridge cells, PCs and some sympathoblasts. The authors should report this in the results section.

We agree with the reviewer, and it is indeed the case that at E12.5 the expression of *Htr3a/Htr3b* spans across "bridge" cells, a subset of SCPs and a subset of cycling cells. Please note that cycling cells at these stage resemble "bridge" cells based on their gene expression. *Htr3a/Htr3b* is also weakly expressed in a minor population within chromaffin cells and sympathoblasts. At E13.5 the majority of the "bridge" cells show high expression of *Htr3a*. Only a minor portion of cycling SCP cells and few sympathoblasts express *Htr3a*.

Following the reviewer's recommendation, we improved the description of the gene expression in the result section:

"). As shown earlier by Furlan et al., chromaffin cells originate from SCPs and the differentiation progresses through the transitory "bridge" population. Thus "bridge" cells are immediate progenitors of chromaffin cells. According to our analysis at E12.5, Htr3a/3b (encoding 5HT-receptor 3A/3B) are strongly expressed in the population of "bridge" cells and are only sporadically expressed in cells from other clusters. At E13.5, Htr3a/3b specifically mark the "bridge" cell population and also appear to be present in a minor portion of sympathoblasts (Supplementary Fig. 1b)."

b. In extended Figure 1C it seems that DDC+ is not restricted to chromaffin cells only but it is also expressed in other cell types, i.e. bridge cells, PCs, and sympathoblasts at E12.5 and at E13.5. The authors only discuss DDC expression for chromaffin cells (line122). How does DDC expression in other cell types affect the synthesis of 5HT at these developmental stages?

We are thankful for the reviewer's careful attention to the details of the gene expression in developing adrenal gland. Indeed, *Ddc* has a wide expression pattern among different cell types in the developing sympatho-adrenal complex at E12.5 and E13.5. However, the strongest expression is observed in chromaffin cells, as evident from the single cell transcriptomic data (improved Supplementary Fig. 1c). Accordingly, we improved the description of gene expression in the results:

"It turned out that nearly all cells in AM express Ddc gene encoding the enzyme responsible for decaboxylating 5HTP (5-hydroxytryptophan) into 5HT"

In order to check if the expression of *Ddc* in SCPs and "bridge" cells allows them to accumulate 5HT we carefully analyzed the immunostainings of adrenal gland sections from E13.5 *Htr3a*$^{EGFP+}$ embryos for Sox10, 5HT and EGFP revealing the *Htr3a* expression. According to this analysis, SCPs remain 5HT negative, and only few *Htr3a*$^{EGFP+}$ cells show 5HT immunopositivity. Please see new Supplementary Fig 4a. Moreover, we carefully calculated the proportions of the 5HT immunopositive cells among *Htr3a*$^{EGFP+}$ cells and TH^{+} chromaffin cells. Please see the updated bar graphs in Fig.1 and Supplementary Fig. 3. Due to this careful analysis, we were able to conclude that no SCPs and only a negligible portion of *Htr3a*$^{EGFP+}$ cells appeared 5HT immunopositive. We updated the text in the manuscript accordingly:

"At the same time, SCPs did not show 5HT immunoreactivity (Supplementary Fig. 4a), and only 1.7-5.1% of all Htr3a^{EGFP+} *cells were Htr3a*$^{EGFP+}$*/TH*$^{-}$*/5HT*$^{+}$ *(5HT-positive "bridge" cells) at E12.5-E14.5 (Fig. 1e, j, o)."*

Therefore, according to these new data, it became clear that despite *Ddc* expression, SCPs remain 5HT negative, and only the minor portion of "bridge" cells stays 5HT positive, which may contribute to the overall 5HT level in developing adrenal medulla.

c. Expression of Slc29a4 as well as other monoamine transporter genes seem not restricted to chromaffin cells either, but to extend to other cell types. Quantification and appropriate discussion of these findings should be mentioned. How does expression of these genes in other

cell types affect the chromaffin cell paracrine modulation and the overall hypothesis? Could it be that bridge cells also exert an autocrine modulation via 5HT signaling?

To address the reviewer's question, we performed a careful analysis of *Slc29a4*, (the gene coding for plasma membrane monoamine transporter, responsible for the transport of serotonin into cells), *Slc18a1* and *Slc18a2* (the genes coding for vesicular monoamine transporters, responsible for release of 5HT from the cells) (Supplementary Fig. 1). We also calculated the proportions of $5HT^{+}$ cells in AM (Fig. 1) and ZO (Supplementary Fig. 3).

The new data indicate that "bridge" cells and sympathoblasts expresses monoamine transporter for 5HT transfer in and out of the cells. Some sympathetic neurons are known to be serotonergetic[4, 5]. Therefore, the expression of these transporters is expected. However, the proportion of sympathoblasts in the developing adrenal medulla at E11.5-E13.5 is negligible (please see Kameneva et al., 2021, Nat. Genetics), and, therefore, sympathoblasts cannot contribute substantially to 5HT supply in adrenal medulla.

As for the "bridge" cells, the single cell transcriptomics predicted expression of serotonin synthesis and transport genes, suggesting that 5HT is released by "bridge" cells to the neighboring cells.

Following the reviewer's recommendation, we carefully analyzed the proportions of the 5HT immunopositive cells among *Htr3a*$^{EGFP+}$ cells and found that $5HT^{+}$ "bridge" cells (*Htr3a*$^{EGFP+}TH^{—}5HT^{+}$) represent 1.7-5.1% of all *Htr3a*$^{EGFP+}$ cells at E12.5-E14.5 in AM. Therefore, the limited autocrine modulation of "bridge" cells via 5HT signaling cannot be excluded, and we added this note to the figures (Fig.1p) and in the text.

Next, we found that the proportion of $5HT^{+}$ cells among TH^{+} early and mature chromaffin cells combined (*Htr3a*$^{EGFP+}TH^{+}5HT^{+}$ and *Htr3a*$^{EGFP—}TH^{+}5HT^{+}$) was 32% at E12.5 and grew up to 77.8% at E14.5. Therefore, we concluded that the proportion of 5HT immunopositive early chromaffin and chromaffin cells overweighs the proportion of 5HT immunopositive "bride" cells.

Thus, we mainly observe the paracrine regulation: chromaffin cells release 5HT that acts on the neighboring "bridge" cells. This major mechanism is augmented by the autocrine regulation by "bridge" cells releasing 5HT, which acts on the "bridge" cells.

Accordingly, we updated fig.1p where we added the mode of autocrine regulation into the schematics of 5HT signaling during early chromaffin cell development. Also please check the new bar plots for the proportions of the 5HT immunopositive cells among *Htr3a*$^{EGFP+}$ cells and TH^{+} chromaffin cells in Fig.1 and Supplementary Fig. 3.

Please see improved text:

"Next, our analysis revealed that already at E12.5, nearly 32% of early and mature chromaffin cells are $5HT^{+}$ in the AM (Fig. 1e). Soon after, around 58% and 77.8% of chromaffin cells became $5HT^{+}$ in the AM at E13.5 and E14.5 correspondingly (Fig. 1f-o). At the same time, SCPs did not show 5HT immunoreactivity (Supplementary Fig. 4a), and only 1.7-5.1% of all Htr3aEGFP+ cells were Htr3a^{EGFP+}*/*$TH^{—}$*/*$5HT^{+}$ *(5HT-positive "bridge" cells) at E12.5-E14.5 (Fig. 1e, j, o). In ZO the proportion of* $5HT^{+}$ *cells among* TH^{+} *and Htr3a*$^{EGFP+}$ *cells followed a*

similar pattern (Supplementary Fig. 3). Therefore, chromaffin cells contribute most of the local 5HT to the surrounding and neighboring cell types in the adrenal gland. In line with these observations, at E12.5-E13.5, Htr3a^{EGFP+}/TH$^{—}$ "bridge" cells (sensitive to 5HT) are intermingled with chromaffin cells in the primordium of AM and ZO (Fig. 1a, e, Supplementary Fig. 3a-h), and are susceptible to 5HT generated by neighboring chromaffin cells. Based on this, we propose a new mechanism of a paracrine control, where chromaffin cells release 5HT acting on neighboring "bridge" cells, with some contribution of the autocrine regulation, where few 5HT^{+} "bridge" cells act on themselves and other HTR3A^{+} "bridge" cells in their vicinity. As the SCPs and few chromaffin cells express other receptors to 5HT, additional modes of local autocrine and paracrine control might be also present (Fig. 1p).

4. In Figure 1 it is not how 5-HT colocalizes (it is also missing from the Venn diagram). Please provide a percentage where the numerator is 5HT and the denominator is either TH or Sox10/EGFP. Also, the analysis of the cell count should be explained in more detail. The authors should report the percentages of overlap for every cell type quantification and the statistics on the percentages rather than total numbers.

We agree with the reviewer and we have added the new calculation of 5HT co-localization with TH^{+} and *Htr3a*$^{EGFP+}$ cells on Venn diagrams for stages E12.5-E14.5 for both the adrenal medulla (updated Fig 1, panels d, i, n) and organ of Zuckerkandl (updated Supplementary Fig 3, panels d, i, n). We also calculated the percentage of 5HT positive cells among TH^{+} and *Htr3a*$^{EGFP+}$ cells and plotted them as bar graphs. Pure SCPs, before onset of *Htr3a* and TH expression remain 5HT negative (please see new Supplementary data fig 4a), therefore the further analysis of SOX10/5HT co localization at E12.5 and E14.5 was omitted.

Further, we carefully analyzed the proportions of the 5HT immunopositive cells among *Htr3a*$^{EGFP+}$ cells and found that 5HT^{+} "bridge" cells (*Htr3a*$^{EGFP+}$TH$^{—}$5HT^{+}) represent 1.7-5.1% of all *Htr3a*$^{EGFP+}$ cells at E12.5-E14.5 in AM. The proportions of 5HT^{+} cells among TH^{+} early and mature chromaffin cell was 32% at E12.5 and grew up to 77.8% at E14.5. Therefore, we conclude that the proportion of 5HT immunopositive early and mature chromaffin cells overweighs the proportion of 5HT immunopositive "bride" cells.

Furthermore, we improved the description of the cell type quantification in the method section. In brief, all cell types were manually quantified using cell counter plugin in ImageJ, and the data are now represented as percentages as requested.

5. Th is primarily expressed in chromaffin cells, however DDC seems to be expressed by a larger cell population including bridge cells based on the transcriptomic data. How do the authors explain that chromaffin cells are the primary source of 5HT when also other cells have the potential to synthetize 5HT given their expression of DDC (especially as Tph loss of function experiments do not affect Sox10 cell numbers)?

We are thankful to the reviewer for pointing that *Ddc* is indeed expressed in all population of cells within adrenal medulla according to the single cell RNA expression data. As we already mentioned above, this forced us to pay a careful

attention to the distribution of 5HT immunopositive cells within developing chromaffin organs. We found that at all investigated stages, the $5HT^{+}$ early (*Htr3a*$^{EGFP+}$$TH^{+}5HT^{+}$) and mature chromaffin (*Htr3a*$^{EGFP—}$$TH^{+}5HT^{+}$) cells clearly overweighed the proportions of $5HT^{+}$ "bridge" cells (*Htr3a*$^{EGFP+}$$TH^{—}5HT^{+}$):

32% of $5HT^{+}$ early and mature chromaffin cells among all TH^{+} cells vs 1.7% of *Htr3a*$^{EGFP+}$$TH^{—}5HT^{+}$ cells among all *Htr3a*$^{EGFP+}$ cells at E12.5;

58% of $5HT^{+}$ early and mature chromaffin cells among all TH^{+} cells vs 2.6% of *Htr3a*$^{EGFP+}$$TH^{—}5HT^{+}$ cells among all *Htr3a*$^{EGFP+}$ cells at E13.5;

77.8% of $5HT^{+}$ early and mature chromaffin cells among all TH^{+} cells vs 5.1% of *Htr3a*$^{EGFP+}$$TH^{—}5HT^{+}$ cells among all *Htr3a*$^{EGFP+}$ cells at E14.5 in AM.

The similar distribution patterns were observed for ZO, with less than 1% of *Htr3a*$^{EGFP+}$$TH^{—}5HT^{+}$ "bridge" cells among all *Htr3a*$^{EGFP+}$ cells at all stages. Therefore, we concluded that early and mature chromaffin cells are the major cellular contributors to the 5HT in the developing adrenal organs.

Thanks to reviewer's recommendation, we found a minor population of "bridge" cells, which is 5HT immunopositive, and we updated the proposed mechanism with the notion that autocrine mode of regulation ("bridge" cells release 5HT which can act on "bridge" cells) is also possible.

Based on our observations, we find that mainly chromaffin cells in developing chromaffin organs are 5HT immunopositive, even though other cell types express *Ddc* (based on the single cell transcriptomics data) and have a potential to synthetize 5HT from 5HTP. Despite this potency, we do not see this happening in experimental analysis. This can be explained by multiple reasons. For instance, additional levels of control (at the post-transcriptional and protein levels) might take place. Please note that we do not claim that 5HT in chromaffin cells appears as a result of synthesis only, as it might be as well transported inside of the chromaffin cells, and become accumulated from the systemic circulation.

When it comes to $SOX10^{+}$ cells in *Tph* knock out, to our knowledge, there is no evidence for the *Tph* requirement for the development of SOX10 cell lineage. Besides, SCPs depend mostly on the availability of external innervation, coming to the developing tissues from elsewhere. In line with this, the number of $SOX10^{+}$ cells is not affected in the adrenal medullae in case of *Tph1*$^{-/-}$*; Tph2*$^{-/-}$ and *Tph1*$^{-/-}$*; Slc6a4*$^{-/-}$ (Supplementary Fig. 7) in AM and SCG (consistent across the embryo). In the same way, the number of TH^{+} chromaffin cells (early and mature collectively) was not specifically affected in the AM of *Tph* knock out embryos analyzed. Thus, we conclude that *Tph* loss does not have a major effect on the development of chromaffin cell lineage. Please see the updated Supplementary Fig. 7.

We added the clarification of these observations in the Discussion part:

"Overall, the paracrine and autocrine role of 5HT in developing chromaffin organs results in two important aspects related to health and survival: protection from chromaffin tissue overgrowth or neoplasia, and prevention of excessive catecholamines and catecholamine-controlled behavior."

5. How do the authors rule out a possible autocrine regulation of 5HT onto bridge cells?

According to our new data, we found 5HT^{+} "bridge" cells (*Htr3a*$^{EGFP+}$TH^{-}5HT^{+}) represent 1.7-5.1% of all *Htr3a*$^{EGFP+}$ cells at E12.5-E14.5 in AM, and we agree that autocrine regulation of "bridge" cells is possible and important. We corrected the concluding scheme. Please see the new concluding panel in Fig.1p and the updated text:

"Based on this, we propose a new mechanism of a paracrine control, where chromaffin cells release 5HT acting on neighboring "bridge" cells, with some contribution of the autocrine regulation, where few 5HT+ "bridge" cells act on themselves and other HTR3A+ "bridge" cells in their vicinity. As the SCPs and few chromaffin cells express other receptors to 5HT, additional modes of local autocrine and paracrine control might be also present (Fig. 1p)"

6. What is the proportion of Sox10/5HT/HTR3A at E13.5? For this reason, the authors should provide a quantification of Sox10/5HT/HTR3A at E13.5.

We performed additional staining of Sox10/5HT/HTR3A at E13.5 and we did not find SOX10^{+}5HT^{+} cells. Please see the new Supplementary figure 4a.

So again, the main open question that needs to be addressed is whether bridge cells also produce 5HT, and thereby can act in an autocrine way to regulate their differentiation.

According to our new data, we found 5HT^{+} "bridge" cells (*Htr3a*$^{EGFP+}$TH^{-}5HT^{+}) represent 1.7-5.1% of all *Htr3a*$^{EGFP+}$ cells at E12.5-E14.5 in AM, and we agree that autocrine regulation of "bridge" cells is possible and important. We corrected the concluding scheme. Please see the new concluding panel in Fig.1p and the updated text:

"Based on this, we propose a new mechanism of a paracrine control, where chromaffin cells release 5HT acting on neighboring "bridge" cells, with some contribution of the autocrine regulation, where few 5HT+ "bridge" cells act on themselves and other HTR3A+ "bridge" cells in their vicinity. As the SCPs and few chromaffin cells express other receptors to 5HT, additional modes of local autocrine and paracrine control might be also present (Fig. 1p)"

7. For extended figure panels 5 e, f, g, and h, please specify whether the N is the number of litters or pups. This experiment seems underpowered (N = 3) to draw the conclusion that proliferation and apoptosis rates are not affected by 5HTP treatment.

During the revision, we performed the major experiment analysis of cell proliferation and cell cycle progression with the double thymidine analogue labeling. Now the power of the analysis became sufficient to track the difference in cell cycle dynamics. Please see new data in the new figure 4 and Supplementary figure 6.

As for cleaved Caspase 3 immunostaining, we reanalyzed out data and we added more embryos to the control and treatment group to increase the power of the

analysis. In the revised version of the experiment presented now in the new Fig.4, panel e, 5 embryos per condition were analyzed. Virtually no cleaved CASP3^{+} cells were found in both AM and ZO, as there was apoptosis and no apoptosis enhancement due to 5HTP treatment in chromaffin organs.

8. If as mentioned in point 5 apoptosis and proliferation are not altered, how do the authors explain the reduction in AM size. For instance, if bridge cells have a smaller cell size than chromaffin cells a consequence on AM size is feasible, but the authors need to better study/discuss this.

1. We are thankful to the reviewer for the opportunity to further explore the observed phenomenon and to figure out the possible mechanism of the 5HT regulation of chromaffin cell lineage development. We hypothesized, based on the reviewer's suggestions that the changes in the cell cycle progression may reveal the reasons for the observed phenotype.
2. For these purpose, we performed a new major experiment to measure the cell cycle progression in different cell populations with double thymidine analogues labeling. More specifically, we gave two pulses of thymidine analogs EdU and CldU at 4-hour intervals together with the 5HTP treatment to label the cells during S-phase of the cell cycle and to analyze the proportions of cells, which incorporated EdU only, CldU only, and both nucleotides. The difference in proportions of labelled cell reflected the changes in cell cycle progression. The results are summarized in the new figure 4.
3. We started the treatment at E11.5 with one dose of 5HTP, and we gave the second dose of 5HTP and consecutive pulses of thymidine analogs at E12.5. We collected embryos in the evening of E12.5, therefore embryos analyzed for this experiment are different stage from embryos presented on Fig. 3 and Supplementary Fig. 5 (E13.5), and we expect to see slightly different numbers of cells.
4. The proportions of cells incorporated EdU only was lower and the proportion of cells which incorporated both EdU and CldU was higher among "bridge" cells in AM of embryos from 5-HTP treatment group. At the same time the number of proliferating cells (cells that incorporated at least one label) was not different among SCPs, "bridge" cells, early chromaffin cells and mature chromaffin cells. These results allowed us to conclude that "bridge" cells have prolonged cell cycle during our observation period under the treatment with 5HTP. Therefore, as reviewer correctly hypothesized, the change in cell cycle length results in less precursor cells being available for differentiation (please see significantly decreased cell numbers of "bridge" cells in addition to early chromaffin cells in AM at E12.5 according to our new data shown in figure 4b). This prolonged cell cycle of progenitors causes the downstream deficit of chromaffin cells and results in the reduced size of adrenal gland medulla. No changes in cell cycle progression was observed for other cell populations. It is also important to mention that there are negligible cells, which incorporate EdU or CldU among mature chromaffin cells, as they temporarily exit cells cycle at the beginning of maturation.

5. To make sure we did not miss any important difference between treated and control conditions, especially when it comes to the differentiation dynamics and the structure of transitory populations, we performed another big experiment

using single cell transcriptomics. Our single cell sequencing was based on Smart-seq2, the deepest and most expensive method of sequencing, and we aimed to compare the differences in lineage trajectories, differentiation, cell populations in control and in 5HTP-treated conditions. As a result, we revealed that the differentiation trajectory and the structure of all populations (identity and the proportions of cell types), including SCPs, “bridge” and chromaffin cells remained the same in treated and untreated conditions. At the same time, the comparison of cells isolated from 5HTP-treated and control AMs and ZOs from several embryos revealed significant differences in differential splicing of genes responsible for progression though the cell cycle (*Apobec3* and *Cenpa.AS2*). This change in differential splicing might be mediated by *Cwc22* gene, responsible for the regulation of splicing, which is differentially expressed in 5HTP-treated vs control group and has the highest expression in “bridge” cells among the other cell types. Therefore, deep analysis of individual transcriptomes is in agreement with the observed 5HT-mediated changes in ”bridge” cells cell cycle progression. Please, see new Figure 5.

8. Have the authors quantified Sox10 and HTR3a (exogenous markers) cell number in Tph-/- Slc6a4 -/- and Tph1-/- Tph2-/- in AM?

Are Sox10+ and HTR3a (exogenous marker) cell numbers reduced in AM of Tph-/-?

Quantifying these parameters would help clarifying if lack of 5HT is stalling cells into precursors of bridge cells or even in bridge cell stages.

In accordance with the reviewer’s suggestion, we have quantified the numbers of $SOX10^{+}$ cells in *Tph*$^{-/-}$ *Slc6a4* $^{-/-}$ and *Tph1*$^{-/-}$ *Tph2*$^{-/-}$ in AM as well as in sympathetic chain ganglia. We did not find any significant differences in the SOX10 cell number in comparison with C57BL/6 animals. This might be explained by the fact that SCPs depend on the density of neuronal fibers coming from elsewhere, and not so much on the intra-tissue environment. We added the new data into the Supplementary Fig. 7f

We also performed mRNA *in situ* hybridization for *Htr3a* in *Tph1*$^{-/-}$ *Tph2*$^{-/-}$ *Slc6a4* $^{-/-}$ knock out by RNA scope and found that “bridge” cells are present in the knockout animals. Please see Supplementary Figure 7 g. Because RNAscope is sensitive to quality of the tussue, we could not achive good statistical power for this specific experiment using the previous batches of embryos.

Please see new description of the results:

”Of note, the numbers of SCPs, were not changed in AM and SCG in control and KO embryos (Supplemetary Fig.7), because SCPs depend on the local innervation coming from elsewhere. The expression of Htr3a mRNA was evident in Tph1$^{-/-}$*;Tph2*$^{-/-}$*;Scl6a4*$^{-/-}$ *E13.5 adrenal glands, which indicated that “bridge” cells are still present in the KOs (Supplementary Fig. 7 f).”*

Overall, based on our observation of KO embryos, we do not report any specific changes, which would be related to the differentiation of SCPs or ”bridge” cells. Correspondigly, the adrenal medullae in these knockouts do not become bigger.

We added the clarifying line in the discussion:

”The proposed mechanism of chromaffin cell number control via 5HT is unidirectional, as the complete elimination of 5HT and pharmacological blockage of HTR3A receptor do not result in overgrowth of chromaffin cell organs. This goes in line with previous reports showing that the excess of 5HT has stronger effects on brain development as compared to the lack of 5HT [58, 59].”

The authors justify the loss of function as a developmental delay, but they do not provide evidence of this delay as for instance cells being stalled in precursors or immature forms. It could also be that cells may die when 5HT is lacking so apoptosis markers should be considered. If these data have not already been generated, the authors should discuss these possibilities.

We apologize for not reporting these results in a clear way in the previous version of the manuscript. Below we provide the major arguments:

1. The lack of serotonin is known to cause developmental delay from the previous studies[6, 7].
2. In order to have the internal control of the systemic developmental delay, we decided to analyze the cell numbers within the sympathetic chain ganglia (SCG), as those represent a related cell type with an independent origin, being immediately derived from the early migratory streams of the neural crest. Thus, we reasoned that if the changes in the sympathetic ganglia will follow the same pattern as the changes in the adrenal medulla, the observed changes must be non-specific due to general developmental delay or other abnormality. If the changes in cell numbers within SCG and AM would have different patterns, we would discover the specific effect of reduced 5HT on chromaffin cells.
3. Thus, we have calculated the number of TH^+ sympathoblasts in several pairs of SCG and TH^+ chromaffin cells within several sections of adrenal medullae. Our analysis showed the similar reduction rate in both organs, indicating the general developmental delay and the absence of specific effects resulting from reduced 5HT in adrenal glands of the knock out embryos.
4. We have followed the similar logic and used SCG ganglia as an internal control tissue for the experiment with 5HTP-treatment (Fig. 2). In this set of experiments, we were able to find specific action of 5HT, i.e decrease of the chromaffin cell population in adrenal medulla in comparison with no changes in sympathoblast cell population in the SCG. Please see new Fig.3 and Supplementary Fig. 5.

9. For the whole paper the statistical approach should be better explained and revisited substantially. Mann Whitney U test is not appropriate for any comparison involving more than 2 groups where more than one variable is considered. Particularly for drawing conclusions on behavior and pharmacological treatment.

We are very thankful to the reviewer for the motivation to improve the statistical approach for the whole paper. Accordingly, we have added the separate section in the methods describing the choices of statistical methods:

“Statistical analysis was done using GraphPad Prism 8.1.1 software. All datasets were checked for normality with Shapiro-Wilk test, as this test reliably works for small datasets. For datasets with normal distribution double sided unpaired t-test was applied. For datasets which failed a test for normal distribution, Mann-Whitney test was applied. For comparisons between multiple groups for one variable the one-way ANOVA test was applied (Fig. 3d, Fig. 6a,c,l, Supplementary fig. 7 d,e,f). In case one-way ANOVA test was showing significant differences, Tukey’s multiple comparison test was used for a robust pairwise comparison of groups with unequal size (every mean to every other mean) or Dunnet’s multiple comparison test for a pairwise comparison of groups to a control group.

In the analyses of statistical significance in catecholamine measurement as well as in behavioral tests (Fig. 7, Supplementary Fig.8) we have chosen the pairwise comparison within genders. The reason for it is that the treatment with 5HTP or 1 hour restrain was applied at the stage, when the sexual dimorphism is not established in the forming adrenal glands and, consequently, the treatment equally affects males and females. However, adrenal glands have sexual dimorphism at postnatal stages and therefore must be analyzed separately, since the combined analysis of these groups can mask the effects of our prenatal treatment. We have calculated statistical significance with Mann-Whitney test within genders between treated and control animals as this test as this test is applicable to small samples and can evaluate statistical significance regardless of the normality of data distribution and homoscedasticity of the data. For these experiments the analysis was performed in SigmaPlot 12.1”

All figure legends are now containing the detailed description of statistical methods applied.

Interactions and main effects should be discussed and should be supported by an appropriate statistic:

a. Figure 1 display percentage graphs and related statistics

We displayed the percentage graphs with mean and SD

b. Figure 2d 2-way anova should be performed

We kept the unpaired double sided ttest as the test for statistical significance because two-way ANOVA is not necessary in this case, as we did not intend to compare the medulla size to the cortex size. In this case, we applied the pairwise comparison: the control medulla size is compared to the treated medulla size. Moreover, we made sure that the data distribution is normal, which justified the use of the unpaired double sided ttest.

c. Figure 3F one-way anova. (stats are missing completely as is)

We agree, and we applied one-way ANOVA.

d. Figure 4C two-way anova

Agree, and we removed the old version of the figure.

e. Figure 5 one-way (g, h), and two-way (i) anova

We applied one-way ANOVA in all cases. For i, there was no intention to compare TH^{+} to $SOX10^{+}$ cell numbers. Therefore, the appropriate comparison with one variable was applied.

f. Figure 6 two-way anova and also provide dot plot graphs to see how the single data points distribute.

We agree with the reviewer.

1. It will not be possible to apply two-way ANOVA here, as not all datasets have normal distribution, which is the necessary assumptions for the test.
2. Moreover, in our case, the pairwise comparison within genders will better reflect the biological meaning of the experiment. The reason for this is that the treatment with 5HTP or 1h restrain was applied at the stage when the sexual dimorphism is not established in the forming adrenal gland medullae and, therefore, the treatment *bona fide* equally affects both males and females. However, the adrenal glands have sexual dimorphism at postnatal stages and therefore must be analyzed separately. We have calculated statistical significance with Mann-Whitney test within genders between treated and control animals, as this test evaluates statistical significance regardless of the normality of data distribution and homoscedasticity of the data.
3. We changed all bar graphs to the violin plots with individual values shown together with median and quartiles.

g. Figure 7 one-way anova

One-way ANOVA is applied.

10. For the behavior, the authors should discuss potential effects of 5HTP on the mother that are then producing effects on the pups and how cross-fostering experiments might disambiguate various alternative hypotheses.

We agree that the 5HTP-treated mothers can affect the newborn progeny in multiple ways that are hard to predict.

1. Since we cannot predict all such ways of influence, and the foster mother experiments might be traumatic and stressful as well (and might not even answer the question), we cannot claim that the results of postnatal tests are only rooted in decreased adrenals and embryonic influence from the mother. Instead, we *bona fide* suggest that outcomes of these experiments go in line with all other, primarily, embryonic observations. Also, the results of the mild stress and the results of 5HTP treatment show the same pattern in postnatal progeny and both result in the

corresponding decrease of medullae size, which cross-validates these results and fosters our general conclusion, especially given that the mild stress naturally causes the 5HT increase in pregnant females. Still, the main supporting argument is that prenatal effects and postnatal effect are likely connected and correlate via the decreased adrenals.

We updated the text in the revised manuscript to clearly explain this issue:

"Although our data from mice and rats appear to be consistent, we do not claim that the results of postnatal behavioral tests are only rooted in decreased adrenals and embryonic influence from the mother. Here, we bona fide suggest that outcomes of these experiments go in line with each other and, primarily, embryonic observations. Also, the results of the mild stress and the results of 5HTP treatment show the same pattern in postnatal progeny, being associated with decreased AM size. Overall, the prenatal effects and postnatal effect are likely connected and correlate via the reduced number of chromaffin cells in adrenal glands."

2. For the behavioral study and catecholamine analysis of animals prenatally exposed to the elevated levels of 5HT, pregnant female rats received extremely low dose of 5-HTP (1 mg/kg), and we demonstrated that the dose is sufficient to increase placental and fetal 5HT level (Fig. 2b), and results in long-term decrease of adrenal medulla size as well as affects the progeny behavior.

According to the literature data, 5-HTP exposure (20-100 mkg/kg) provides reversible increase of brain 5HT. For example, 5HTP oral treatment (10 mg per kg) results in brain 5HT increase up to two hours. At 4h post-treatment brain 5HT level decreases dramatically, to and by 24h returned to control-like level. Moreover, 5-HTP administration causes a transient elevation of 5-HT in the urine within 1-4 hours returning to control level by 6-8h after oral gavage or i.p. injection[8]. Similar results of 5-HNP administration described in man[9].

Above mentioned literature data allow us to hope that our 5HTP treatment did not significantly affect the behavior of nursing mothers. Since we have considerable experience with the modulation of serotonin levels in pregnant rodents (5HT, 5-HTP and pCPA application), the pregnant females were under observation before and after delivery. We were ready to replace the nursing mother in case of problems. However, no signs of miscarriage of the offspring were observed. The size and body weight of newborns did not differ in control and experimental mothers.

11. For the behavior, is N the number of litters or pups?

For the behavior study, the individual adult animals are considered as data points.

12. Naming figure 6 a-c "pharmacological study" is confusing.

We agree with the reviewer, and we changed the name to the "major catecholamines" to avoid confusion.

13. Line 301-302 sentence does not make sense… negative for mycn amplification but correlated with mycn expression?

We apologize for the confusion caused by the previous version of the manuscript. The intended meaning was that while no cell line bears MYCN amplification (more than two copies of MYCN gene in a genome), these neuroblastoma cell lines differed in N-MYC expression and this high N-MYC expression was associated with HTR3A levels. To convey this meaning better and to reflect the results of western blot analyses (Fig. 6b), we have amended this section as follows:

"While all cell lines were negative for MYCN amplification (two copies of gene in the genome), HTR3A protein expression was associated with expression of major drivers of aggressive neuroblastomas, N-MYC and c-MYC [44, 45]*, or one of the core stemness factors, SOX2 (Fig. 6b)."*

14. Lines 357-358 – ref missing. Which species? Line 361 – which species?

For the line 357-358 "However biosynthetic enzymes TPH1 and AADC are expressed in the syncytiotrophoblastic cell layer of the placenta" the clarification that it is in mouse and human was added to the text and the reference to the Bonnin et al., 2011, Nature is added. Current the text is the following:

"The biosynthetic enzymes TPH1 and DDC are produced in the syncytiotrophoblastic cell layer of the murine placenta, which is in line with previous observations of in vitro placental 5HT neo-synthesis at E10.5-E18.5 in mice. In line with this observation, human placental fetal villi demonstrated a similar biosynthetic capacity during early gestation [68]*."*

For the line 361 "Later in the development, a progressive switch from placental to endogenous source of fetal 5-HT was demonstrated for the case of developing brain" the clarification that it is in mouse was added to the text. The updated text incorporates the following phrase:

"The combination of different 5HT sources and the switch from systemic (extraembryonic) to the local source of 5HT were previously noticed during embryonic brain development in mice [15]*."*

Reviewer #4 (Remarks to the Author):

In this study, Kameneva et al. propose a novel feedback loop regulating the production of mature chromaffin cells in the adrenal gland. According to the authors' model, Schwann cell precursor (SCP)-derived bridge cells act as chromaffin precursor cells that express the serotonin receptor 5-hydroxytryptamine receptor 3A (HTR3A). HTR3A, in turn, is activated by 5-HT secreted by early mature chromaffin cells. Upon increase of 5-HT levels, the generation of mature chromaffin cells is reduced and consequently the size of the adrenal gland decreased. This then correlates with altered expression of stress hormones and slight behavioral changes. Dissecting the impact of this signaling pathway in development of the adrenal gland may also pave the way for a better understanding of chromaffin organ-derived cancers, such as neuroblastoma.

This is a potentially interesting study, as the proposed regulatory feedback loop is novel. However, there are several major issues that need to be addressed prior to publication of this study. In particular, the data supporting a regulatory feedback loop between bridge cells and chromaffin cells need to be substantially strengthened. Likewise, the link between the findings made in development and those obtained from the tumorigenesis assays has to be better established.

> We thank the reviewer for asking reasonable and deep questions spending a lot of time on our work. We did our best, and we hope that additional experiments helped to resolve all key questions and issues.

Main points:

1. Although Htr3a is proposed by the authors to be expressed specifically in bridge cells, HTR3AEGFP also marks early chromaffin cells labelled by TH. In fact, at the relevant stages, the vast majority of Htr3aEGFP expression overlaps with TH expression (Fig. 1). Therefore, virtually all data could be interpreted as an effect of autocrine serotonin signaling on chromaffin cells (rather than by cross-regulation between bridge cells and chromaffin cells).

> We agree with the reviewer that the possibility of autocrine serotonin signaling on chromaffin cells needs to be carefully addressed. In the *Htr3a*^{EGFP} mice model the GFP is retained for a prolonged time after the active expression of *Htr3a* is over, as it was shown previously[10] and during our study.

1. Therefore, we now clarified the observed cell types with chromaffin organs as following:
   Htr3a^{EGFP+}TH^{-} - "bridge" cells
   Htr3a^{EGFP+}TH^{+} - early chromaffin cells
   Htr3a^{EGFP-}TH^{+} - mature chromaffin cells.

2. Next, in order to solve the issue, we performed detailed analysis of *Htr3a* RNA expression in tissue to reveal the expression of *Htr3a* RNA on the sections of E13.5 adrenal medullae from *Htr3a*$^{EGFP+}$ embryos by a sensitive fluorescent RNA *in situ* hybridization (RNA scope) combined with immunohistochemistry for EGFP (shows the expression of *Htr3a* in *Htr3a*$^{EGFP+}$ mice model) and TH (marker of chromaffin cells).

The combination of *Htr3a* mRNA *in situ* hybridization with TH clearly showed that the vast majority of TH^{+} are negative for the *Htr3a* mRNA expression. Please, refer to the new Supplementary Fig. 2b.

3. To improve on this issue even further, we measured the cell cycle progression in different cell populations building adrenal glands. For this purpose, we gave two pulses of thymidine analogs EdU and CldU at 4-hour intervals together with the 5HTP treatment to label the cells during S-phase of the cell cycle and to analyze the proportions of cells, which incorporated EdU only, CldU only, and both nucleotides. The difference in proportions of labelled cell reflected the changes in cell cycle progression. The results are summarized in the new figure 4.
4. We started the treatment at E11.5 with one dose of 5HTP, and we gave the second dose of 5HTP and consecutive pulses of thymidine analogs at E12.5. We collected embryos in the evening of E12.5, therefore embryos analyzed for this experiment are different stage from embryos presented on Fig. 3 and Supplementary Fig. 5 (E13.5), and we expect to see slightly different numbers of cells.
5. The results are summarized in the new figure 4. The proportions of cells incorporated EdU only was lower and the proportion of cells which incorporated both EdU and CldU was higher among “bridge” cells in AM of embryos from 5-HTP treatment group. At the same time the number of proliferating cells (cells that incorporated at least one label) was not different among SCPs, “bridge” cells, early chromaffin cells and mature chromaffin cells. These results allowed us to conclude that “bridge” cells are the only population of cells, which has prolonged cell cycle during our observation period under the treatment with 5HTP.
6. These new data, allowed us to improve the description of the mechanism of the 5HT-HTR3A mediated decrease of chromaffin cells. More specifically, the change in cell cycle length of “bridge” cells result in less precursor cells being available for differentiation into chromaffin cells in a given time and manifests in the reduced size of adrenal gland medulla. No changes in cell cycle progression was observed for other cell populations. It is also important to mention that there are sporadic (very few) cells which incorporate EdU or CldU cells among mature chromaffin cells, as they temporarily exit cells cycle at the beginning of maturation, and do not compensate for the shortage of precursor cells.
7. Please note that the new analysis revealed that “bridge” cells are also significantly reduced in numbers together with early chromaffin cells at the stage of cell cycle measurement (E12.5) in AM. Therefore, the downstream effect of prolonged cell cycle in “bridge” cells is manifested in their cell numbers and affects the cell numbers of their direct progeny chromaffin cells. The presence of cell number-related phenotype in “bridge” cells supports that there will be downstream effects in chromaffin population. Thus, we start seeing the phenotype already in “bridge” cells. Please check the new Figure 4b.
8. Finally, to make sure we did not miss any important difference between treated and control conditions, especially when it comes to the differentiation dynamics and the structure of transitory populations, we performed another big experiment using single cell transcriptomics. Our single cell sequencing was based on Smart-seq2, the deepest and most expensive method of sequencing, and we aimed to compare the differences in lineage trajectories, differentiation, cell populations in control and in 5HTP-treated conditions. As a result, we revealed that the differentiation trajectory and the structure of all populations (identity and the proportions of cell types), including SCPs, “bridge” and chromaffin cells remained the same in treated and untreated conditions. At the same time, the comparison of cells isolated from 5HTP-

treated and control AMs and ZOs from several embryos revealed significant differences in differential splicing of genes responsible for progression though the cell cycle (*Apobec3* and *Cenpa.AS2*). This change in differential splicing might be mediated by *Cwc22* gene, responsible for the regulation of splicing, which is differentially expressed in 5HTP-treated vs control group and has the highest expression in “bridge” cells among the other cell types. Therefore, deep analysis of individual transcriptomes is in agreement with the observed 5HT-mediated changes in ”bridge” cells cell cycle progression. Please, see new Figure 5.

9. To avoid any overstatement and to open up the future research direction we updated the text:

   “*As the SCPs and few chromaffin cells express other receptors to 5HT, additional modes of local autocrine and paracrine control might be also present (Fig. 1p).*”

Indeed, unlike claimed in the manuscript (lines 213ff), the proportion of Htr3aEGFP-positive cells is NOT increased in the adrenal medulla upon 5-HTP treatment (Fig. 3c), suggesting that the reduction of chromaffin cells in these mice is due to a direct effect of 5-HT on chromaffin cells. The proportion of Htr3aEGFP-positive cells is only (relatively slightly) increased in the organ of Zuckerkandl (Fig. 3e), but there, early chromaffin cell numbers are not changed, again suggesting that the phenotype is NOT coupled with altered differentiation of bridge cells.

Our new experiments (Fig. 4 and 5) clearly show that only *Htr3a*$^{EGFP+}$TH$^{—}$ “bridge” cells react to the increased level 5HT by slowing down their progression though the cell cycle. Consequently, in these new results we observe the reduction of “bridge” cell numbers in AM at E12.5 (new Fig. 4b). The slowing down of the cell cycle in “bridge” cells causes less cells precursor cells available the for generating chromaffin cells and leads to the eventual slower accumulation of chromaffin cell numbers. This understanding of adrenal developmental dynamics results from two new major experiments: single cell transcriptomics (showing the difference in cell cycle-related genes in “bridge” cells, Fig 5) and careful analysis of a cell cycle length in AM (Fig 4) and ZO (Supplementary Fig. 6). Any possible autocrine signaling in chromaffin cells will not lead to any significant change of their cell numbers because vast majority of mature chromaffin cells is proliferatively arrested and early chromaffin cells (that still rarely divide) are not changing their cell cycle length or label incorporation. Furthermore, we did more careful analysis of apoptosis and did not find any difference from our previous conclusions (see Fig 4e). We changed the main concluding scheme to outline these developments in our understanding (Fig. 4f).

To rule out any unexpected effect and to apply the most unbiased method to our study, we performed a single cell transcriptomics analysis based on Smart-seq2, the deepest and most expensive method of sequencing, to compare the differences in cell populations in control and in treated conditions separately in ZO and AM. This major experiment showed no critical difference in control and in 5HTP treatment when we did a very strict analysis including compositional defects and velocity of differentiation (using our experiences from Furlan et al., Science 2017, La Manno et al., Science 2018 and Soldatov et al., Science 2019). We did not show multiple statistical tests that delivered negative results due to their negative nature. Even our high competence in bioinformatics analysis of single cell data (please see the

aforementioned papers for confirmation) did not help us to understand the difference between ZO and AM phenotype under 5HTP treatment. The differentiation trajectory and the structure of all populations, including SCPs, bridge and chromaffin cells remained the same in ZO and AM according to their transcriptional profiles.

The incorrect phrase in line 213 is removed.

In the experiments shown e.g. in Fig. 3f (pharmacological treatment) or Fig. 5, only TH^+ chromaffin cells are taken into account; i.e. these experiments are in agreement with a direct effect of serotonin on chromaffin cells and don't support a role of paracrine serotonin signaling from chromaffin cells to bridge cells, as proposed in the authors' model.

1. The pharmacological study (presented in former Fig. 3f and current Fig. 3d) simply aimed at confirming that specific activation of HTR3A receptor, but not the other receptors, is responsible for the observed reduction of TH^+ cells. If the pharmacological agents would give us same effects as 5HT, then we would be sure about HTR3A receptor role in the mechanism of action. In parallel, the analysis of chromaffin cell population in various knock out mice models (presented in former Fig. 5 and current supplementary figure 7) aimed at confirming that only the increase of 5HT is responsible for the observed reduction of TH^+ cells. It worked out pretty well, confirming that the effect goes through HTR3A/B and that increase of 5HT is required for the phenotype. Besides, after improved gene expression analysis, we know that "bridge" cells express *Htr3a*, and, thus, the effect goes through them.
2. To be more precise, our single cell data and RNA *in situ* hybridization showed that *Htr3a* receptor is expressed in one cell type - "bridge" cells (with negligible exceptions at E12.5). The new single cell transcriptomics, in-depth analysis of a cell cycle and improved apoptosis study clearly showed that no parameter changes in chromaffin cells, although they reduce their number. Looking at our responses above, the reviewer can see how our updated model works, and that we did not need to reinvestigate the action of pharmacological agents after all the work done with natural ligand (5HT), especially because we obtained fully consistent results.
3. Since all results obtained at E13.5 show the decline of chromaffin cell numbers only in AM, we used it a diagnostic criterion for the pharmacological investigation (done at E13.5) and E13.5 KO embryos. Therefore, it was sufficient to calculate only the numbers of TH^+ cells, as no other cell types show any reliable change at this stage, and would not provide important information for the analysis. Overall, the results of pharmacological study are in agreement with the paracrine regulation, as chromaffin cells are shown in our study to be negative for *Htr3a* expression (combination of SS2 and RNA in situ hybridization), thus specific activation of this receptor can act only on *Htr3a*$^+$ "bridge" cells, which slow down their cell cycle causing slower accumulation of chromaffin cells (appearing as a reduction of chromaffin cells when compared to control).

All in all, the proposed regulatory feedback loop between bridge cells and chromaffin cells is a central part of this study; therefore, it needs to be experimentally substantiated, e.g. by demonstrating 5-HT dependent effects on differentiation of isolated (Htr3aEGFP+/TH-) bridge cells in the absence of 5-HT effects on TH+ chromaffin cells.

1. We appreciate this advice and have tried the culturing of the chromaffin cells and “bridge” cells as explant culture. The pure isolation of “bridge” cells and “chromaffin” cells in sufficient amount for *in vitro* study was not feasible due to the rapid degradation of cell types and their markers. The artificial conditions did not help to answer this question.
2. Instead, we decided to apply the unbiased and systematic method of single cell RNA transcriptomics to disentangle different cell population and to analyze 5HT-dependent effects on differentiation of bioinformatically isolated Htr3a^{EGFP+}/TH^{-} “bridge” cells separately from 5HT effects on TH^{+} chromaffin cells. Bioinformatics-based disentangling allows to observe the changes in different cell populations derived from natural *in vivo* conditions.
As a result, the single cell transcriptomics analysis showed no significant difference in chromaffin TH^{+} population when it comes to gene expression, proportions, compositional effects, cell cycle genes and other features. Basically, the differentiation was unperturbed and the gene expression mechanisms of transition into chromaffin cells did not appear different in control versus the 5HTP treatment. Thus, chromaffin populations did not differ, despite lower chromaffin numbers in treated condition. Logically, this reduction must stem from the upstream events, and the immediate upstream cell population is represented by the “bridge” cells. Although the “bridge” cells did not show any difference in general trajectory and differentiation path, they clearly demonstrated changes in spicing and cell cycle-related genes being differentially spliced in 5HTP treated condition. The “bridge” population was the only population of cells in the entire sympatho-adrenal system that showed any significant difference in gene expression. This nicely coincided with new experimental data, where we measured the cell cycle progression in all sympathy-adrenal population and found that the “bridge” cells have longer cell cycle in 5HTP treated condition. Consequently, the numbers of “bridge” cells were significantly reduced in 5HTP-treated embryos at E12.5 (but not at E13.5), which resulted in a shortage of precursor cells available for differentiation into chromaffin cells. These delayed cell cycle progression and shortage of precursor cells are the reasons for decrease in chromaffin cells numbers. Finally, we found no difference in more advanced apoptosis analysis of all populations. Taken together, these results strongly substantiated our model and supported the role of “bridge” cells in reduced chromaffin cell numbers in 5HTP treated condition. Also, these data support the paracrine mechanisms of signaling via 5HT as the major driver of observed phenotype.

2. Along these lines: does HTR3A protein expression overlap with Htr3aEGFP expression at E13.5 or is EGFP maintained despite downregulated HTR3A protein expression? The latter case would possibly help to support the authors’ model.

To address reviewer’s question, we performed a combination of sensitive fluorescent RNA in situ hybridization against *Htr3a* and *Htr3a*$^{EGFP+}$ expression. *Htr3a*$^{+}$*Htr3a*$^{EGFP+}$ cells were found in the adrenal gland medulla along with *Htr3a*$^{—}$ *Htr3a*$^{EGFP+}$ cells (Supplementary Fig. 2). These new results, indicate that *Htr3a* has a short transient expression in a subset of cells during critical phase before chromaffin cell specification. Many of *Htr3a*$^{EGFP+}$ cells are *Htr3a*$^{-}$ at E13.5, therefore EGFP is detected beyond the expression of *Htr3a* mRNA. Such prolonged

EGFP detection period is due to long time of EGFP degradation. To our regret, these is no reliable antibody against HTR3A that would allow visualization of HTR3A receptor on the cryosections. Even if the presence of HTR3A protein would persist in chromaffin cells, we detect no difference in behavior of this cell population in single cell data or cell cycle analysis or apoptosis analysis. Vast majority of chromaffin cells do not divide at the investigated stages, and only SCPs and "bridge" cell divide. Thus, only the dividing cells can be responsible for the reduction of downstream chromaffin cells, as no apoptosis (and difference in apoptosis) is detected.

3. In Fig. 1, 5-HT expressing cells should be included in the VENN diagrams; in case of paracrine signaling proposed by the authors, there should be little overlap between 5-HT and HTR3A expression. In case of autocrine signaling (my point 1 above), most 5-HT expressing cells would also be positive for Htr3aEGFP.

Yes, we performed the requested analysis, and our new data clearly supports that the paracrine regulation is the main mode of regulation, as chromaffin cells are devoid of *Htr3a* receptor gene expression (please see new Supplementary fig 2b), and only max 5.1% of *Htr3a*$^{EGFP+}$ are 5HT^{+} "bridge" cells (*Htr3a*$^{EGFP+}$TH$^{—}$5HT^{+}). These 5 % of 5HT "bridge" cells can act in an autocrine mode at E12.5-E14.5 in AM. We corrected the manuscript to include this information.

The new Venn diagrams showing 5HT overlap with TH and *Htr3a*$^{EGFP+}$ were added to the Figure 1 (AM) and Supplementary Figure 3 (ZO). Moreover, the proportions of 5HT positive cells among TH^{+} and *Htr3a*$^{EGFP+}$ were included to the figures. Please check new panels d,e,i,j,n,o in Figure 1 (AM) and Supplementary Figure 3 (ZO). These new data presentation allows to clearly see that 5HT-immunopositivity is strongly associated with the onset of TH expression, as mostly TH^{+}*Htr3a*$^{EGFP+}$ early chromaffin cells and TH^{+}*Htr3a*$^{EGFP—}$ mature chromaffin cells are 5HT^{+}.
Since the detection of EGFP was shown to extend beyond the active expression of *Htr3a* mRNA and early chromaffin cells were shown to be largely negative for *Htr3a* mRNA (new Supplementary Fig. 2), we conclude that only TH$^{—}$*Htr3a*$^{EGFP+}$ cells can be referred to as "bridge" cells. We therefore, can not agree that "in case of autocrine signaling (my point 1 above), most 5-HT expressing cells would also be positive for Htr3aEGFP", many TH^{+}*Htr3a*$^{EGFP+}$ early chromaffin cells start being 5HT-immunopositive, but they most likely do not act on themselves as the active expression of *Htr3a* is ceased with the onset of TH^{+} expression.

This conclusion is supported by the new data of cell cycle slowing down in "bridge" cells, but not in chromaffin cells (new Figure 4).

Nevertheless, the careful analysis of data allowed us to find a little overlap of 5HT^{+} expression with TH$^{—}$*Htr3a*$^{EGFP+}$ cells as *Htr3a*$^{EGFP+}$ are presented by 5HT^{+} "bridge" cells (*Htr3a*$^{EGFP+}$TH$^{—}$5HT^{+}) E12.5-E14.5 in AM. Therefore, we modified the proposed mechanism with the notion that that autocrine regulation of minor portion of 5HT^{+} "bridge" cells on "bridge" cells can contribute to the major paracrine mechanism of 5HT-releasing chromaffin cells acting on 5HT-sencitive "bridge" cells.

Please check the updated text:

"Based on this, we propose a new mechanism of a paracrine control, where chromaffin cells release 5HT acting on neighboring "bridge" cells, with some contribution of the autocrine regulation, where few 5HT+ "bridge" cells act on themselves and other HTR3A+ "bridge" cells in their vicinity. As the SCPs and few chromaffin cells express other receptors to 5HT, additional modes of local autocrine and paracrine control might be also present (Fig. 1p)"

4. A key experiment used by the authors to support their model is displayed in Extended Data Fig.5: neither proliferation nor apoptosis seem to be changed upon 5-HTP treatment, supporting the idea that serotonin affects the transition from bridge cells to chromaffin cells. However, given the overall low numbers of proliferative (and apoptotic) cells, proliferation (and ideally apoptosis) has to be quantified in TH+ cells in order to exclude an effect of serotonin on chromaffin cells. "n" might have to be increased to reach statistical significance.

1. We made new experiments assessing proliferation and apoptosis increasing n, and mature chromaffin cells neither divide nor die (with negligible exception in consistency with the earlier version of the manuscript).
2. Furthermore, we principally improved the analysis of proliferation in different population of cells building AM and ZO during the revision. To be more specific, we applied double thymidine labeling (EdU and CldU) at a greater concentration then in the previous version of the manuscript and at shorter time window. This allowed us, not only to calculate the overall proportion of proliferating cells, but also assess the length of cell cycle in different populations of cells. The new results showed that the cell cycle progression was significantly slowed down in "bridge" cells in AM and in SCPs in ZO. At the same time, the overall "proliferation" (cells incorporated at least one of the thymidine analogues) of all cell populations in AM and ZO was not altered by the 5HTP treatment. Please, check the plots on new Figure 4b and new Supplementary Figure 6c. Please, see the new Figure 4 and Supplementary Figure 6 and the detailed answer to the question 1 point 2.
3. As for cleaved Caspase 3 immunostaining, we reanalyzed out data and we added more embryos to the control and treatment group to increase the power of the analysis. In the revised version of the experiment presented now in the new Fig.4, panel e, 5 embryos per condition were analyzed. Virtually no cleaved CASP3+ cells were found in both AM and ZO, as there was no apoptosis enhancement due to 5HTP treatment in chromaffin organs.

5. Based on Fig. 1, most Htr3a^{EGFP+} cells co-express TH at E13.5. How can the authors explain, therefore that in the vitro experiment described in Fig. 4, Htr3aEGFP+ but not TH is reduced? The authors should exclude that 5-HT influences transcription/expression of Htr3aEGFP+ rather than cell numbers. For the quantification of these data, I feel that counting numbers of marker-positive cells, rather than measuring the volume of 3D reconstructed images would be more accurate.
In line 236-237, it is written that the TH+ structures did not differ across all experimental conditions. This is surprising, as in vivo the adrenal glands are smaller following 5-HT treatment. It would be good to comment on this discrepancy here.

During the revision process we carefully analyzed the conditions of *in vitro* experiment, and we decided that due to a highly artificial environment of explant culture, increased cell death and other issues, the observation does not help to clarify the mechanism of 5HT mediated reduction of chromaffin cell numbers. Therefore, the figure 4 and corresponding part of the manuscript was removed as technically poor and being heavily criticized by our colleagues.

6. The statistical analysis should be described in the Materials and Methods section. Importantly, a standard error of mean (SEM) cannot be calculated with only three animals, this should be corrected and for those experiments, for which the numbers cannot be increased, the standard deviation has to be shown.

We agree, and for all experiments where only 3 data points are available the SD is shown instead of SEM. Alternatively, the violin plots are shown with all the data points along with median and quartiles.

The statistical analysis is now described in detail in material and method section. Please check

"Statistical analysis was done using GraphPad Prism 8.1.1 software. All datasets were checked for normality with Shapiro-Wilk test, as this test reliably works for small datasets. For datasets with normal distribution double sided unpaired t-test was applied. For datasets which failed a test for normal distribution, Mann-Whitney test was applied. For comparisons between multiple groups for one variable the one-way ANOVA test was applied (Fig. 3d, Fig. 6a,c,l, Supplementary fig. 7 d,e,f). In case one-way ANOVA test was showing significant differences, Tukey's multiple comparison test was used for a robust pairwise comparison of groups with unequal size (every mean to every other mean) or Dunnet's multiple comparison test for a pairwise comparison of groups to a control group.

In the analyses of statistical significance in catecholamine measurement as well as in behavioral tests (Fig. 7, Supplementary Fig.8) we have chosen the pairwise comparison within genders. The reason for it is that the treatment with 5HTP or 1 hour restrain was applied at the stage, when the sexual dimorphism is not established in the forming adrenal glands and, consequently, the treatment equally affects males and females. However, adrenal glands have sexual dimorphism at postnatal stages and therefore must be analyzed separately, since the combined analysis of these groups can mask the effects of our prenatal treatment. We have calculated statistical significance with Mann-Whitney test within genders between treated and control animals as this test as this test is applicable to small samples and can evaluate statistical significance regardless of the normality of data distribution and homoscedasticity of the data. For these experiments the analysis was performed in SigmaPlot 12.1"

7. In Figure 2d, area measurements are shown. However, according to the text, measurements of cortical thickness have been performed. This needs to be clarified.

We thank the reviewer for spotting this inconsistency. We now corrected this situation as the area of cortex was measured by means of subtracting the TH^+ area from the overall area of adrenal gland section.

The appropriate changes are introduced in the text:

"Immunohistochemical analysis of adrenal glands of littermates revealed a significant reduction of the AM, whereas the area of the adrenal cortex was similar in control and experimental offspring (Fig. 2g)"

and in the material and method section:

"The area of adrenal gland section was calculated by surrounding the area based on DAPI signal. The area of medulla was calculate based on TH^+ signal within adrenal gland. The area of cortex was calculated by subtraction of adrenal medulla area from the area of the whole gland per individual section. 3 section per gland and 2 glands per embryo were evaluated."

8. In Figure 6, a difference in the response between genders is shown. Why could that be? This should be discussed here or in the discussion.
Importantly, in this figure, the behavioral studies show barely any difference across the conditions. The authors should show here the single data points and assess statistical significance of the data.

1. Rodent males and females respond to a stress differently, and, because the sympatho-adrenal system in involved in the stress response, we observe sexually dimorphic results of our tests.

2. Next, during this revision, we substantially improved the behavioral studies to clarify the significance of the phenotypes. We now employed two additional test that were designed to assess the aggressive behavior. Those test are done exclusively on males and our results show a clear cut behavioral changes in 5HTP prenatally treated males. In the resident-intruder test and the foot shock induced aggression test, 5HTP prenatally treated males showed no aggressive behavior in comparison with control males. This sharp behavioral changes are direct consequences of the reduced overall adrenaline supply for the organism measured in ng/organ. Please see the updated Figure 7.

Following the reviewer's recommendation we now present all the behavioral data as violin plots with individual data points, which clearly show the distribution of all data points, along with median and quartiles. We also performed better statistical analysis of all the data and now clearly indicate the statistical test used and the *p* values in the figure legends.

3. When it comes to the tests performed previously (currently all the data is summarized in Supplementary Fig. 9), all of these tests are designed to assess similar behavioral patterns – flexibility and adaptability in the unknown environment. Even though males and females have performed differently in individual test the overall conclusion is not different between the genders. Both

males and females, when prenatally exposed to the elevated levels of serotonin change their behavioral patterns toward more reactive and adaptive to the unknown environments. It is well known that the morphology and functions of the adrenal glands, as well as the response to stress, are characterized by sexual dimorphism (Hinojosa-Laborde et al., 1999; Lundberg 2005; Levasseur et al., 2019; Wakil et al., 2013; Bastida et al., 2007), which may be reflected in the differences in performances in individual tests.

Please, check the improved text:

"Moreover, the experimental animals turned out to be more adaptive and flexible, demonstrating less anxiety and reduced stress-induced behavior (Supplementary Fig. 9d). These results are in line with in vivo measurements of catecholamines in adult mice revealing lower levels of adrenaline supplied by smaller adrenal glands (evident when measured as ng/organ, Fig. 7c) in animals prenatally exposed to the elevated 5HT levels."

4. Finally, to place our test results into the more natural context and to assess how different coping strategies correlate with the adrenal size, we performed an expedition to Siberia and studied the wild rodent population. Indeed, the concept of coping strategies is often applied to the analysis of population dynamics in wild rodents. It is well known that colonies of wild voles have "cycling" structure with the periods of colony size growth changing with the periods of extensive migration aiming to occupy new territories and reduce the pressure of overpopulations. From previous studies we know that coping strategies of the animals that emigrate and animals that reside in the original areal are different. To test how this corresponds to the size of adrenal medullae, we collected adrenal glands from the resident and migrant wild red-backed voles during the year of peak migration (2020). The comparison of relative adrenal medulla size between the animals in "resident" and "migrant" groups showed that the "migrant" animals had significantly smaller medullas in adrenal glands.

5. This observation ties together the results of experiments presented in the revised manuscript and places the observed connection between elevated levels of maternal serotonin and the size of adrenals in progeny into the new ecological perspective. Elevated levels of serotonin in mothers lead to the reduction of chromaffin cells in progeny, which is reflected in hormonal balance of adult progeny and changes in their behavioral patterns. Furthermore, we report similar behavioral phenotype in progeny after the simulated mild stress in pregnant laboratory animals (new Fig. 6 e,f). The increased population density of wild rodents is associated with increased levels of stress, which leads to the increase in 5HT (as known from the literature and our own new data). This might, in turn, result in the birth of a generation, which is capable of exploring new territories due to their altered hormonal balance and more adaptive behavior. Therefore, it is reasonable to speculate that observed 5HT-HTR3A link might be acting as a way of prenatal programing of offspring with more flexible and adaptive coping strategy to allow for colony segregation on "residents" and "migrants" necessary to maintenance of the appropriate colony size.

9. The link between the study on HTR3A during chromaffin cell development and the tumorigenesis experiments presented in Figure 7 is poorly established. First, the association of $HTR3A^{high}$ expression and tumorigenesis is purely correlative; the cell lines could obviously carry different mutations, etc that may well explain their differential behavior independently of serotonin signaling.

1. Htr3a is expressed by “bridge” cells, the progenitors of chromaffin cells. During this revision, several strong manuscripts were published that strongly support the connection of Htr3a+ “bridge” cells and neuroblastoma based on the human-specific single cell transcriptomics data comparisons. For instance, please see Jansky et al. Nat Genetics 2021. To be more detailed, the Jansky et. al. team showed that some neuroblastoma tumors contain malignant subpopulations, which are transcriptionally most similar to the human embryonic “bridge” cells, which hints toward the reuse of the “bridge” cells transcriptional program in specific NB tumors or suggests the origin of these tumors from “bridge” cells (immediate precursors of chromaffin cells). Please also see Kameneva et al., Nature Genetics 2021 for the specifics of human sympatho-adrenal development.

2. Next, we agree with the reviewer that the tumor cells and cell lines carry different mutations, and may show differential behavior independently of serotonin signaling. To accommodate this comment, we introduced the following passage into our text:

   “Still, it might be wise to keep in mind the potential difference between tumor and healthy $HTR3A^{+}$ “bridge” cells, as the tumor cells might have additional, unpredictable effects following from HTR3A activation.”

3. Although we agree that the observations made regarding the tumorigenicity of $HTR3A^{high}$ neuroblastoma cells are correlative to a large extent, we have now included additional experiments and two more neuroblastoma cell lines, CHLA-15 and CHLA-20, further strengthening our conclusions.

   Both CHLA-15 and CHLA-20 were used as $HTR3A^{high}$ models as indicated by HTR3A mRNA (Fig. 6a) and protein (Fig. 6b,c) levels. For consistency matters, all analyses presented in Fig. 6 were performed with cells from newly thawed cell stocks (authenticated by STR profiling) and replace the previous data. Of note, in contrast to the previous manuscript version, the newly thawed patient-derived NBL-40 neuroblastoma cells (several different stocks/passages tested) exhibited low expression of HTR3A. However, this is in line with the response of these particular NBL-40 cells to HTR3A agonist N-methylquipazine dimaleate (NMQ) (Fig. 6d) as well as with the low expression of proteins associated with aggressive neuroblastoma (Fig. 6b). To avoid any uncertainty, we decided to utilize CHLA-20 established neuroblastoma cells as a $HTR3A^{high}$ model in functional assays (Fig. 6h-k).

   Therefore, we now show several findings supporting our conclusion that $HTR3A^{high}$ expression (but not the receptor activity) is associated with aggressive neuroblastoma growth: 1) both $HTR3A^{high}$ cell lines tested, SH-SY5Y and

CHLA-20, are tumorigenic (Fig. 6j-k) and form significantly more spheres (a proxy for tumor-initiating capacity, Fig. 6i) than HTR3A^{low} NBL-28 and NBL-38 cells; 2) cell lines that express high levels of HTR3A also express markedly higher levels of major drivers of aggressive neuroblastomas, N-MYC and c-MYC, or one of the core stemness factors, SOX2 (Fig. 6b); 3) HTR3A agonists dramatically limit cell proliferation in all HTR3A^{high} cell lines but they do not affect HTR3A^{low} cell lines, or they show effects at much higher doses (Fig. 6d,e); 4) NMQ does not induce differentiation of neuroblastoma cells as no reduction of sphere-forming capacity was observed after the treatment with HTR3A agonist NMQ (Fig. 6i), which also did not induce apoptosis in HTR3A^{high} cells (Supplementary Fig. 8).

Thus, in line with our findings that stimulation of HTR3A slows down cell cycle in “bridge” cells (Fig. 4c), we demonstrate that HTR3A^{high} neuroblastoma cells resemble less differentiated cells, which show cancer stem cell features and rapidly reduce their proliferation in response to HTR3A agonists. It is important to note that we do not propose HTR3A as a functional regulator of neuroblastoma stemness but rather as a potential marker of less differentiated neuroblastomas.

To substantiate these data, the authors need to perform functional experiments (e.g. CRISPR knockout of Htr3a in the high expressing cells lines) in order to show that loss of tumor growth is indeed due to reduced expression of this receptor.

To address this comment, we employed CRISPR to knockout HTR3A in HTR3A^{high} SH-SY5Y. We chose ribonucleoprotein (RNP)-mediated CRISPR using complexes of Cas9-GFP (CAS9GFPPRO; Sigma-Aldrich) and one of the following synthetic sgRNAs (all Sigma-Aldrich; MISSION™ gRNA IDs shown in brackets): HTR3A-specific sgHTR3A#1 (HSPD0000020297), sgHTR3A#2 (HSPD0000020295), and sgHTR3A#3 (HSPD0000020298); negative control sgNC1 (NegativeControl1), and sgNC2 (NegativeControl2); and a positive control sgAAVS1 (HScontrol_AAVS1) recognizing adeno-associated virus integration site 1. After lipofection of cells (Lipofectamine CRISPRMAX Cas9 Transfection Reagent; Thermo Fisher Scientific) with respective RNP particles, GFP-high cells were sorted by FACS, both as individual clones (1 cell per well of 96-well plate; 192 wells in total for each sgRNA) and a pool of transfected cells. However, after 3 months of expansion, similar level of HTR3A protein was detected in all growing clones, regardless the sgRNA used. Although a minor clone-to-clone variability was observed, the HTR3A expression in individual clones or pools was always much higher compared with the HTR3A^{low} NBL-38 control samples. Although we cannot fully exclude the possibility that CRISPR knockout was unsuccessful, given the intrinsic cell heterogeneity of SH-SY5Y (Ferlemann. et al., Sci Rep 2017, 7(1):13612. doi: 10.1038/s41598-017-13497-8; Mercatelli et al., Methods Protoc. 2021, 4(2):28. doi: 10.3390/mps4020028) and the number of isolated individual FACS-sorted cells tested, we conclude that only cells that maintained high HTR3A expression were able to self-renew and grow enough to establish a clonal cell line. Again, this is in line with our sphere assay results demonstrating that HTR3A^{low} cell lines comprise very low percentage of cells able to self-renew enough to form neurospheres (Fig. 6i).

Moreover, they should analyse the differentiation status of the neuroblastomas in order to strengthen their hypothesis that 5-HT treatment affects differentiation of bridge cell-like cells in the tumors. Do such cells exist in neuroblastoma?

Indeed, recent single-cell transcriptomic analyses by Jansky et al. (Nat Genet 53, 683–693 (2021). https://doi.org/10.1038/s41588-021-00806-1) demonstrate that high-risk neuroblastomas and neuroblastoma cell lines comprise cells with the "bridge" cell transcription signature.

However, by our detailed analysis of cell cycle progression using dual labeling with thymidine analogs we now show that 5HT treatment does not inhibit differentiation of "bridge" cells into chromaffin cells *per se* but slows down cell cycle in "bridge" cells (Fig. 4b,c) which results in the observed effects. Similarly, inhibition of proliferation but not induction of differentiation was observed in neuroblastoma cells after treatment with HTR3A agonists (Fig. 6d,e,i). As demonstrated by limiting dilution sphere formation assay, a well-accepted in vitro test of undifferentiated/stem-like cells, increased HTR3A activity does not affect differentiation of HTR3A^{high} neuroblastoma cell lines. Both N-methylquipazine (NMQ)-pretreated and vehicle-pretreated neuroblastoma cells maintained similar sphere formation capacity (Fig. 6i).

Thus, we now clearly state this in the Results section:

"Therefore, the activation of HTR3A receptor does not compromise the stem-like state of neuroblastoma HTR3Ahigh cells, but only reduces their proliferation.".

In the Discussion, the authors refer to cancer stem cell properties; are HTR3Ahigh cells "de-differentiated" (e.g. TH-negative) and exhibit cancer stem cell properties? Furthermore, they should comment on whether there is a correlation between reduced cell proliferation and differentiation status. Are differentiated cells less proliferative and, thus, less tumorigenic?

Following the response to your previous comment, we now present results of two most complex *in vitro* and *in vivo* functional assays of cancer stem cells – limiting dilution sphere assay (Fig. 6h,i) and tumorigenicity assay in NSG mice (Fig. 6j-k). In both assays, HTR3A^{high} neuroblastoma cell lines show markedly enhanced cancer stem cell characteristics compared with HTR3A^{low} cell lines. Thus, we conclude that HTR3A expression associates with the aggressive phenotype of neuroblastoma cells. Again, it is important to note that we do not suggest anywhere in the manuscript that HTR3A is a driver of this aggressive phenotype. On the contrary, based on the HTR3A activity-dependent inhibition of cell proliferation, we might speculate that upregulated HTR3A expression is rather a bystander effect in poorly differentiated/high-risk neuroblastomas that evaded 5-HT-mediated control.

As stated above, treatment with HTR3A agonists significantly inhibits proliferation of HTR3A^{high} neuroblastoma cells (Fig. 6d,e) but it does not induce their differentiation. By pre-treating cells with 75 µM NMQ for 5 days prior sphere assay, we demonstrate that activation of HTR3A receptor does not compromise the stem-like state of neuroblastoma HTR3A^{high} cells, but only

reduced their proliferation during the treatment period (Fig. 6i). Overall, HTR3A^{high} neuroblastoma cell lines appear to be more de-differentiated, but the differentiation status is not under control of HTR3A.

Of course, terminally differentiated cells do not proliferate. However, it is otherwise very hard to correlate differentiation and proliferation, especially in cancer that breaches developmental paradigms. Generally, stem cells/cancer stem cells can switch between quiescent and active proliferative states and may divide symmetrically and asymmetrically. Thus, the very same undifferentiated (stem, progenitor, cancer stem) cell may, depending on the conditions, proliferate very rapidly and/or symmetrically, undergo slow cell cycles producing a rapidly short-term proliferating but more differentiated progeny, or it does not divide at all and remains quiescent.

Nevertheless, in the revised manuscript we demonstrate that HTR3A activation serves as an intrinsic constraint in cell proliferation in both “bridge” cell population and HTR3A^{high} neuroblastoma cells.

Finally, on line 311, the authors state that the reduced proliferation of the HTR3Ahigh expressing neuroblastoma following 5-HT treatment correlates with their in vivo mouse data. However, they showed before that there is no detectable difference in proliferation in neither SCPs, bridge and chromaffin cells following 5-HT treatment in vivo (Extended Data Figure 5). This discrepancy needs to be further clarified.

By analyzing the effects of 5HTP on cell cycle progression of “bridge” cells using dual labeling with thymidine analogs during chromaffin cell development, we now show that activation of HTR3A slows down the cell cycle specifically in “bridge” cells (Fig. 6b,c) which then results in the reduction of adrenal medulla growth (Fig. 3). In ZO the cell cycle progression was slowdown in SCPs. Similarly, significantly reduced cell proliferation was observed in HTR3A^{high} neuroblastoma cells following the treatment with HTR3A agonists (Fig. 6d,e). Thus, these results are completely in line and suggest a common role of 5-HT/HTR3A feedback loop in limiting the cell cycle progression.

Minor points:

The main and supplementary figures should be numbered.

All main and supplementary figure are numbered in the figure legends.

In general, in the figure legends, the arrowheads should be explained.

All arrowheads are explained.

In Figure 3f, a statistical analysis should be performed, as done in all the other graphs shown in this figure.

For a former Fig. 3f (current Fig.3d) one-way ANOVA is used to assess statistical significance.

In Figure 4c, the dot plot symbols in the different conditions are barely visible, the size of the graph needs to be increased.

The figure 4c is removed from the revised version of the manuscript. Nevertheless, we controlled that the data points are visible in all the revised figures.

In the Extended Data Figure S6b and S6d, the y-axis should be labelled.

The former Extended Data Figure S6b and S6d (currently Supplementary Figure 7b) has now y-axis labelled.

In Figure 6a, catecholamine is misspelled, this should be corrected.

It is corrected

In Figure 6c, other names are used for the catecholamines tested than in the text of the paper. This should be corrected and misspellings should be taken care of.

The names of all catecholamines and their metabolites are unified in the text and in the figures.

On lane 286, Figure 6e is referenced wrongly, it should be 6c.

This is corrected.

1. de Boer, S.F., Buwalda, B. & Koolhaas, J.M. Untangling the neurobiology of coping styles in rodents: Towards neural mechanisms underlying individual differences in disease susceptibility. *Neurosci Biobehav Rev* **74**, 401-422 (2017).
2. de Boer, S.F., Olivier, B., Veening, J. & Koolhaas, J.M. The neurobiology of offensive aggression: Revealing a modular view. *Physiology & Behavior* **146**, 111-127 (2015).
3. Furlan, A. *et al.* Multipotent peripheral glial cells generate neuroendocrine cells of the adrenal medulla. *Science* **357** (2017).
4. Ritter, K.E. & Southard-Smith, E.M. Dynamic Expression of Serotonin Receptor 5-HT3A in Developing Sensory Innervation of the Lower Urinary Tract. *Frontiers in Neuroscience* **10** (2017).
5. Ritter, K.E., Wang, Z., Vezina, C.M., Bjorling, D.E. & Southard-Smith, E.M. Serotonin Receptor 5-HT3A Affects Development of Bladder Innervation and Urinary Bladder Function. *Front Neurosci* **11**, 690 (2017).
6. Cote, F. *et al.* Maternal serotonin is crucial for murine embryonic development. *PNAS* (2006).
7. Alenina, N. *et al.* Growth retardation and altered autonomic control in mice lacking brain serotonin. *PNAS* (2009).
8. Lynn-Bullock, C.P., Welshhans, K., Pallas, S.L. & Katz, P.S. The effect of oral 5-HTP administration on 5-HTP and 5-HT immunoreactivity in monoaminergic brain regions of rats. *J Chem Neuroanat* **27**, 129-138 (2004).
9. Magnussen, I., Jensen, T.S., Rand, J.H. & Van Woert, M.H. Plasma accumulation of metabolism of orally administered single dose L-5-hydroxytryptophan in man. *Acta Pharmacol Toxicol (Copenh)* **49**, 184-189 (1981).

10. Finger, T.E., Bartel, D.L., Shultz, N., Goodson, N.B. & Greer, C.A. 5HTR3A-driven GFP labels immature olfactory sensory neurons. *J Comp Neurol* **525**, 1743-1755 (2017).

REVIEWER COMMENTS

Reviewer #1 (Remarks to the Author):

The authors have revised the manuscript according to my comments and recommendations. They have provided an extensive revision which includes pertinent experiments with meaningful findings. I feel this revision is well done though it admittedly has limitations.

I do have a minor comment regarding catecholamine turnover. This relates to the nature of catecholamine and metanephrine concentrations. Although levels of metanephrines depend on catecholamine production, these levels are also related to subsequent metabolism. This metabolism occurs not only via COMT but also via MAO. Thus, we cannot assume that high metanephrine levels always reflect high catecholamine synthesis...e.g. inhibition of MAO may also lead to consequent elevated levels of catecholamines/metanephrines. Therefore, the authors must be careful in their interpretations/conclusions. I would suggest this consideration should be addressed in the event the manuscript requires further minor revisions based on my comments.

Reviewer #2 (Remarks to the Author):

The authors have added new supporting data that convincingly address most of my prior concerns.

The manuscript would be strengthened by:

1. L180: refer to mature chromaffin cells as TH+ chromatin cells.
2. Align all text with the correct figures and legends (i.e. L183 refers to the incorrect insets).
3. The first results section fits with the proposal that paracrine 5HT may act on neighbouring bridge cells which is also referred to in discussion. The mouse data in which 5HT signalling is removed shows that paracrine signalling has no major effect and this should be worded accordingly in both the results and discussion.
4. L201-205: This is hard to understand and not clearly depicted in the figure. The proportion of Sox10+/Hrt3a+ cells does not look to decrease in the Venn diagrams, rather Sox10+ cells decrease in total.
5. A discussion of why bridge cells are reduced at E12.5 and not E13.5 is needed.
6. The analysis of cells remaining in the cell cycle is a welcome addition to the manuscript. This shows an increase in double +ve cells which could be taken to suggest that cell cycle length is expanded (not expended) in 5HT treated mice. To help the reader it would be good to state that double +ve cells are suggestive of increased cell cycle length.
7. Typo in Supp Fig 1C
8. Consider replacing Fig 4F - why is a pressure gauge used?
9. Consider representing the qRT-PCR of Fig 6a as per 6c.

Reviewer #3 (Remarks to the Author):

The authors have considerably improved their manuscript "Serotonin limits generation of chromaffin cells during adrenal organ development". The major revision add substantial mechanistic evidence for the role of 5-HT on bridge cell cycle progression and the regulation of the chromaffin organ size and development. This work contributes important and novel information of relevance to multiple fields including psychiatry and oncology. The authors did a good job in measuring 5-HT levels in 5-HTP treated dams. The addition of aggression and exploratory behavior strengthens the manuscript and the idea that increased 5-HT levels lead to decreased medulla size which ultimately correlates with more explorative and less aggressive behaviors. The work is clearly presented and of high quality, but I still have a final major request regarding the supplementary behavioral data:

1. The authors refer to supplementary figure 9 and state the following: "As the elevation of normetanephrine is not observed in females, this might signify the sexual dimorphism in endocrine metabolic systems and might explain the behavioral differences observed in males and females."(lines 413-415). They added: "the observed differences in males and females, as well as the only partial correlations with behavioral data might be due to other systemic regulatory mechanisms, which are not covered by this study." (Lines 417-419). If the authors want to use this sentence, they need to perform a 2-way analysis. As it is right now a between treatment within sexes Mann Whitney test has been performed; however, the sex variable or interaction effect of treatment x sex is not reported nor tested. Thus, these two sentences are without any statistical support. (I suggest removing the data or not even discussing the sexual dimorphism but rather state the actual limitation of these results in detecting a difference. Or the authors can just state that for each individual test they detected a difference in one of the sexes but not draw any conclusion on sexual dimorphism as there is none tested.)

2. The authors still need to report the number of litters used and discuss the use of mouse versus litter as the treatment unit (N)

Reviewer #4 (Remarks to the Author):

Kameneva and colleagues have made major efforts to address our various concerns by performing several new experiments, including very comprehensive studies involving SmartSeq2 analysis and validation by RNA scope. This allowed the authors to better define SCP-derived 'bridge cells' and further strengthen their model on a regulatory feedback loop controlling the generation of chromaffin cells. Interestingly, according to their new data, genes potentially involved in regulating cell cycle length were found to be differentially spliced in the bridge cells. This finding as such is highly interesting and could be addressed in future experiments.
Likewise, the link of their data from developmental processes to neuroblastoma formation has been substantiated by new experiments and by reference to most recently published findings by others that support the authors' conclusion.
I appreciate that the authors very carefully addressed our comments in detail and feel that the study has been greatly improved.

There is only one issue that the authors should comment on: they observed less numbers of bridge cells upon 5HTP treatment in their cell cycle dynamics experiment, whereas no reduction can be seen in Figure 3. Why is this? They should discuss this discrepancy as one would expect to see a reduction in bridge cells in the experiments shown in Figure 3 as well.

Reviewer #5 (Remarks to the Author):

This is a lovely developmental biology paper, which deserves to be published. However, it does have two major issues:
- The experimental evidence for the 5HT mediated mechanism for suppressing chromaffin cell production relies entirely on animal experiments, for obvious reasons. However, there is no serious attempt to check if human data is consistent with the story they present. For example, a superficial check of the numerous single cell fetal adrenal atlases suggest that in humans, HTR3A is not expressed in bridge cells. Can feedback from 5HT still be the mechanism for regulating chromaffin cell production in humans given this? It is not clear to me that it can.
There is good reason to expect that human adrenal glands will differ from mouse. Recent single cell studies of the human adrenal gland and neuroblastoma (see Dong et al. Cancer Cell, 2020 and subsequent commentary) have demonstrated that human and mouse adrenal glands differ with respect to the key genes they express.
To me there is a section missing that connect the mechanism they have established in rodents to what is known about the expression of the key genes (such as HTR3A) in the human adrenal medulla.
- On a somewhat related note the section on neuroblastoma is not very compelling. I do not think it would should be removed entirely, but the commentary surrounding it should be toned down. I would strongly encourage the removal of the speculation regarding the origins of neuroblastoma. In particular these statements should be removed:
- "These are key characteristics of cancer stem cells, and this observation may point to the previously unrecognized cell states responsible for tumor aggressiveness, in line with recent findings of similarity of genetic profiles of HTR3A+ "bridge" cells and neuroblastoma subpopulations8-10."
- "In line with this reasoning, recent studies showed that the initial stages of chromaffin cell development depend on the recruitment of local nerve-associated Schwann cell precursors (SCPs), which turn into a short-living transient population of "bridge" cells that rapidly transitions towards mature chromaffin cells in mouse and human embryos 6-8. This finding complicated the old picture of the adrenal development (where migratory neural crest cells immediately generate chromaffin tissues), and raised a series of questions regarding the control of the number of chromaffin progenitors operating during the differentiation steps vulnerable to initiation of sympathoadrenal tumors (neuroblastoma, paraganglioma and pheochromocytoma). Moreover, another recent study suggested the involvement of "bridge" chromaffin progenitors in neuroblastoma initiation 9, 10 . These "bridge" cells are characterized by the expression of HTR3A – a co-receptor to serotonin 5-hydroxytryptamine (5HT). Based on that, 5HT was recently hinted to be a part of the mechanism related to the development of adrenal medulla 6.
- Really all the data show is that there are a subset of neuroblastoma cell lines that express HTR3A and respond to HTR3A agonists. The origins of neuroblastoma are debated (to put it mildly) and the fact that this one gene is expressed on a subset of cell lines does not add productively to this debate. Even if they were to discuss the origins of neuroblastoma (which they should not given their data), the expression of HTR3A is more consistent with a neuroblastic cell of origin given that HTR3A is most strongly expressed by neuroblastic cells, not bridge cells, in the human fetal adrenal medulla (see above, Janksy et al 2021). That neuroblastomas mostly resemble neuroblastic cells is also the consensus from all the recent single cell neuroblastoma studies.

REVIEWER COMMENTS

<u>Reviewer #1 (Remarks to the Author):</u>

The authors have revised the manuscript according to my comments and recommendations. They have provided an extensive revision which includes pertinent experiments with meaningful findings. I feel this revision is well done though it admittedly has limitations.

I do have a minor comment regarding catecholamine turnover. This relates to the nature of catecholamine and metanephrine concentrations. Although levels of metanephrines depend on catecholamine production, these levels are also related to subsequent metabolism. This metabolism occurs not only via COMT but also via MAO. Thus, we cannot assume that high metanephrine levels always reflect high catecholamine synthesis. e.g. inhibition of MAO may also lead to consequent elevated levels of catecholamines/metanephrines. Therefore, the authors must be careful in their interpretations/conclusions. I would suggest this consideration should be addressed in the event the manuscript requires further minor revisions based on my comments.

We are thankful for reviewer's comment and we replaced our interpretation/conclusion for the one including more careful discussion involving MAO in addition to COMT.

Please see the updated text of the result section:

"Of note, high metanephrine levels might not always reflect high catecholamine synthesis and following catabolism, as inhibition of monoamine oxidase may also lead to elevated levels of catecholamines/metanephrines, which we cannot rule out even though we consider this scenario unlikely based on the previous literature [48-51]. "

"To ensure that the observed reduction of adrenaline in males and the reduction of adrenaline, noradrenaline and dopamine in females were not associated with accelerated catabolism by catechol-O-methyltransferase in blood, we measured catecholamine metabolites: metanephrine and normetanephrine (Supplementary Fig. 10c)"

The logic of our interpretation and the choice of catecholamine degradation metabolites is based on the following arguments: among catecholamine degradation enzymes COMT is known to be predominantly active in the general circulation (Axelrod, 1937; Kopin, 1964.). COMT acts on catecholamines mainly outside the cells, whereas MAO is localized mainly within the cells. Intraperitoneal or intravenous administration of adrenaline and noradrenaline revealed that metanephrine and normetanephrine were the major metabolites in plasma and urine following the specific activity of COMT. Thus, according to the literature, we believe that the contribution of MAO's role in this process is negligible.

We took into account the data from the following publications:
Julius Axelrod Noradrenaline Fate and Control of Its Biosynthesis. Science, New Series, Vol. 173, No. 3997 (Aug. 13, 1971), pp. 598-606
Axelrod J. Metabolism of epinephrine and other sympathomimetic amines. Phys.Rev. 1959, v. 39(4), 751-776.
Kopin I.J. Storage and metabolism of catecholamines: The role of monoamine oxidase. Pharmacol.Rev. 1964, 16, 179-191
H Parvez, G Ismahan, S Parvez, M B Youdim. Developmental changes in the activity of catechol-O-methyl transferase in rat and rabbit fetuses/ J Neural Transm. 1979;44(1-2):65-75. doi: 10.1007/BF01252702.

Reviewer #2 (Remarks to the Author):

The authors have added new supporting data that convincingly address most of my prior concerns. The manuscript would be strengthened by:

1. L180: refer to mature chromaffin cells as TH+ chromatin cells.

We improved the text mentioning the molecular marker (TH) according to the reviewer's recommendation.

Please check the updated text:

"Indeed, TH—/Htr3a^{EGFP+} "bridge" cells compose most of the AM and ZO at E12.5 (Fig. 1a-d, Supplementary Fig. 4a-d), and they rapidly differentiate into TH+/Htr3aEGFP+ early chromaffin cells and TH^{+}/Htr3a$^{EGFP—}$ mature chromaffin cells, composing most of the AM and ZO at E13.5 and E14.5 (Fig. 1f-o, Supplementary Fig. 4f-o)."

2. Align all text with the correct figures and legends (i.e. L183 refers to the incorrect insets).

We double-checked all the references to the figures, introduced necessary changes, and now they appear correct.

3. The first results section fits with the proposal that paracrine 5HT may act on neighboring bridge cells which is also referred to in discussion. The mouse data in which 5HT signaling is removed shows that paracrine signaling has no major effect and this should be worded accordingly in both the results and discussion.

We agree with the reviewer and we added the following clarification to the Results:

"As the elevated levels of 5HT lead to a decreased chromaffin cell numbers in vivo by delaying the cell cycle of precursor "bridge" cells, we expected to see the opposite effect in the case of reduced levels of 5HT. However, the previously reported genetic loss-of-function of the HTR3A receptor failed to show any abnormal phenotype in adrenal glands [43]. A potential explanation for these observations is that 5HT affects cell cycle progression in HTR3A^{+} "bridge" cells only when 5HT levels reach a certain threshold. Furthermore, HTR3A-dependent paracrine regulation may not be critical for the development of the adrenal gland, but is an important controller of excessive chromaffin tissue growth and pre-malignant states."

Discussion:

"The proposed mechanism of chromaffin cell number control via 5HT-HTR3A-dependent paracrine regulation is unidirectional, as the complete elimination of 5HT and pharmacological blockage of HTR3A receptor do not result in overgrowth of chromaffin cell organs"

4. L201-205: This is hard to understand and not clearly depicted in the figure. The proportion of Sox10+/Hrt3a+ cells does not look to decrease in the Venn diagrams, rather Sox10+ cells decrease in total.

The area of the overlap between Sox10 and Htr3a cells is smaller at E14.5 in adrenal gland in comparison with E12.5 and E13.5 stages, and the difference between the stages might be hard to spot. Therefore, we additionally provided the magnified views of Sox10^{+}

cells that lost EGFP immunopositivity in Fig. 1i insets and the percentage of $SOX10^+$/*Htr3a*$^{EGFP+}$ cells at E13.5 and E14.5.

In addition, we rephrased the results:

"When the differentiation of chromaffin cells slows down around E14.5 (E13.5 in ZO)[6, 8]*, the proportion of $SOX10^+$/Htr3a^{EGFP+} cells decreases gradually (from 6.3% at E13.5 to 4.7% at E14.5), as the $SOX10^+$ SCPs engage into the "bridge" fate at a decreasing rate. (Fig. 1k-o, Supplementary Fig. 4f-o)."*

5. A discussion of why bridge cells are reduced at E12.5 and not E13.5 is needed.

The difference in bridge cell numbers at E12.5 and E13.5 can be explained by (1) the time when the samples were analysed after 5HTP injection because of (2) rapid 5HTP and 5HT reduction (pharmacokinetics) after reaching their peak due to injection, and (3) the speed of bridge cell turnover and differentiation into chromaffin cells (La Manno et al., 2018, Supplementary note 2, Section 6). In the experiment addressing cell cycle dynamics, the analysis of cell populations was done 8 hours after the 5HTP injection. In the analysis of cell populations at E13.5, cell counts were done at 24 hours after the injection. Soon upon the injection of 5HTP, the currently existing bridge cells become affected, and the resulting chromaffin cells generated within 14 hours-time-window appear reduced in their numbers. However, the process of the recruitment of the new bridge cells from SCPs is not affected or stopped. 5HTP, being injected at E12.5, and the corresponding elevation of 5HT are likely weakened by the time when the new bridge cells are recruited during later stages. By E13.5, new bridge cells are recruited from SCPs and those are not that much affected by the transient 5HTP and 5HT elevation, and their numbers appear similar in treatment vs control at E13.5. Still, the earlier, near-immediate defects in bridge cells already resulted in reduction of chromaffin cells, which we detect even at later timepoints. Overall, the lifetime of a bridge cell is short (see ref 7), and that explains the discrepancy between the stages.

We added the following text to the Results section for providing the relevant explanations:

"Delayed cell cycle progression resulted in the reduced number of "bridge" cells observed at this stage, soon after the injection (Fig. 4b). Such reduction of "bridge" cells was not observed at E13.5, due to a fast turnover of "bridge" cells and subsequent recruitment of new "bridge" cells from SCPs. Moreover, 5HTP and 5HT are rapidly depleted after the injection-dependent concentration peak due to pharmacokinetics. At the same time, the dynamics of cell cycle progression in SCPs and early chromaffin cells in AM did not change in control vs treatment group (Fig. 4c). The majority of mature chromaffin cells were negative for EdU or CldU, as they temporarily exit cell cycle in agreement with previous studies [37]*. Due to fast differentiation of chromaffin cells from progenitors in 14 hours* [7]*, reduction of the progenitor pool has a major effect of chromaffin cell population. A majority of chromaffin cells do not proliferate at E12.5-E13.5 and are unable to compensate for the loss."*

6. The analysis of cells remaining in the cell cycle is a welcome addition to the manuscript. This shows an increase in double +ve cells which could be taken to suggest that cell cycle length is expanded (not expended) in 5HT treated mice. To help the reader it would be good to state that double +ve cells are suggestive of increased cell cycle length.

We are thankful for the reviewer for spotting this error, and we improved the text according to this recommendation.

Please check the updated text:

"Despite the overall numbers of proliferating (incorporating one or both thymidine analogues) cells among SCPs, "bridge" cells, early chromaffin cells or mature chromaffin cells did not show a significant change in comparison with control (Fig. 4b), the length of a cell cycle in "bridge" cells appeared significantly increased in the 5HTP treated group (Fig. 4c-d, Supplementary Fig. 7a)."

7. Typo in Supp Fig 1C

It is now corrected

8. Consider replacing Fig 4F - why is a pressure gauge used?

We thank the reviewer for this comment, as we are interested in improving the readability and intuitive understanding of our scheme. We gave it a though and improved the scheme in this figure, as well as we added new explanatory lines to the corresponding figure. The main purpose of Fig.4F is to summarize the results of the major experiments in mouse by introducing the analogy to feedback loop mechanism between chromaffin cells and bridge cells. The "pressure gauge" measures the paracrine influence of chromaffin cells on bridge cells, which is enhanced (arrow moves to the red zone) in case of extra 5HT being supplied to the system. To make it more intuitive, we made the arrows showing velocity of the process more obvious (thicker line, red color), and we also made the note that V2 is smaller than V1 bigger and of red color. We now introduced an explanatory text directly into a figure 4f:

"*The indication on a measuring device shows the amount of a feedback signaling via 5HT from chromaffin to bridge cells"*.

9. Consider representing the qRT-PCR of Fig 6a as per 6c.

We agree with the reviewer. According to the reviewer's recommendation, we have replaced 6a with a new plot. We still present the qPCR data on a log2 scale, which is preferred in the field, as it helps to visualize the differences in downregulation of gene expression much better in comparison with a linear scale. All log2 fold-change values are presented relative to an arbitrary value (not to the mean expression of SH-SY5Y as previously), as it helps to distinguish individual expression values (points) that would otherwise overlap with x-axis (zero). Please check the new Fig. 6a.

Reviewer #3 (Remarks to the Author):

The authors have considerably improved their manuscript "Serotonin limits generation of chromaffin cells during adrenal organ development". The major revision add substantial mechanistic evidence for the role of 5-HT on bridge cell cycle progression and the regulation of the chromaffin organ size and development. This work contributes important and novel information of relevance to multiple fields including psychiatry and oncology. The authors did a good job in measuring 5-HT levels in 5-HTP treated dams. The addition of aggression and exploratory behavior strengthens the manuscript and the idea that increased 5-HT levels lead to decreased medulla size which ultimately correlates with more explorative and less aggressive behaviors. The work is clearly presented and of high quality, but I still have a final major request regarding the supplementary behavioral data:

The authors refer to supplementary figure 9 and state the following:
"As the elevation of normetanephrine is not observed in females, this might signify the sexual dimorphism in endocrine metabolic systems and might explain the behavioral differences observed in males and females."(lines 413-415). They added: "the observed differences in males and females, as well as the only partial correlations with behavioral data might be due to other systemic regulatory mechanisms, which are not covered by this study." (Lines 417-419). If the authors want to use this sentence, they need to perform a 2-way analysis. As it is right now a between treatment within sexes Mann Whitney test has been performed; however, the sex variable or interaction effect of treatment x sex is not reported nor tested. Thus, these two sentences are without any statistical support. (I suggest removing the data or not even discussing the sexual dimorphism but rather state the actual limitation of these results in detecting a difference. Or the authors can just state that for each individual test they detected a difference in one of the sexes but not draw any conclusion on sexual dimorphism as there is none tested.)

We completely agree with the reviewer and removed all the claims about sexual dimorphism.

Please see improved text of the Results section:

"On the other hand, the concentration of normetanephrine was significantly higher in 5HTP-treated males, suggesting an enhanced catabolism of noradrenaline that is typically observed in major pheochromocytoma subtypes in human[46, 47]. The elevation of normetanephrine is not observed in females. Of note, high metanephrine levels might not always reflect high catecholamine synthesis and following catabolism, as inhibition of monoamine oxidase may also lead to elevated levels of catecholamines/metanephrines, which we cannot rule out even though we consider this scenario unlikely based on the previous literature [48-51]. The system of catecholamines and their metabolism is complex and stretches beyond the production of adrenaline and noradrenaline in chromaffin organs. Therefore, the observed differences in males and females, as well as the only partial correlations with behavioral data might be due to other systemic regulatory mechanisms, which are not covered by this study."

2. The authors still need to report the number of litters used and discuss the use of mouse versus litter as the treatment unit (N)

We understand the concern and provide the explanations below. The exact timing of the embryonic stages varies depending on the exact time of conception and implantation, which can be up to 12h apart. The developmental difference within this time-window can

affect the results and introduce the unwanted noise. Based on our previous experience with embryonic stages, pulling several litters into one comparison can result in much greater variability within the overall group, which can hide the intergroup true differences. Selecting and aligning the perfectly comparable litters requires a lot of mice to be sacrificed without much of necessity. With this in mind, and with the goal to minimize the number of animals used, we focused on the analysis of enough number of individual embryos (3-6) serving as biological replicates for our studies in few aligned litters. For the majority of the experiments, we utilized 1-3 litters per experimental condition.

We added a note to the Method section:

"For all experiments, a single embryo was considered as a biological n, and the embryos from 1-3 litters were used in experiment to comply with the 3R policy about the usage of animals in research. Furthermore, the exact timing of the embryonic development varies depending on the time of conception and embryo implantation, which can be up to 12h apart. The developmental difference within this time-window can affect the results and introduce the unwanted noise into the assessment of developing organs at E8-E14.5 stages. Based on our previous experience with such variation, pulling several litters into one comparison can result in much greater variability within the overall group, which can hide the true differences. Selecting and aligning the perfectly comparable litters requires the unnecessary sacrifice of higher numbers of without much of necessity. With this in mind, and with the goal to minimize the number of animals used, we focused on the analysis of adequate numbers of individual embryos (3-6) serving as biological replicates for our studies. For the majority of the experiments, we utilized 1-3 litters per experimental condition."

Reviewer #4 (Remarks to the Author):

Kameneva and colleagues have made major efforts to address our various concerns by performing several new experiments, including very comprehensive studies involving SmartSeq2 analysis and validation by RNA scope. This allowed the authors to better define SCP-derived 'bridge cells' and further strengthen their model on a regulatory feedback loop controlling the generation of chromaffin cells. Interestingly, according to their new data, genes potentially involved in regulating cell cycle length were found to be differentially spliced in the bridge cells. This finding as such is highly interesting and could be addressed in future experiments.

Likewise, the link of their data from developmental processes to neuroblastoma formation has been substantiated by new experiments and by reference to most recently published findings by others that support the authors' conclusion.

I appreciate that the authors very carefully addressed our comments in detail and feel that the study has been greatly improved.

There is only one issue that the authors should comment on: they observed less numbers of bridge cells upon 5HTP treatment in their cell cycle dynamics experiment, whereas no reduction can be seen in Figure 3. Why is this? They should discuss this discrepancy as one would expect to see a reduction in bridge cells in the experiments shown in Figure 3 as well.

We agree with the reviewer, and we provided the relevant explanation. The difference in bridge cell numbers at E12.5 and E13.5 can be explained by (1) the time when the samples were analysed after 5HTP injection because of (2) rapid 5HTP and 5HT reduction (pharmacokinetics) after reaching their peak due to injection, and (3) the speed of bridge cell turnover and differentiation into chromaffin cells (La Manno et al., 2018, Supplementary note 2, Section 6). In the experiment addressing cell cycle dynamics, the analysis of cell populations was done 8 hours after the 5HTP injection. In the analysis of cell populations at E13.5, cell counts were done at 24 hours after the injection. Soon upon the injection of 5HTP, the currently existing bridge cells become affected, and the resulting chromaffin cells generated within 14 hours-time-window appear reduced in their numbers. However, the process of the recruitment of the new bridge cells from SCPs is not affected or stopped. 5HTP, being injected at E12.5, and the corresponding elevation of 5HT are likely weakened by the time when the new bridge cells are recruited during later stages. By E13.5, new bridge cells are recruited from SCPs and those are not that much affected by the transient 5HTP and 5HT elevation, and their numbers appear similar in treatment vs control at E13.5. Still, the earlier, near-immediate defects in bridge cells already resulted in reduction of chromaffin cells, which we detect even at later timepoints. Overall, the lifetime of a bridge cell is short (see ref 7), and that explains the discrepancy between the stages.

We added the following text to the Results section for providing the relevant explanations:

"Delayed cell cycle progression resulted in the reduced number of "bridge" cells observed at this stage, soon after the injection (Fig. 4b). Such reduction of "bridge" cells was not observed at E13.5, due to a fast turnover of "bridge" cells and subsequent recruitment of new "bridge" cells from SCPs. Moreover, 5HTP and 5HT are rapidly depleted after the injection-dependent concentration peak due to pharmacokinetics. At the same time, the dynamics of cell cycle progression in SCPs and early chromaffin cells in AM did not

change in control vs treatment group (Fig. 4c). The majority of mature chromaffin cells were negative for EdU or CldU, as they temporarily exit cell cycle in agreement with previous studies [37]. Due to fast differentiation of chromaffin cells from progenitors in 14 hours[7], reduction of the progenitor pool has a major effect of chromaffin cell population. A majority of chromaffin cells do not proliferate at E12.5-E13.5 and are unable to compensate for the loss."

Reviewer #5 (Remarks to the Author):

This is a lovely developmental biology paper, which deserves to be published. However, it does have two major issues:

- The experimental evidence for the 5HT mediated mechanism for suppressing chromaffin cell production relies entirely on animal experiments, for obvious reasons. However, there is no serious attempt to check if human data is consistent with the story they present. For example, a superficial check of the numerous single cell fetal adrenal atlases suggests that in humans, HTR3A is not expressed in bridge cells. Can feedback from 5HT still be the mechanism for regulating chromaffin cell production in humans given this? It is not clear to me that it can. There is good reason to expect that human adrenal glands will differ from mouse. Recent single cell studies of the human adrenal gland and neuroblastoma (see Dong et al. Cancer Cell, 2020 and subsequent commentary) have demonstrated that human and mouse adrenal glands differ with respect to the key genes they express. To me there is a section missing that connect the mechanism they have established in rodents to what is known about the expression of the key genes (such as HTR3A) in the human adrenal medulla.

We are very much grateful for this comment. Indeed, understanding the human expression of *HTR3A* would be an essential addition to our story. For this, we reanalyzed the most excellent developmental human adrenal gland datasets (single cell transcriptomics) recently published by Jansky et al., 2021 (accessible by the following link https://adrenal.kitz-heidelberg.de/developmental_programs_NB_viz/) to obtain the insight about human aspects of *HTR3A* expression (please see new Supplementary Figure 2).

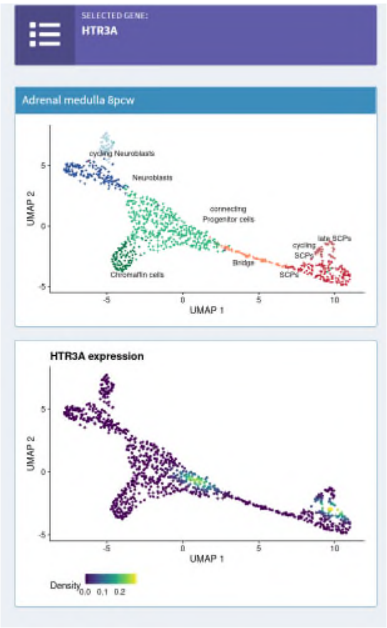

Visualizing the expression of *HTR3A* as kernel density clearly shows that human *HTR3A* is indeed associated with the sympathetic neuroblast lineage, as well as with the subset of SCPs and connecting progenitors to bridge cells specifically at week 8.5 of human adrenal medulla (which would be the equivalent to E12-E13 of a mouse embryonic development). Therefore, *HTR3A* is expressed along human sympathoadrenal lineage

differentiation trajectory in a way similar to mouse *Htr3a* expression in bridge cells, and, thus, can be a part of a feedback loop mechanism in humans. We added these re-analysis data to the supplementary Figure 2 and discussed it in Results sections and Discussion.

Results:

"*Recent profiling of human sympathoadrenal development with single cell RNA sequencing* [22] *revealed that HTR3A is expressed in SCPs and connecting Progenitor cells (next to the "bridge" cells) at 8 weeks post-conception in adrenal medulla (Supplementary Fig.2) as well as in human sympathoblasts at later stages* [22]*.*"

Discussion:

"In humans, the expression of HTR3A revealed a conceptually similar, although somewhat distinct expression pattern. HTR3A appeared expressed in human SCPs and connecting progenitor cells (next to the "bridge" population) at week 8.5 and later in sympathetic neuroblast lineage [22]*. Therefore, the human-specific role of the described paracrine regulation via 5HT and chromaffin progenitor-specific HTR3A is probable.*

- On a somewhat related note the section on neuroblastoma is not very compelling. I do not think it would should be removed entirely, but the commentary surrounding it should be toned down. I would strongly encourage the removal of the speculation regarding the origins of neuroblastoma. In particular these statements should be removed:

- "*These are key characteristics of cancer stem cells, and this observation may point to the previously unrecognized cell states responsible for tumor aggressiveness, in line with recent findings of similarity of genetic profiles of HTR3A+ "bridge" cells and neuroblastoma subpopulations8-10.*"

- "*In line with this reasoning, recent studies showed that the initial stages of chromaffin cell development depend on the recruitment of local nerve-associated Schwann cell precursors (SCPs), which turn into a short-living transient population of "bridge" cells that rapidly transitions towards mature chromaffin cells in mouse and human embryos 6-8. This finding complicated the old picture of the adrenal development (where migratory neural crest cells immediately generate chromaffin tissues), and raised a series of questions regarding the control of the number of chromaffin progenitors operating during the differentiation steps vulnerable to initiation of sympathoadrenal tumors (neuroblastoma, paraganglioma and pheochromocytoma). Moreover, another recent study suggested the involvement of "bridge" chromaffin progenitors in neuroblastoma initiation 9, 10 . These "bridge" cells are characterized by the expression of HTR3A – a co-receptor to serotonin 5-hydroxytryptamine (5HT). Based on that, 5HT was recently hinted to be a part of the mechanism related to the development of adrenal medulla 6.*"

We agree with the reviewer and we removed all speculation regarding the origin of neuroblastoma.

Please check the revised text in introduction:

"In line with this reasoning, recent studies showed that the initial stages of chromaffin cell development depend on the recruitment of local nerve-associated Schwann cell precursors (SCPs), which turn into a short-living transient population of "bridge" cells that rapidly transitions towards mature chromaffin cells in mouse and human embryos [6-9]*.*

This finding complicated the old picture of adrenal development (where migratory neural crest cells immediately generate chromaffin tissues), and raised a series of questions regarding the control of the number of chromaffin progenitors operating during the differentiation steps. These "bridge" cells are characterized by the expression of Htr3a[6, 8] *– a gene encoding for a subunit of HTR3 receptor to serotonin (5-hydroxytryptamine, 5HT). Based on that, 5HT was recently hinted to be a part of the mechanism related to the development of adrenal medulla* [6]*."*

And in discussion:

"The comparison of human HTR3A^{high} and HTR3A^{low} neuroblastoma cell lines revealed that cell lines with HTR3A^{high} expression level have higher tumor-initiating potential. Those cell lines had key characteristics of cancer stem cells and appeared tumorigenic in a mouse xenograft model system as well as formed significantly more spheres in vitro."

- Really all the data show is that there is a subset of neuroblastoma cell lines that express HTR3A and respond to HTR3A agonists. The origins of neuroblastoma are debated (to put it mildly) and the fact that this one gene is expressed on a subset of cell lines does not add productively to this debate. Even if they were to discuss the origins of neuroblastoma (which they should not given their data), the expression of HTR3A is more consistent with a neuroblastic cell of origin given that HTR3A is most strongly expressed by neuroblastic cells, not bridge cells, in the human fetal adrenal medulla (see above, Janksy et al 2021). That neuroblastomas mostly resemble neuroblastic cells is also the consensus from all the recent single cell neuroblastoma studies.

We agree with the reviewer that the neuroblastoma origin is debatable and enigmatic and that our data are still far-fetched. For this reason, we deleted the exact mentioned passages and only say that:

"*Still, it might be wise to keep in mind the potential difference between tumor and healthy HTR3A^{+} cells, as the tumor cells might have additional, unpredictable effects following from HTR3A activation, and the relevance of 5HT paracrine regulation within tumors remains to be elucidated. Moreover, the origin of neuroblastoma is highly debatable* [9, 22, 72-75]*, and our results regarding the feedback loop mechanism involving 5HT and HTR3A in bridge cells in vivo and in cancer cell lines should be interpreted with great care.*"

REVIEWERS' COMMENTS

Reviewer #5 (Remarks to the Author):

The authors have addressed my concerns, with the exception of the expression of HTR3A in humans. The authors provide a screenshot of HTR3A from https://adrenal.kitz-heidelberg.de/developmental_programs_NB_viz/ claiming this supports the mechanism being the same in humans. I used this same atlas to check it's expression and express scepticism that HTR3A follows the same pattern in humans. If the gene is plotted without "kernel density" on, expression of HTR3A is not detectable.
It seems likely that the kernel view the authors show represents expression of one or two reads in one or two cells, barely more than noise. The same kernel plot applied to the whole adrenal medulla also shows that the expression of HTR3A in humans is mostly in late neuroblasts, no the bridge cells.
Another human adrenal gland atlas (https://www.neuroblastomacellatlas.org/all) similarly shows that HTR3A is not expressed in bridge cells in humans and only in sympathoblasts.
Given this, I do not think that the added paragraph in the results is a fair representation of the data relating to human HTR3A and should be changed accordingly.

REVIEWERS' COMMENTS

Reviewer #5 (Remarks to the Author):

The authors have addressed my concerns, with the exception of the expression of HTR3A in humans. The authors provide a screenshot of HTR3A from https://adrenal.kitz-heidelberg.de/developmental_programs_NB_viz/ claiming this supports the mechanism being the same in humans. I used this same atlas to check it's expression and express scepticism that HTR3A follows the same pattern in humans. If the gene is plotted without "kernel density" on, expression of HTR3A is not detectable. It seems likely that the kernel view the authors show represents expression of one or two reads in one or two cells, barely more than noise. The same kernel plot applied to the whole adrenal medulla also shows that the expression of HTR3A in humans is mostly in late neuroblasts, no the bridge cells. Another human adrenal gland atlas (https://www.neuroblastomacellatlas.org/all) similarly shows that HTR3A is not expressed in bridge cells in humans and only in sympathoblasts. Given this, I do not think that the added paragraph in the results is a fair representation of the data relating to human HTR3A and should be changed accordingly.

- We are very much thankful to the reviewer because indeed the kernel density view appeared extraordinary misleading. As we trusted the online interface without re-running the data, we were tricked into this situation. To respond to this problem and to clarify whether the expression of *HTR3A* is truly present in human "bridge" cells, we performed our own, a bit deeper 10X sequencing of developing human adrenal glands at weeks 5-to-7. Our new result showed the specific expression in "bridge" cells and in sympathoblasts despite the number of positive "bridge" cells was low. Generally, the expression level of *HTR3A* appeared low in both "bridge" cells and sympathoblasts (around 2 UMI). However, the specificity of this low expression per specific cluster (validated with Fisher test) suggested that this expression is real, although close to the border of detection (please see the updated Supplementary Figure 2c). To clarify this, we performed an experimental validation with *HTR3A* RNAscope probe and found labeled cells with 1-4 dots per cell on average, confirming the low level of specific *HTR3A* expression in developing human adrenal gland (please see the updated Supplementary Figure 2d). Some of *HTR3A* positive cells also showed positive *PENK* RNA signal (manifesting chromaffin cell differentiation), which nicely corresponded to the location on the UMAP embedding where bridge cells start to upregulate *PENK* and transit into chromaffin population (please see the updated Supplementary Figure 2).
  Previously, we relied on Smartseq2 sequencing for detecting *Htr3a* expression in mice, and Smartseq2 is a much more sensitive method as compared to 10x Genomics. The

technical complications prevented us from using Smartseq2 for sequencing human cells this time and reliably concluding on robust expression of *HTR3A* in human "bridge" cells. Therefore, although we show our new positive data, we keep this question open without pushing for the importance of HTR3A in human "bridge" cells. We made a neutral and open description of these results, also mentioning rather negative data from other groups, which you can find in the updated Results section and also in the Discussion:

Results:

"To check if HTR3A is expressed in human adrenal medulla during development, we isolated adrenal glands at weeks 5-to-7 and subjected them to single cell transcriptomics analysis with Chromium 10X approach. The results showed the sparse expression of HTR3A in "bridge" cells and rather consistent expression in sympathoblasts (Supplementary Fig. 2a-b). Although we detected only 6 HTR3A^{+} cells in a "bridge" population, the statistical test supports the significance of this find (Supplementary Fig. 2c). At the same time, this suggests low expression level of HTR3A, which we experimentally validated with RNAscope on slices of week 6 and week 8 human adrenal tissue (Supplementary Fig. 2d). Indeed, if the expression of HTR3A is truly present, it is weak and at the border of detection, which leaves the question about the role of HTR3A in human "bridge" cells open. Consistently, the data from other groups show almost absent expression of HTR3A from SCPs and "bridge" cells in developing human adrenal medulla despite its clear presence in sympathoblasts[22]. Again, a question of whether HTR3A is sufficiently present in "bridge" cells of human adrenal glands requires further investigation with more sensitive methods.''

Discussion:

"In humans, the sparse expression of HTR3A appeared in "bridge" population at weeks 5-to-7 according to RNAseq and at weeks 6 and 8 according to experimental validations with RNAscope. Despite the expression was detected in principle, it appeared low and at the border of detection, leaving a question about the role of HTR3A in human "bridge" cells open. Contrary to this, the expression of HTR3A in sympathoblasts showed a strong and consistent pattern, also in agreement with other studies 22. Therefore, the human-specific role of the described paracrine regulation via 5HT and chromaffin progenitor-specific HTR3A is probable, although it requires further experimentation to be validated."

We hope that the Reviewer will appreciate the new experimental effort during the 3rd revision round and will find these updates open, fair and appropriate.
